# Pulmonary siRNA Delivery with Sophisticated Amphiphilic Poly(Spermine Acrylamides) for the Treatment of Lung Fibrosis

**DOI:** 10.1002/smll.202308775

**Published:** 2023-12-21

**Authors:** Friederike Adams, Christoph M. Zimmermann, Domizia Baldassi, Thomas M. Pehl, Philipp Weingarten, Iris Kachel, Moritz Kränzlein, David C. Jürgens, Peter Braubach, Ioannis Alexopoulos, Malgorzata Wygrecka, Olivia M. Merkel

**Affiliations:** Pharmaceutical Technology and Biopharmaceutics, Department Pharmacy Ludwig-Maximilians-University Munich, Butenandtstr. 5−13, 81377 Munich, Germany; Institute of Polymer Chemistry Chair of Macromolecular Materials and Fiber Chemistry, University of Stuttgart, Pfaffenwaldring 55, 70569 Stuttgart, Germany; Center for Ophthalmology University Eye Hospital Tübingen, Elfriede-Aulhorn-Straße 7, 72076 Tübingen, Germany; Pharmaceutical Technology and Biopharmaceutics, Department Pharmacy Ludwig-Maximilians-University Munich, Butenandtstr. 5−13, 81377 Munich, Germany; WACKER-Chair of Macromolecular Chemistry, Catalysis Research Center, Department of Chemistry, Technical University Munich, Lichtenbergstr. 4, 85748 Garching bei München, Germany; Pharmaceutical Technology and Biopharmaceutics, Department Pharmacy Ludwig-Maximilians-University Munich, Butenandtstr. 5−13, 81377 Munich, Germany; WACKER-Chair of Macromolecular Chemistry, Catalysis Research Center, Department of Chemistry, Technical University Munich, Lichtenbergstr. 4, 85748 Garching bei München, Germany; Pharmaceutical Technology and Biopharmaceutics, Department Pharmacy Ludwig-Maximilians-University Munich, Butenandtstr. 5−13, 81377 Munich, Germany; Institute for Pathology Hannover Medical School, Carl-Neuberg-Straße 1, 30625 Hanover, Germany; Biomedical Research in Endstage and Obstructive Lung Disease, Hannover (BREATH) Research Network, Member of the German Center for Lung Research (DZL), Hannover Medical School, Carl-Neuberg-Str. 1, 30625 Hanover, Germany; Center for Infections and Genomics of the Lung (CIGL), Justus Liebig University Giessen, German Center for Lung Research, Aulweg 132, 35392 Gießen, Germany; Multiscale Imaging Platform Institute for Lung Health German Center for Lung Research, Aulweg 132, 35392 Giessen, Germany; Center for Infections and Genomics of the Lung (CIGL), Justus Liebig University Giessen, German Center for Lung Research, Aulweg 132, 35392 Gießen, Germany; Pharmaceutical Technology and Biopharmaceutics, Department Pharmacy Ludwig-Maximilians-University Munich, Butenandtstr. 5−13, 81377 Munich, Germany

**Keywords:** lung fibrosis, pulmonary delivery, poly(spermine acrylamide), post-polymerization functionalization, siRNA delivery

## Abstract

RNA interference (RNAi) is an efficient strategy to post-transcriptionally silence gene expression. While all siRNA drugs on the market target the liver, the lung offers a variety of currently undruggable targets, which can potentially be treated with RNA therapeutics. To achieve this goal, the synthesis of poly(spermine acrylamides) (P(SpAA) is reported herein. Polymers are prepared via polymerization of N-acryloxysuccinimide (NAS) and afterward this active ester is converted into spermine-based pendant groups. Copolymerizations with decylacrylamide are employed to increase the hydrophobicity of the polymers. After deprotection, polymers show excellent siRNA encapsulation to obtain perfectly sized polyplexes at very low polymer/RNA ratios. In vitro 2D and 3D cell culture, ex vivo and in vivo experiments reveal superior properties of amphiphilic spermine-copolymers with respect to delivery of siRNA to lung cells in comparison to commonly used lipid-based transfection agents. In line with the in vitro results, siRNA delivery to human lung explants confirm more efficient gene silencing of protease-activated receptor 2 (PAR2), a G protein-coupled receptor involved in fibrosis. This study reveals the importance of the balance between efficient polyplex formation, cellular uptake, gene knockdown, and toxicity for efficient siRNA delivery in vitro, in vivo, and in fibrotic human lung tissue ex vivo.

## 1 Introduction

RNA mediated targeted inactivation of gene expression, known as RNA interference (RNAi), is an established technique for the investigation of cellular processes in vivo and is increasingly being suggested for the potential treatment of a plurality of diseases such as respiratory disorders (e.g., severe acute respiratory syndrome viruses or asthma),^[1–8]^ rheumatic diseases,^[9,10]^ brain diseases,^[11–15]^ or degenerative disorders.^[16,17]^ The G protein-coupled receptor protease-activated receptor (PAR)−2 is an emerging new target in fibrosis,^[18]^ and the PAR-2/tissue factor/factor VIIa (PAR-2/TF/FVIIa) axis was earlier identified to contribute to the development of pulmonary fibrosis specifically.^[19]^ Since PAR-2 is a molecular hub in lung fibrosis, it is an ideal candidate for pharmacological inhibition or therapeutic intervention using siRNA for specific gene silencing.^[20]^ Investigation of precision-cut lung slices (PCLS) from fibrosis patients can bridge a gap between in vitro and in vivo models and is a reliable and powerful model for studying respiratory diseases and therapeutic approaches. PCLS are ideal for high-resolution live imaging. Structural changes and drug treatments can be tracked using fluorescent markers for different cell types, cell signals, or other processes. Especially, human precision-cut lung slices (hPCLS) can provide highly relevant data for human diseases, eliminating the often uncertain extrapolation of results from animal models to humans.^[21]^ Since artificially synthesized siRNAs can be introduced into cells by transfection, in principle sequence-specific cleavage of any target mRNA can be achieved via a complementary siRNA.^[22–27]^ Despite intensive research, to date, the FDA has approved only five siRNA drugs: Onpattro/patisiran, Givlaari/givosiran, Oxlumo/lumasiran, Leqvio/inclisiran, and Amvuttra/vutrisiran; all targeting liver-specific diseases, which shows the lack of current therapy in specific targeting of siRNA therapeutics beyond the liver, especially when administered intravenously.^[28–30]^ Local administration routes can improve the performance of in vivo RNAi. Intranasal or intratracheal delivery to the airway and alveolar epithelium can enhance the delivery efficiency of siRNA regarding respiratory disorders without the need for active targeting combined with the advantage of reduced doses compared to systemic administration and the avoidance of the first-pass metabolism. Thus, less systemic side effects are expected, and interactions with serum proteins can be avoided. Due to administration locally to the nose or mouth, direct access to target lung epithelial cells is facilitated that are essential target cell types in a variety of pulmonary diseases (e.g., cystic or pulmonary fibrosis, chronic obstructive pulmonary disease, asthma, lung cancer, influenza or SARS corona viruses).^[31–33]^ Lung diseases are a major cause of mortality worldwide and need new therapeutic approaches to treat lung-related disorders, however, efficient siRNA delivery to the lungs is very complex.^[34]^ Despite several advantages of pulmonary administration of siRNA, major barriers of respiratory delivery are mucociliary clearance, the presence of mucus, and clearance of particles by macrophages.^[35]^ The use of nanoparticles can help to overcome these limitations. An siRNA system for efficient local administration to the lungs should: 1) Condense siRNA; 2) Protect the payload from degradation; 3) Improve cell uptake; 4) Facilitate endosomal escape; 5) Silence the target gene via siRNA release from the carrier; 6) Show biocompatibility. In the context of pulmonary delivery, polymers can be advantageous over lipid-based non-viral vectors to protect and transport negatively charged siRNA due to their higher encapsulation efficiencies of nucleic acids, combined with a better reproducibility of the formed siRNA/polymer-complexes (polyplexes) and their improved stability in lung surfactants.^[35,36]^ In addition, these polycationic polymers can be synthesized on a large scale and generally exhibit higher biocompatibility than viral vectors.^[37,38]^ A plethora of different cationic polymers have been designed with the above-mentioned criteria (e.g., polyethylenimine (PEI) and several modifications of PEI, chitosan, cationic dendrimers. polylysines, etc.) none of which of combine all aspects of efficient pulmonary siRNA delivery. Especially in pulmonary fibrosis treatment, siRNA/polymer systems have not been sufficiently tested and siRNA silencing of PAR-2 with polyplexes for lung-related disorders has not been investigated so far. Recent developments with a focus on other targets in lung fibrosis treatment include naked siRNA,^[2]^ PEGylated poly(dimethylamino)ethylmethacrylate,^[39]^ self-assembled micelle inhibitory RNA nanoparticles,^[40]^ PEI derivatives,^[41,42]^ perfluorocarbon emulsion polyplexes,^[43]^ guanidinium-functionalized poly(oxanorbornene)imide^[44]^ and liposomes.^[45]^ Simple cationic polyplexes are not capable of efficiently transiting through mucus most likely due to the highly negative charge of the lung mucus layer.^[34]^ The development of polymers with amphiphilic properties to stabilize polyplexes in the mucus and simultaneously overcoming low transfection efficiencies of non-viral systems combined with minimal cytotoxicity remains to be the greatest barrier of successful non-viral nucleic acid delivery to treat lung fibrosis.^[38,46]^ Spermine is a biogenic polyamine with great potential in nucleic delivery as it is found as a polycation at all pH values, however, it shows only low ability to condense siRNA due to its low molecular weight. It is produced biosynthetically from the amino acids arginine and ornithine via polycationic intermediates putrescine and spermidine regulated by several enzyme classes such as oxidases, transferases, synthases, and decarboxylases, making spermine-containing polymers potentially biodegradable.^[47–50]^ Although the first gene transfer in mammalian cells was performed by Szybalksa and Szybalksi in 1962 with spermine, spermine polymers remain rare in literature.^[51]^ To the best of our knowledge, spermine-containing polymers for RNA delivery are restricted to oligomeric and low-molecular weight forms,^[52–59]^ modification of precursors with low numbers of spermine molecules^[60,61]^ or linear spermine-polymers that solely bear secondary amines^[62–66]^ hindering efficient nucleic acid delivery.

Since high molecular weight and branched polymers often have beneficial transfection efficiencies, we herein report on novel high molecular weight, brushed spermine-based polyacrylamides synthesized via facile free-radical polymerization of N-acryloxysuccinimide (NAS), an active ester monomer. Post-polymerization functionalization with a modified spermine-species and deprotection led to poly(spermine acrylamides) P(SpAA) with increased molecular-weights that still contain a high density of secondary and primary amines.

To investigate the impact of different hydrophobic fractions on siRNA delivery especially when mucus is present, copolymers with varying ratios of spermine acrylamide (SpAA) units (cationic) and hydrophobic units, derived from N-decylacrylamide (DAA), were synthesized. This hydrophobic monomer, which has an excellent balance of hydrophobicity and size, has received little attention for drug delivery applications. Only very few reports are published in the current literature in which DAA was used for micellar approaches.^[67–69]^ This modification was used to boost the non-specific cellular uptake, by binding of amphiphilic compounds to lipid membranes that can consequently also result in higher transfection efficiencies.^[37,70,71]^ Additionally, hydrophobic modifications with alkyl chains have been reported to decrease cytotoxicity of cationic polymers.^[72,73]^

The obtained homopolymers P(SpAA) 1−3 and copolymers P(SpAA-co-DAA) 1−3 showed different molecular weights and molar ratios of DAA ranging from 0 to 60 mol%. Polymers were fully characterized and achieved excellent siRNA encapsulation to obtain perfectly sized polyplexes at low polymer excess. In vitro 2D and 3D cell culture experiments, ex vivo gene silencing in human lung explants from patients suffering from lung fibrosis and in vivo experiments revealed superior properties of amphiphilic P(SpAA-co-DAA) in delivery of siRNA to lung cells showing a perfect balance between polyplex formation, toxicity, and siRNA delivery efficiency thus fulfilling all criteria for efficient pulmonary siRNA delivery to treat fibrosis.

## 2 Results and Discussion

### 2.1 Polymerization Studies

Monomers NAS 1 and N-decylacrylamide 2 (DAA) were synthesized starting from acryloyl chloride, triethylamine and N-hydroxysuccinimide or N-decylamine, respectively, using modified literature procedures.^[67,74]^ Both monomers were isolated as pure products in good yields (71% NAS, 65% DAA).

To use spermine as a functional molecule, tri-boc spermine **3** (TBSp) that bears only one reactive primary amine was synthesized using orthogonal protection group chemistry in an adapted literature procedure.^[75]^ Such a complex synthesis route is mandatory to prevent possible unwanted side reactions, cross-linking, and to facilitate the reaction of solely one amine group per spermine during post-polymerization functionalization.

**Scheme 1:**
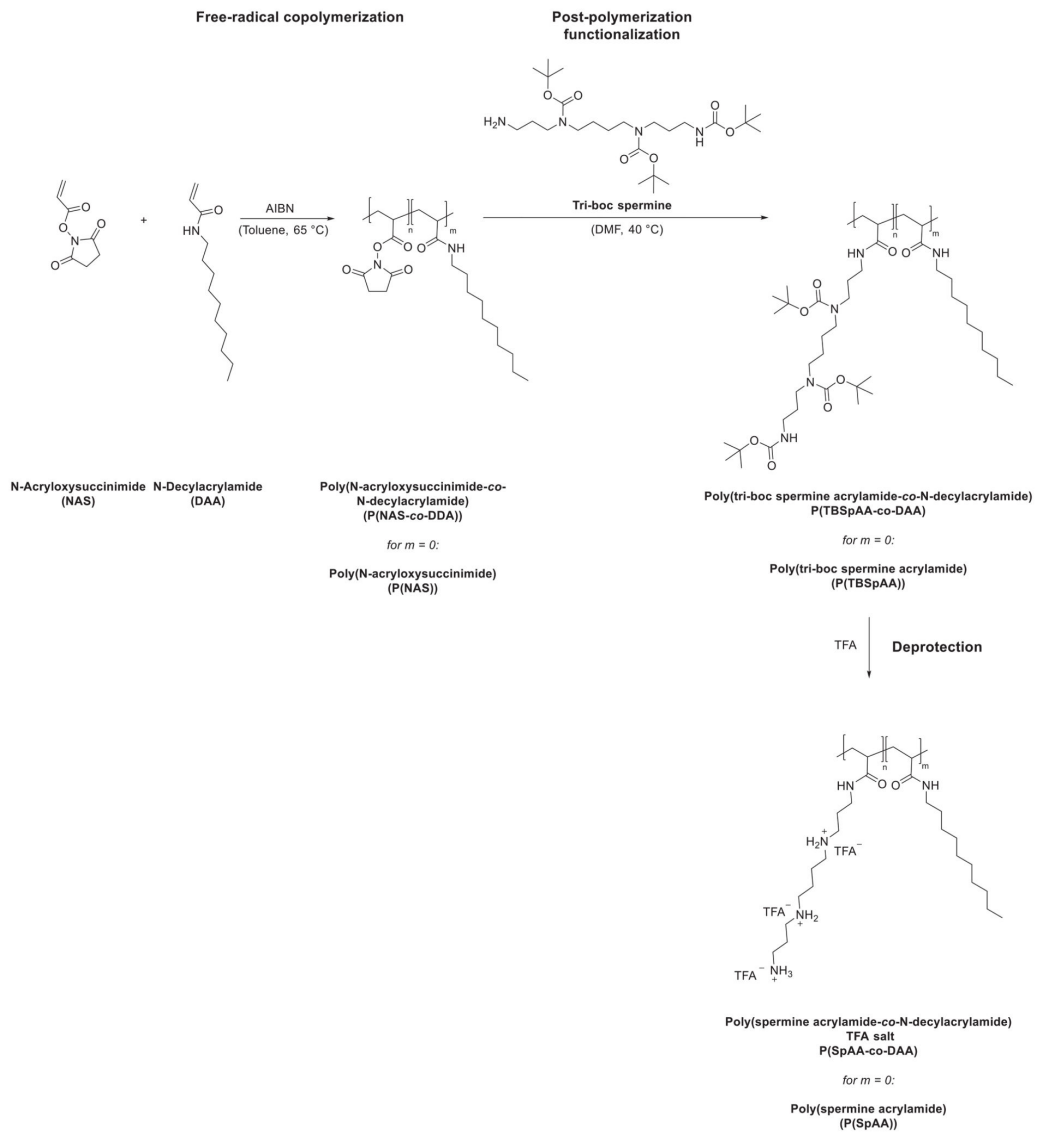
Synthesis of poly(spermine acrylamide) (P(SpAA)) and poly(spermine acrylamide-co-N-decylacrylamide) (P(SpAA-co-DAA) via radical (co)polymerization of N-acryloxysuccinimide (NAS) 1 and N-decylacrylamide (DAA) 2, followed by post-polymerization with tri-boc spermine 3 and deprotection using tri fluoro acetic acid (TFA).

The endogenous molecule spermine seems to have great potential in encapsulating siRNA and therefore, presents a promising non-viral gene delivery agent. In order to increase its molecular weight, spermine homo- and copolymers were synthesized via free radical polymerization (FRP). Using NAS as a precursor monomer and DAA as the hydrophobic unit, these monomers were employed in FRP using azobisisobutyronitrile (AIBN) as polymerization initiator to evaluate the general activities, yields, molar masses, polydispersities, and microstructures of the isolated poly(N-acryloxysuccinimide) (P(NAS)) and poly(N-decylacrylamide) (P(DAA)) homo- and copolymers (Scheme 1). NAS polymerization was conducted over night at 65 °C in toluene using monomer concentrations of 10 wt./vol.% in the presence of varying amounts of AIBN (2−10 wt%) as the radical starter. No clear correlation between the weight ratio of AIBN and molecular weight during polymerization was observed (Table 1). As expected for FRP and due to precipitation of the polymer during polymerization, the obtained P(NAS) polymers 1−3 showed molar masses between 12.6 and 20.0 kg mol^−1^ and broad molecular weight distributions (3.43 ≤ Ð ≤ 3.57) measured via size-exclusion chromatography (SEC). All polymers were isolated in good yields (84 − 97%). A similar procedure was used for DAA polymerization. Using 2 wt% AIBN, P(DAA) with a molar mass of 23.7 kg mol^−1^ and a polydispersity of 2.02 was synthesized. Additionally, copolymers with varying ratios of NAS and DAA were synthesized by simple modulation of the monomer feed by preaddition mixing of different NAS/DAA ratios using 10 wt% AIBN and a total monomer concentration of 10 wt./vol.%. Different ratios of NAS and DAA contents were obtained in the copolymers P(NAS-co-DAA) 1−3 ranging from 90 mol% NAS to 50 mol% NAS (Table 1). ^1^H NMR spectroscopy verified the successful polymerization since all signals of the NAS and DAA repeating units agree with those of the obtained homopolymers P(NAS) and P(DAA) (Figures S11 and S15, Supporting Information). It was possible to exactly tune the composition of the copolymer through the monomer feed as the ratios of the two monomers in the feed and in the polymer were nearly identical (Table 1). DOSY NMR studies were used to confirm the successful linkage between the NAS and the DAA monomers and to exclude the formation of two separate homopolymers. DOSY NMR spectra of P(NAS-co-DAA)2 showed only one set of signals assigned to the diffusion coefficient for the copolymer (Figure S18, Supporting Information). Compared to homopolymerizations, the shift in molecular weight of the copolymers with increasing DAA content can either be attributed to higher molecular weights or to different hydrodynamic radii of DAA containing polymers. To further understand the comonomer sequence distribution and the compositional drift of the copolymers, since material properties strongly correlate to the molecular structure of copolymers, a series of reactions with different monomer feed ratios was performed. Fineman−Ross technique was used to determine the monomer reactivity ratios of NAS (r_1_ (r_NAS_)) and DAA (r_2_ (r_DAA_)) in copolymerizations (Figure S10, Supporting Information).^[76,77]^ r_1_ (r_NAS_) was calculated as 0.76, showing a minor preference for cross-propagation, whereas r_2_ (r_DAA_) had a value of 1.39, indicating a preference for homopropagation. Consequently, a compositional drift from DAA to NAS occurred. If higher fractions of DAA are incorporated in the initial stage of the copolymerization, this molecular structure could be beneficial for amphiphilic material properties.

### 2.2 Post-Polymerization Functionalization

Post-polymerization functionalization is a facile tool for access to pendant groups that are not easily accessible as monomers themselves. NAS, an active ester of acrylic acid, was chosen as a monomer, because active esters are reactive toward nucleophiles in a straightforward addition−elimination reaction without generating toxic by-products. After polymerization of NAS or copolymerization with DAA, the obtained homo- and copolymers were treated with one equivalent of TBSp **3** per NAS-repeating unit to convert NAS units to tri-boc spermine acrylamide (TBSpAA). To ensure full removal of active esters to prevent side reactions during in vivo and in vitro evaluations, the reaction mixture was treated with ammonia to convert unreacted NAS units to water-soluble acrylamides. Thus, proportions of the polymers consist of acrylamide repeating units generating a di-or terpolymer, respectively (repeating unit of acrylamide omitted for clarity). Signals could be assigned to either TBSp pendant group, DAA, or back-bone protons. Additionally, no residual N-hydroxysuccinimide, which is formed during modification of P(NAS), is intercalated in the polymer, which was often observed if ammonia was not added. SEC analysis in DMF showed a shift in retention time toward higher molecular weights for all polymers. Molecular weights were not comparable between P(NAS) and P(TBSpAA) homo- and copolymers, due to potentially different hydrodynamic radii in the SEC solvent and broad polydispersities.^1^H NMR spectroscopy was also used to determine the percentage of the TBSpAA and DAA in the obtained copolymers. The calculated ratio of spermine in P(TBSpAA-co-DAA) 1−3 showed slightly divergent values than the NAS/DAA ratios (Table 1). This is explained by the low amount of NAS groups that was converted into acrylamides instead of being functionalizedby spermine.

Deprotection of all Boc-protected polymers was performed in trifluoroacetic acid (TFA) to obtain the TFA salts of the desired (P(SpAA) and poly(spermine acrylamide-co-DAA) (P(SpAA-*co*-DAA)) polymers. Due to overlap of the backbone protons, the exact amount of acrylamide in the homopolymers could not be calculated. For the copolymers (i.e., terpolymers), an estimated amount of acrylamide between 1−10 wt% was calculated based on the change in the ratio from NAS/DAA to SpAA/DAA (Table 1). Due to the low molecular weight of the acrylamide units in comparison to the other two units in the polymer, all other calculations are negligibly affected by the amount of acrylamide present in the copolymers.

To be able to compare the polymers to a small molecule with a similar structure (one primary and two secondary amines), TBSp **3** was converted to a propionyl-spermine amide species **4** in a two-step reaction, i.e., coupling to an activated propionyl-group followed by deprotection with TFA. ^1^H NMR spectra of the polymer TFA-salts showed similar chemical shifts of the spermine proton signals as **4** with a strong broadening of the peaks attributed to the polymeric structure (Figures S8 and S13, Supporting Information). Additionally, the methylene group signal of **4** showed a similar chemical shift as the backbone protons of the isolated P(SpAA) polymers 1−3. ^13^C NMR spectra of the polymers reflected the presence of TFA salt in the polymers, substantiating the assumption of polymer TFA-salts (Figure S14, Supporting Information).

After deprotection, a final cationic to hydrophobic ratio (SpAA:DAA) of 83:17, 76:24, and 43:57 was obtained for the copolymers P(SpAA-co-DAA)1, P(SpAA-co-DAA)2, and P(SpAA co-DAA)3. These values are in accordance with the ones before deprotection, showing that TFA treatment does not lead to any alteration of the polymer composition or degradation of the polymer.

### 2.3 Properties of Cationic Polymers

In the past years, different polymers ranging from bio-based polymers such as chitosan and poly(L-lysine) to synthetic ones like PEI and polymethacrylates were tested as non-viral vectors for gene and nucleic acid therapy. Studies on structure-function relationships revealed a necessity of positively charged amines in which the structure and density of amines impacted the transfection efficiency. A higher density of amines and thus a higher charge provoke a higher efficiency, but also a higher toxicity.^[37]^ An important property of polycations is their buffering capacity to facilitate endosomal escape of siRNA. Due to the buffering capacity of amine-containing polymers, endosomes can be destabilized that is often explained by the “proton-sponge effect”. The residual non-protonated amino groups of the polymers after complexation with siRNA are protonated due to a lower pH in the endosome causing the disruption of the membrane, release of the genetic material into the cytoplasm, and successful transfection.^[78,79]^ Amongst the polymers used for nucleic acid delivery, branched PEI is one of the most widely used polymers. It shows a superior transfection efficiency, due to its high buffering capacity caused by its branched polymer architecture. With primary, secondary, and tertiary amino groups exhibiting pKa values distributed over the entire physiological pH range (10 to 4), it still has a good buffering capacity in the acidic environment of endo-lysosomes thus acting as a proton sponge.^[78,79]^ However, as a consequence thereof, it also causes high cytotoxicity, which is associated with limitations and concerns in in vivo applications and with restrictions in clinical trials.^[80]^

The basic capacity of spermine-polymer P(SpAA)3 and spermine-molecule **4**, both with a ratio of primary/secondary/tertiary amines = 1/2/0, was investigated by performing an acid/base titration with 0.1 m HCl (Figure S32, Supporting Information). It was compared to hyperbranched PEI with a molecular weight of 25 kg mol^−1^ and a ratio of primary/secondary/tertiary amines of 1/1.1/0.7 (indicated by the manufacturer, BASF, Ludwigshafen, Germany). Additionally, spermine (primary/secondary/tertiary amines = 2/2/0) was investigated to compare the results with the spermine polymers with respect to changes in basicity when one primary amine is converted to an amide. Polymers with higher buffering capacities required larger amounts of HCl for the alteration of the pH value of the solution. PEI had a substantial buffering capacity over almost the whole pH range. The buffering capacity at the pH values higher than 9 is mainly attributed to the primary and secondary amines with the reported pKa values of 8−9, whereas the buffering capacity at lower pH values, especially in the range of 5.5−7, might be associated to the tertiary amines with the pKa values of 6−7, which is in accordance with literature.^[81]^ Spermine itself is known to induce pH buffering. Since the same amount of substance (in mol) was used regarding each protonable unit, the buffering capacity of P(SpAA)3, **4** and spermine was found to be better than that of PEI in the region of pH 11 to pH 6. The P(SpAA)3 and the modified spermine molecule **4** showed similar buffering capacities, with the latter performing slightly better, revealing the similarity of both structures with regards to the ratio of primary and secondary amines. Having the highest percentage of primary amines, spermine performed best in the pH region of 11−6. Due to the absence of tertiary amines, a lower buffering capability is observed for P(SpAA)3, **4** and spermine in the pH region of 5 to 2.

Considering that side chain coiling of the brushed polymers in polyplexes might further lower the pK_a_ value of the sterically hindered secondary amines, these results suggest that within the experimental pH range of 5−8, spermine polymers offer a buffering ability, which can facilitate endosomal escape.

The critical aggregation concentration (CAC) is defined as the concentration of amphiphilic molecules required for their spontaneous self-assembly of these substances. It is a determining factor for applications of amphiphilic polymers, including drug delivery, as it predicts the stability of self-assembled polymeric aggregates or micelles. Hence, P(SpAA)3 and P(SPAA-co-DAA) 1−3 solutions in water with increasing concentration were prepared. Using the water-insoluble Nile red, a fluorogenic dye that undergoes an increase of fluorescence intensity when encapsulated in the hydrophobic core of a self-assembled system, CAC values were determined by plotting the concentration of the polymer versus the fluorescence of Nile red.^[82]^ Homopolymer P(SpAA)3 was barely able to encapsulate small amounts of Nile Red when using concentrations of 0.1 mg mL^−1^ or more, but only a very low fluorescence was observed (Figure S33, Supporting Information). It seems that the hydrophobic backbone of the polymer led to negligible amphiphilic properties. In contrast, copolymers P(SPAA-co-DAA) 1−3 that bear different percentages of hydrophobic comonomer DAA can encapsulate considerable amounts of Nile Red. In detail, the CAC decreased from 0.24 mg mL^−1^ determined for P(SPAA-co-DAA)1 (17% DAA units) nearly six-fold to 0.041 mg mL^−1^ for P(SPAA-co-DAA)3 bearing 57% hydrophobic monomer units (Figures S34 − S36, Supporting Information). This value is within the same range or even lower than values reported for hydrophobically-modified PEI systems.^[83–87]^ In addition, not only the CAC decreased with increasing amounts of hydrophobic moieties, but also the fluorescence value increased significantly indicating that higher amounts of Nile red were encapsulated. Consequently, copolymers P(SPAA-co-DAA) 1−3 seemed to be promising candidates with amphiphilic properties that can enhance cellular uptake and stability of polyplexes.

### 2.4 Physico-Chemical Characterization of Polyplexes with siRNA

**Figure 1:**
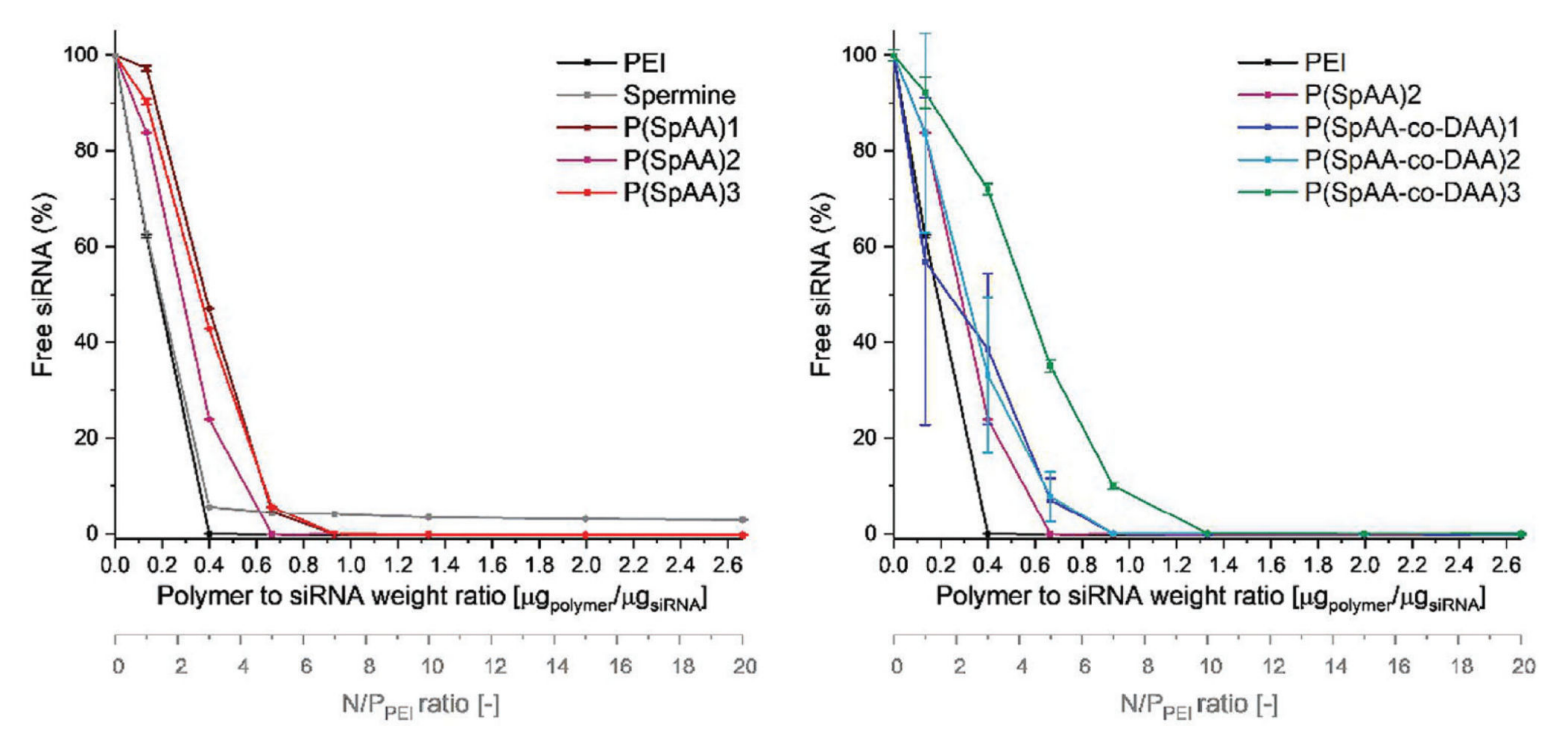
siRNA encapsulation profiles of polyplexes as measured by SYBR Gold assay at various polymer to siRNA weight ratios. 100% values are represented by the determined fluorescence of uncondensed siRNA (data points indicate mean, n = 3).

SYBR Gold assay was used to evaluate the capacity of the polymers to condense siRNA at various concentrations. An excellent condensation of siRNA is needed to protect siRNA and to ensure cellular uptake while preventing enzymatic degradation. The synthesized positively charged polymers can electrostatically interact with negative charges of phosphate groups in siRNA that is quantified using the fluorescent dye SYBR Gold. Apart from P(SpAA)1-3 and P(SpAA-co-DAA) 1−3, hyperbranched PEI (25 kg mol^−1^) and spermine were used for comparison reasons (Figure 1). All polymers and spermine were able to condense siRNA in which more efficient encapsulation was observed with increased concentrations, due to higher amounts of positively charged amines facilitating more electrostatic interactions with siRNA. Spermine showed a relatively low siRNA encapsulation ability with a constant value of ≈4% free siRNA (or detectable siRNA on the surface of the complexes) even at high concentrations attributed to the rigid siRNA-structure and the low molecular weight of spermine compared to the more flexible spermine-pendant groups in the polyacrylamides. Ho-mopolymers P(SpAA)1 and P(SpAA)3 showed full encapsulation at ≈1.0 μg polymer per μg siRNA, whereas P(SpAA)2 showed a slightly better encapsulation. Due to the higher number of amines in PEI using same concentrations, siRNA encapsulation is more efficient leading to full encapsulation at weight ratios above 0.4 μg polymer/μg siRNA. In matters of N/P ratio this would be a value of 3 regarding the protonable unit of PEI that is in accordance with SYBR gold assays of hyperbranched PEI (25 kDa) performed in literature.^[88]^ The N/P ratio is defined as the numbers of amines in the polymer divided by the number of siRNA phosphate groups (calculation see Supporting Information). Regarding that commonly used number, P(SpAA)1-3 show full encapsulation already at N/P of 1, showing the excellent encapsulation ability of these polymers (Figure S37, Supporting Information). An extensive use of free amine groups can be avoided that can provoke high toxicities. In comparison to similar systems, such low N/P ratios for fully condensing siRNA were neither reported for spermine/siRNA complexes^[52,53,58–60,63,64]^ nor for hydrophobically-modified PEI/siRNA systems.^[89–91]^ Such an efficient siRNA can be attributed to the flexible alkyl back-bone complemented by the flexible brushed spermine-side chains with three amino groups in close proximity facilitating optimal encapsulation of rather rigid double stranded siRNA.

Copolymers P(SpAA-co-DAA)1 and P(SpAA-co-DAA)2 showed comparable profiles with a sufficient encapsulation of siRNA at concentrations of 1.0 μg polymer per μg siRNA. Furthermore, the P(SpAA-co-DAA)3 copolymer required a higher concentration for a complete encapsulation of siRNA due to high amounts of hydrophobic subunits that do not contribute to electrostatic interactions between amines and siRNA. siRNA encapsulation based on N/P ratio showed that also copolymers can encapsulate siRNA at an N/P ratio of one or higher (Figure S37, Supporting Information). From these results, it can be concluded that hydrophobic segments in the polymer do not negatively influence electrostatic interactions of siRNA and amines, in contrast to what was observed in literature where hydrophobic modification led to less efficient siRNA encapsulation compared with the unmodified PEI-counterpart at the same N/P ratio.^[89,91]^

Not only the full encapsulation of siRNA, but also size and charge of polyplexes play a major role in successful delivery of the payload. Particle sizes, polydispersity indices (PDI) and zeta potentials of polyplexes were measured using dynamic light scattering (DLS) and laser Doppler anemometry (LDA). Comparing polyplexes, formed with the same concentration of polymers using three different concentrations (c_1_ = 5.603 μg mL^−1^, c_2_ = 7.844 μg mL^−1^, c_3_ = 11.206 μg mL^−1^ polymer representing N/P ratios of 5, 7, and 10 for PEI), all polyplexes were small with slightly positive zeta potentials. These results indicate optimal siRNA encapsulation already at low polymer excess with low to moderate polydispersities (Figure S38, Supporting Information). Solely, P(SPAA-co-DAA)3 was not able to fully encapsulate siRNA at a low concentration of c_1_; which equals an N/P ratio of 0.75 for this polymer. Incomplete encapsulation is concluded based on the negative zeta-potentials and large, polydisperse particles. Since more phosphate groups are present than nitrogen atoms at such a low N/P ratio, full condensations are rather uncommon. Additionally, spermine was not able to form polyplexes at all concentrations owing to its low molecular weight. Incompletely encapsulated siRNA on the surface of the polyplex causes negative zeta potentials as well as a detection of “free” siRNA as already shown for SYBR gold assays with spermine. Zeta-potentials of PEI-polyplexes were slightly positive to neutral at all concentrations even if those concentrations are comparably high for PEI in matters of N/P ratio (NP = 5 (c_1_), 7 (c_2_) and 10 (c_3_)). All other spermine-polymers showed similar or higher zeta-potentials, confirming that P(SpAA) were able to fully encapsulate siRNA already at low concentrations. No correlation between sizes and zeta potentials with increasing concentrations was found, as sizes remained small for all polyplexes with full siRNA encapsulation.

**Figure 2:**
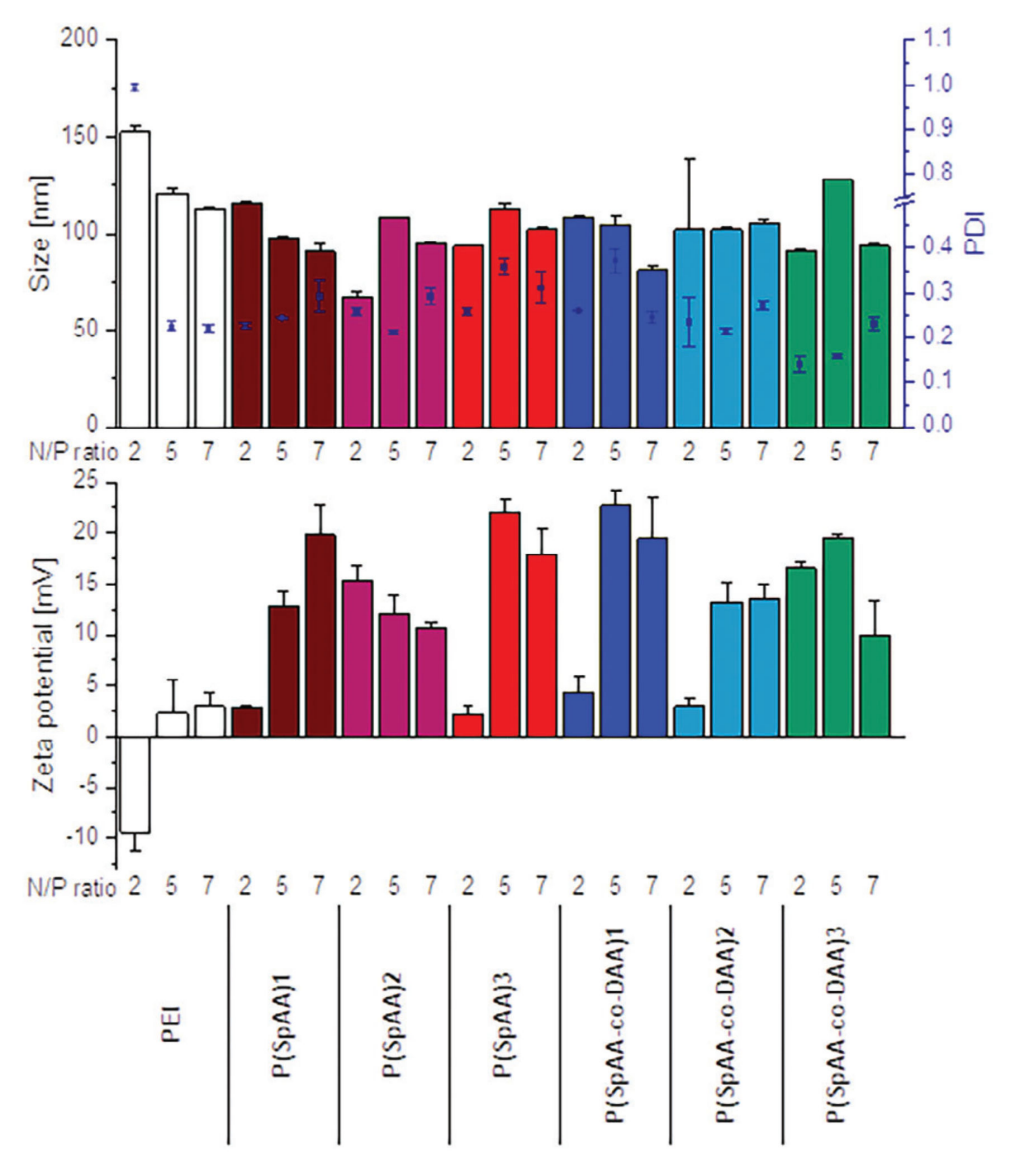
Dynamic light scattering and laser Doppler anemometry measurements of polyplexes formed with PEI, P(SpAA) 1−3 and P(SpAA-co-DAA) 1−3 (Top) Hydrodynamic diameters (left y-axis), polydispersity indices (PDI, right y-axis) and (B) zeta potentials of polyplexes at N/P ratios of 2, 5, and 7 (data points indicate mean ± SD, n = 3).

To further analyze sizes, polydispersities and zeta potentials with regard to the N/P ratio, measurements with all polymers were conducted at N/P ratios of 2, 5, and 7 that are relevant descriptors for in vitro experiments (Figure 2). Since PEI does not show full encapsulation of siRNA at N/P ratio of 2, zeta potentials for these polyplexes were negative and polyplexes showed increased sizes and polydispersities. Polyplexes from P(SpAA) displayed efficient siRNA encapsulation at low N/P ratios with small polyplexe sizes between 70 and 130 nm and positive zeta potentials (3 − 23 mV), indicating that these polymers can form optimal particles already at exceptionally low N/P ratios. Advantageously, the use of low polymer excess helps avoiding unwanted side or toxic effects due to a low number of amines present in the system. Polyplexes from P(SpAA)1, P(SpAA)3, P(SPAA-co-DAA)1, and P(SPAA-co-DAA)2 formed at N/P ratios of 2 showed very slightly positive zeta potentials indicating a low amount of excess positive charges. With increasing N/P ratios, zeta potentials became more positive while sizes remained ≈100 nm for these polymers. For all polyplexes, polydispersities were low to moderate with values raging between 0.13 and 0.37, with P(SPAA-co-DAA)3 forming the most monodisperse polyplexes (PDI = 0.14 − 0.23) at all tested N/P ratios, most likely due to its most amphiphilic character. Smaller^[91]^ as well as similar to larger sizes^[92]^ were reported in literature for amphiphilic PEI-polyplexes in comparison to the parent PEI indicating that the ability for a dense packaging of siRNA is individual for each cationic system. However, reported polyplex sizes for similar systems were mostly higher than our reported sizes of ≈100 nm.^[89,92,93]^

To evaluate polyplex stability in the presence of competing polyanions under neutral and acidic conditions, a heparin-modified SYBR gold competition assay was performed. The ability of P(SpAA)1-3 and P(SpAA-co-DAA)1-3 to protect siRNA in the presence of increasing concentrations of polyanionic heparin under physiologically relevant conditions of the cytoplasm (pH 7.4) was tested (Figure S40, Supporting Information, top). siRNA displacement at pH 7.4 from P(SpAA) polyplexes was not observed at low heparin concentrations and reached maximum release of less than 20% at high concentration of 1.00 I.U. heparin per well. P(SpAA)1 and P(SpAA)2 showed slightly better stability at pH 7.4 than P(SpAA)3 reflected by a higher concentration of heparin necessary to replace initial amounts of siRNA. In comparison, PEI formed more loosely assembled polyplexes as higher amounts of siRNA (up to 30%) were released generally, and siRNA replacement took place already at low concentrations of heparin. Similar observations were made for polyplexes with spermine at low heparin concentrations. At high concentrations of 1.00 I.U. heparin per well, a release of up to 80% siRNA was detected from unstable polyplexes formed with spermine that corroborates our earlier findings and investigations on low-molecular weight oligospermines.^[52]^ P(SpAA-co-DAA) copolymers showed less effective siRNA complexation at higher heparin concentration than homopolymers indicating that more hydrophobic sub-units lead to more loosely formed polyplexes. Polyplexes from P(SpAA-co-DAA)2 and 3 started to release siRNA at a concentration of 0.25 whereas P(SpAA-co-DAA)1 formed more stable polyplexes with concentrations of 0.50 I.U. of heparin necessary to displace small amounts of siRNA. At high heparin concentration, all polyplexes from copolymers release between 35 and 55% siRNA. Even if this release is higher than for the homopolymers and a low release would be preferred to confirm a high protection ability at pH 7.4, similar values were observed for other polycationic systems.^[52,70]^

Additionally, siRNA release ability under acidic conditions (pH 4.5), mimicking the endosomal compartment, was analyzed using a different buffer system (Figure S40, Supporting Information, bottom). siRNA was more easily released from P(SpAA) and PEI polyplexes reaching almost 100% release for P(SpAA)2 and 70−90% for P(SpAA)1, P(SpAA)3 and PEI due to higher number of protonated amines causing an elevated charge repulsion. A similar behavior was observed using copolymers, with all copolymers releasing 100% siRNA at high heparin concentrations, indicating suitable siRNA release abilities. Spermine, on the other hand, is not able to form any stable polyplexes at pH 4.5.

It seems that amphiphilic copolymers P(SpAA-co-DAA) 1− 3 and homopolymer 3 form less stable polyplexes that release siRNA even more efficiently than PEI. These polyplexes might be less stable due to lower charge density or higher steric hindrance caused by the hydrophobic subunits, which in turn also helps to release the payload at lower pH values in the endolysosomal compartment probably due to further destabilization caused by charge repulsion of additionally protonated amine groups.

### 2.5 In Vitro Performance of Polyplexes in Lung Cells

**Figure 3:**
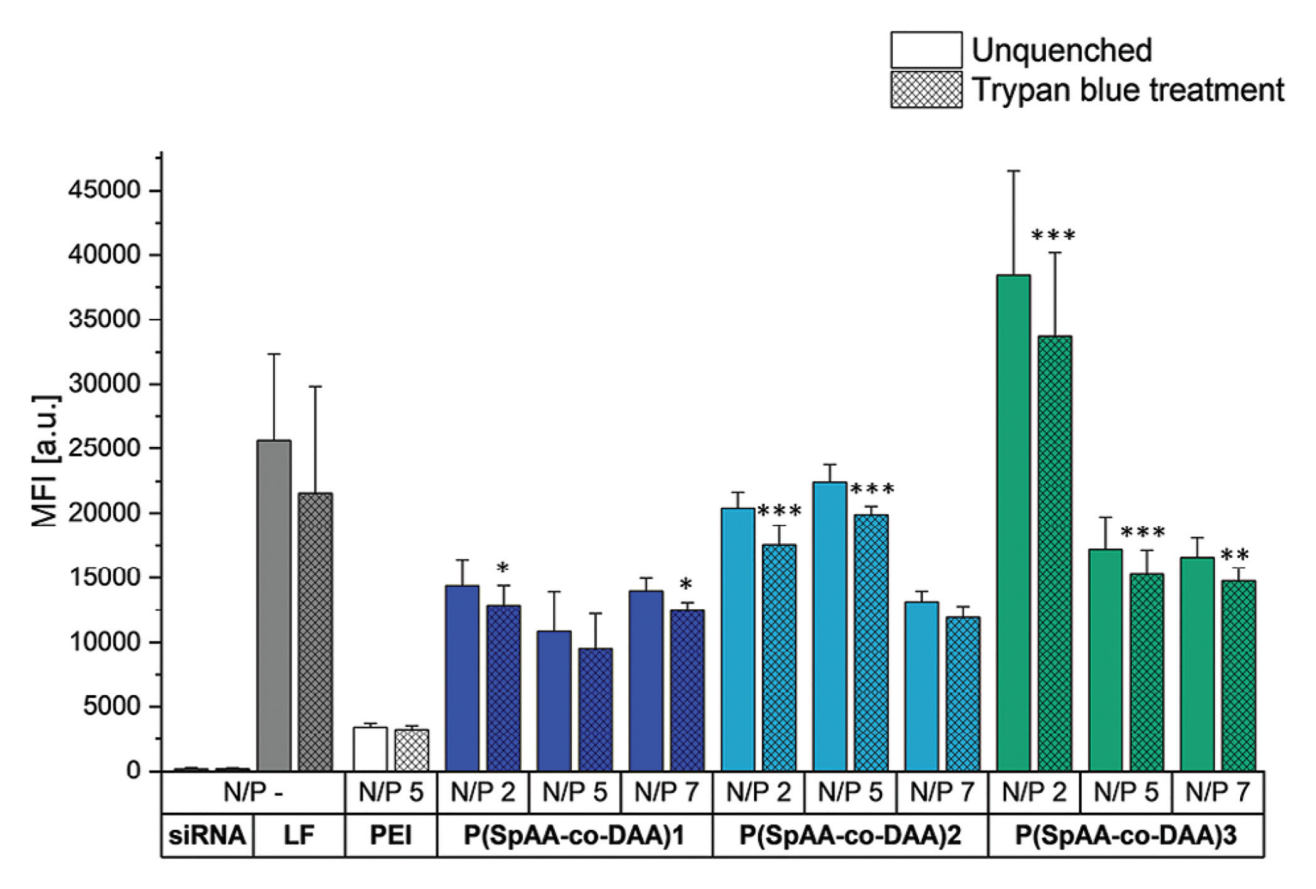
Cellular uptake of polyplexes made of AF488-labeled siRNA with PEI at N/P 5 or P(SpAA-co-DAA) 1−3 at N/P ratios of 2, 5, and 7 after 24 h of incubation, as quantified by flow cytometry performed with and without trypan quenching and presented as median fluorescence intensity. Cells treated with free siRNA served as negative control, whereas Lipo-fectamine (LF) lipoplexes served as positive control. Significance levels shown in comparison to PEI uptake.

To test the ability to mediate internalization into lung epithelial cells, cellular uptake of Alexa Fluor 488-labeled siRNA was quantified by flow cytometry determining the median fluorescence intensity (MFI) and polyplexes from P(SpAA) 1−3, P(SpAA-co-DAA)1-3 and PEI with fluorescent siRNA at N/P ratios of 2, 5, and 7 were analyzed. Trypan blue treatment, which was additionally applied in order to exclude extracellular fluorescent signals caused by cell surface-bound siRNA, resulted in insignificantly lower MFI values for all tested polyplexes, indicating that negligeable amounts of polyplexes were only attached to the outer cell membranes (Figure 3; Figure S41, Supporting Information).

PEI polyplexes showed similar uptake abilities independent from the used N/P ratios. For P(SpAA) 1−3 homopolymers, the fluorescence signals of cellularly internalized siRNA-AF488 decreased from N/P ratios of 2 to 7 (Figure S41, Supporting Information), which could be a result of densely-packed self-quenching of fluorescent siRNA at N/P 2.^[94]^ Independent of this observation, significantly higher uptake was reached at all N/P ratios of the three homopolymers in comparison to PEI-mediated siRNA uptake, highlighting that all P(SpAA) homopolymers are able to outperform commonly used PEI at all tested N/P ratios. Highest uptake was reached with P(SpAA)3 at N/P 2 that was about Six-times higher than observed in PEI-mediated up-take experiments. Similar trends were observed for copolymers P(SpAA-co-DAA)1-3 (Figure 3). All polyplexes induced higher cellular uptake in comparison to PEI-polyplexes with a correlation with the hydrophobic fraction of the polymer on the MFI, however without any clear influence of the N/P ratio.

Considering that the polyplexes made of the copolymers appeared slightly less stable in the heparin competition assay, the self-quenching effect of the fluorescent siRNA may be weaker resulting in similar intracellular fluorescence independent of the polymer/RNA ratio. P(SpAA-co-DAA)2 polyplexes formed at N/P 2 and 5 reached performance insignificantly different from positive control Lipofectamine. Remarkably, P(SpAA-co-DAA)3 polyplexes formed at N/P 2 were able to outperform “gold standard” Lipofectamine in our experiments and additionally induced 10-times higher cellular uptake of siRNA in comparison to PEI. To the best of our knowledge, none of the previously reported hydrophobically-modified PEI-polyplexes or spermine-containing polyplexes were able to outperform 25 kg/mol hyperbranched PEI by this far especially at such low N/P ratios of 2−7.^[91,95,96]^

To evaluate the gene silencing efficiency of spermine-acrylamide polyplexes on the protein level, H1299/eGFP cells were utilized that express the “enhanced green fluorescent protein” reporter gene (eGFP). Based on the most promising in vitro cellular uptake experiments, H1299/eGFP cells were transfected with freshly prepared or spray-dried P(SpAA-co-DAA)3 polyplexes formulated with siRNA against eGFP (siGFP) or with scrambled siRNA (siNC) as negative control using an N/P ratio of 5. Spray-dried particles with 5% trehalose were produced to obtain stable and inhalable dry powder formulations with long shelf life. However, spray drying can exert shear forces on nanoparticles and could disassemble the produced polyplexes. Therefore, DLS measurements were performed before and after spray drying to visualize any possible effects (Figure S39, Supporting Information). DLS of polyplexes formed with P(SpAA-co-DAA)3 and siGFP after resuspending subsequent to the spray-drying process only showed a slight increase in size and PDI. After 1 day of resuspension, sizes were small with lower polydispersities. Therefore, spray-dried particles can be used as dry-powder formulations that release polyplexes of desired characteristics. To additionally compare the in vitro efficiency of freshly prepared and spray-dried polyplexes made of P(SpAA-co-DAA)3 and siRNA, knockdown efficiencies were calculated (Figure S42, Supporting Information). Freshly prepared polyplexes showed a very high knockdown efficiency of 93%, while spray-dried polyplexes showed a similar efficiency of 96%, which are both exceptionally high results for an N/P ratio of 5 compared to other systems.^[58,97]^ Therefore, it was shown that the transfection ability and efficiency during spray drying is retained for in vitro experiments and that spray-dried formulations can be used in follow up studies in vivo.

Following validation of cellular uptake and transfection ability mediated by poly(spermine acrylamide)/siRNA polyplexes in vitro, further evaluation of uptake and knockdown abilities in a more in vivo relevant context was performed using air−liquid interface (ALI) cultures of lung epithelial cells since mucus is present in such an experiment.

Mucus visualization on Calu3 cells in ALI cultures was performed by WGA-AF488 staining of the mucus layer (Figure S44, Supporting Information). After 24 h of incubation time with polyplexes, AF647-siRNA can be found below the mucus layer, indicating successful polyplex transport across mucus.

**Figure 4:**
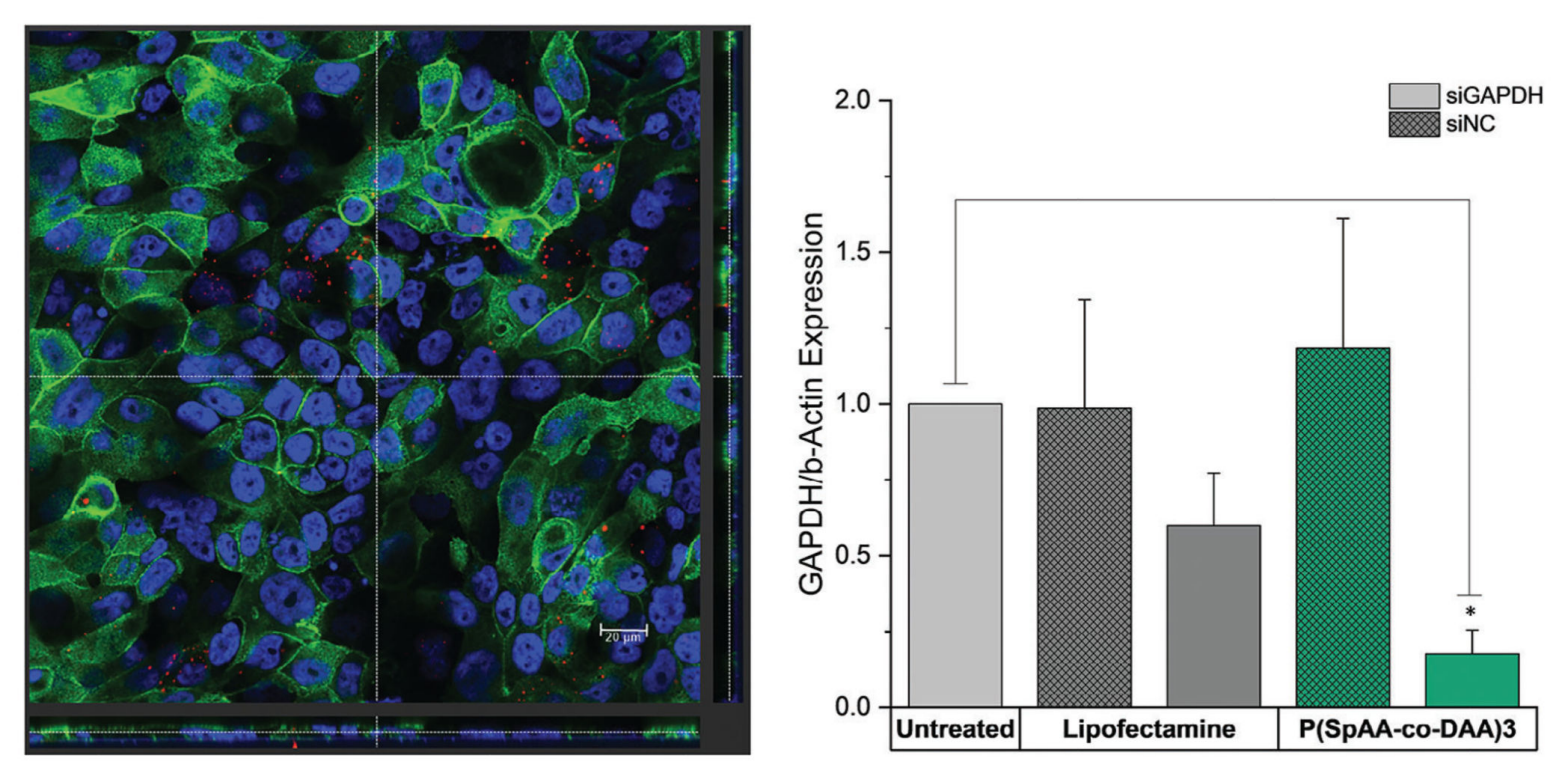
(Left) Orthogonal view of phalloidin staining of Calu3 cells at ALI. Incubation time: 24 h; Blue: DAPI (nuclei); Green: Rhodamine-labeled Phalloidin (cytoskeleton); Red: AF647-siRNA. (Right) GAPDH gene knockdown of P(SpAA-co-DAA)3 polyplexes using an N/P ratio of 5 in Calu3 cells grown in ALI cultures after 24 h transfection. 100 pmol hGAPDH siRNA were used. Blank samples consisted of Calu3 monolayers treated with 5% glucose only. The positive control consisted of Lipofectamine 2000 (LF) lipoplexes with 100 pmol hGAPDH siRNA. GAPDH expression was quantified by real time PCR and normalized to *β*-actin expression. Data points indicate mean ± SD (n = 3).

Cell uptake was visualized by staining of Calu3 cells in the presence of fluorescent siRNA (Figure 4, left; Figure S43, Supporting Information). Confocal microscopy confirmed that siRNA was located in the cytoplasm of Calu3 cells, emphasizing cellular uptake of polyplexes. The ability of spermine-acrylamides to silence an endogenously expressed gene in Calu3 monolayers, after successful mucus diffusion and cellular uptake, was additionally tested. Cells were transfected with P(SpAA-co-DAA)3 polyplexes containing siRNA against the housekeeping gene GAPDH (siGAPDH) or scrambled sequence siRNA (siNC as negative control). Negative controls consisted of blank cells that were treated with 5% glucose only, while positive control cells were transfected with Lipofectamine 2000. GAPDH gene expression was quantified by real time PCR and normalized to *β*-actin gene expression (Figure 4, right). Calu3 cells that were transfected with P(SpAA-co-DAA)3/siGAPDH polyplexes showed higher levels of gene knockdown when compared to the blank cells or those that were treated with P(SpAA-co-DAA)3/siNC polyplexes. Lower levels of reduced GAPDH gene expression were also observed in comparison to cells that were treated with LF/siGAPDH, indicating that P(SpAA-co-DAA)3 polyplexes are able to efficiently deliver siRNA to lung epithelial cells in an in vitro model of the lung, resulting in efficient gene knockdown. In models, which better mimic in vivo conditions,^[98]^ such as ALI cultures, and especially when mucus is present, lipofectamine seems to not be as effective as in standard in vitro experiments. This observation has been explained by pulmonary surfactants inhibiting lipofection in the alveolar space,^[99]^ and by interactions between lipophilic drugs or drug delivery systems with mucus, inhibiting permeation in mucosal tissues.^[100]^ On the other hand, polyplexes from P(SpAA-co-DAA)3 can still efficiently mediate target gene downregulation. Additionally, lipofectamine cannot be employed in in vivo experiments due to its toxicity. Just recently, we tested hyperbranched PEI and a highly efficient virus-inspired polymer for endosomal release (VIPER) in the same ALI-culture model, showing that both polymers induce less (PEI) or similar (VIPER) GAPDH knockdown as P(SpAA-co-DAA)3.^[101]^ Thus, P(SpAA-co-DAA)3 is a very promising candidate for safe and efficient siRNA delivery to lung cells via pulmonary delivery routes.

### 2.6 In Vivo Distribution of Polyplexes in Lung Cells

**Figure 5:**
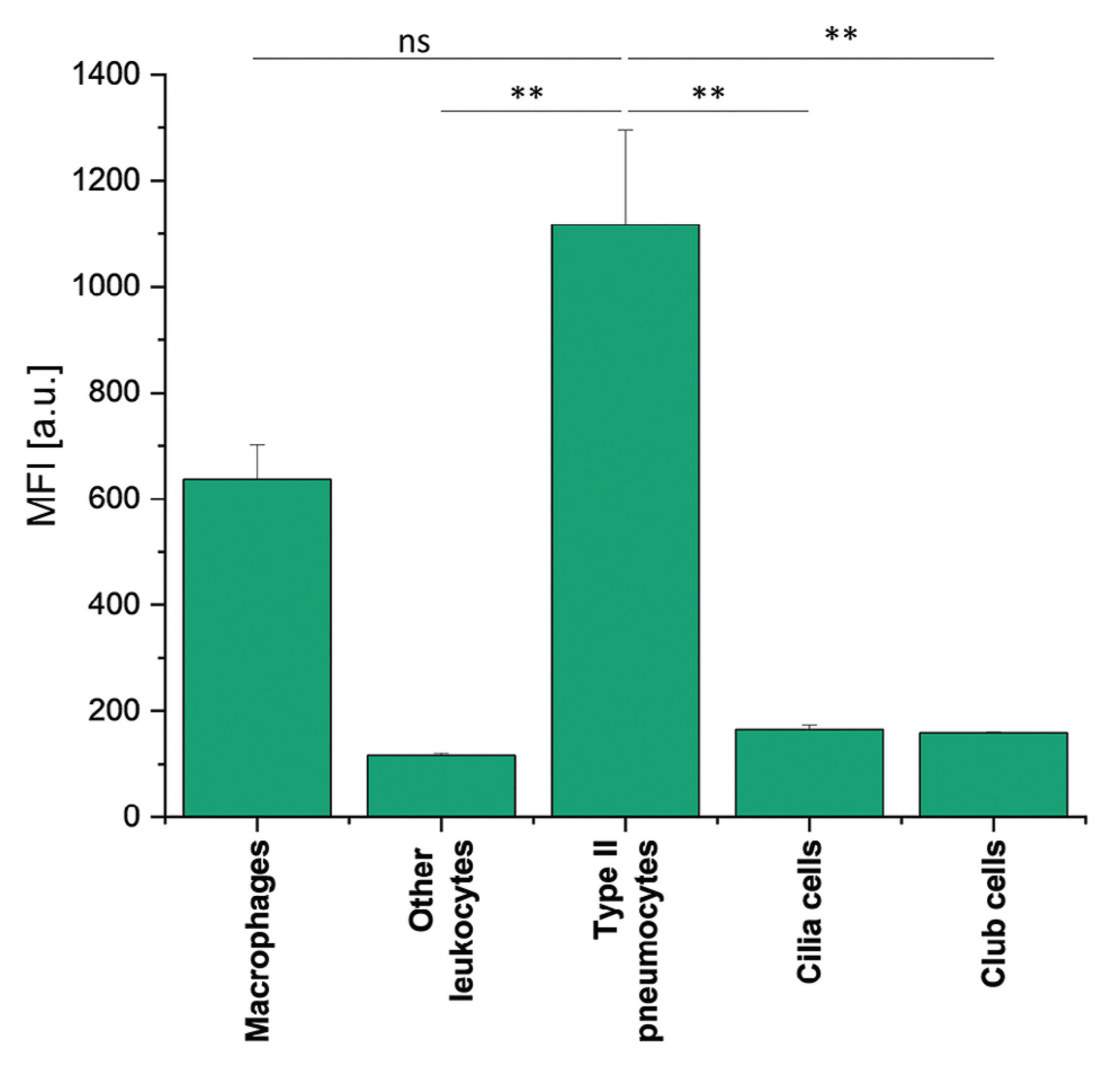
In vivo P(SpAA-co-DAA)3/siRNA polyplex distribution in lung cells using P(SpAA-co-DAA)3 at an N/P ratio of 5. Cellular uptake in different lung cell populations was quantified by flow cytometry. Data points indicate mean ± SEM.

To quantify the in vivo distribution of P(SpAA-co-DAA)3/siRNA polyplexes within the lungs and to determine uptake in different lung cell types, BALB/c mice were intratracheally administered with polyplexes loaded with AF647-labeled siRNA. After 48 h, bronchoalveolar lavage fluid (BALF) was collected while lungs were further processed to obtain single-cell suspensions. Different types of lung cells were counterstained with specific fluorescently labeled markers to quantify the fate of polyplexes after pulmonary administration. P(SpAA-co-DAA)3/siRNA polyplexes were mainly taken up by two cellular subsets: type II pneumocytes and macrophages confirming that siRNA can reach the site of action when being transported by spermine-polymers after intratracheal administration to lung (Figure 5). The cellular uptake observed in type II pneumocytes was higher (not significantly) than uptake in macrophages. Since Alveolar macrophages are the initial cellular defense in the deep lungs against foreign substances, uptake by this cell type is not surprising. Nevertheless, total macrophage clearance was avoided since small polyplexes of ≈100 nm were administered. Similar observations were recently made with PEI and VIPER/siRNA polyplexes.^[101]^

### 2.7 Gene Silencing and Distribution in Fibrotic Human Precision-Cut-Lung-Slices (hPCLS)

**Figure 6:**
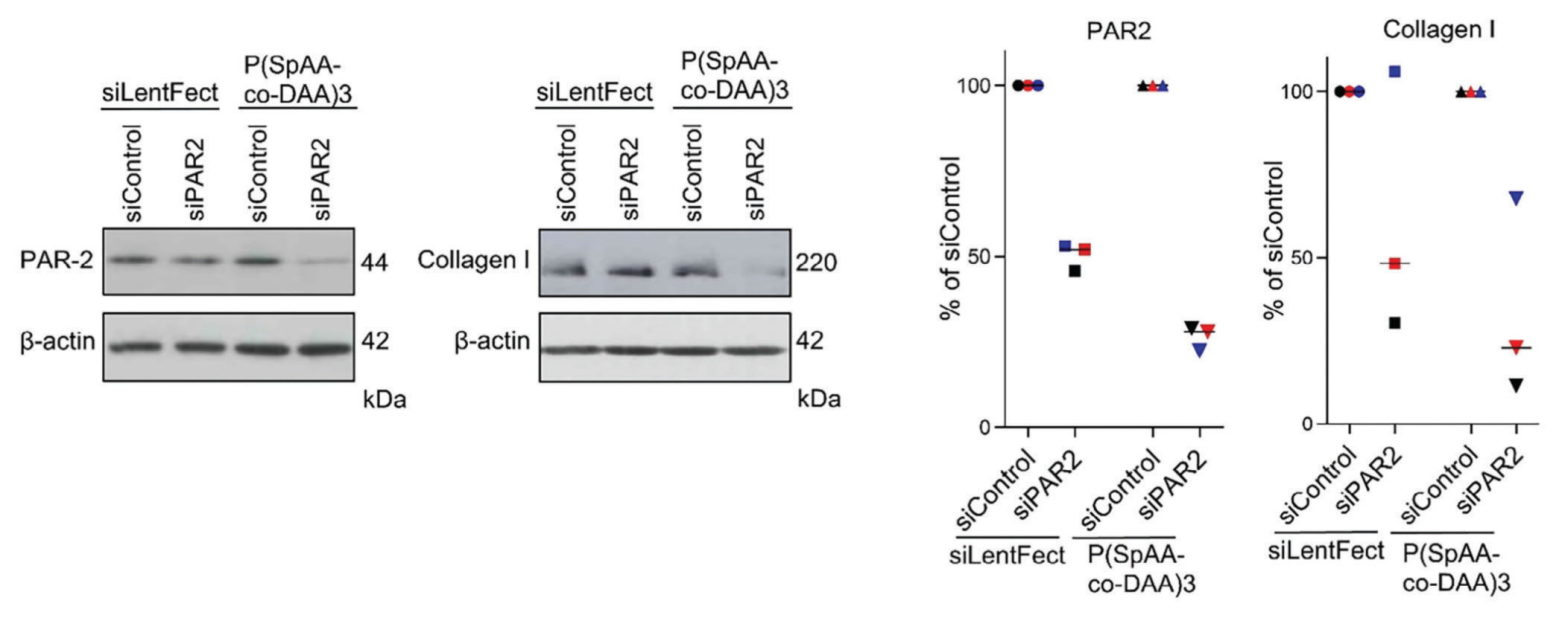
Ex vivo gene silencing efficacy of PAR2 and effects on collagen I levels in fibrotic human lung tissue from three donors transfected with P(SpAA-co-DAA)3/siRNA polyplexes at an N/P ratio of 5 in comparison to siLentFect. PCLS were transfected either with siRNA directed against PAR2 (siPAR2) or with siRNA control (siControl) and the proteins levels of PAR2 or collagen I were evaluated 72 h afterward. *β*-actin was used as a loading control. Western Blot analysis of one donor each is shown exemplarily in the left panels, and all results are summarized quantitatively by densitometric analysis in the right panels.

**Figure 7:**
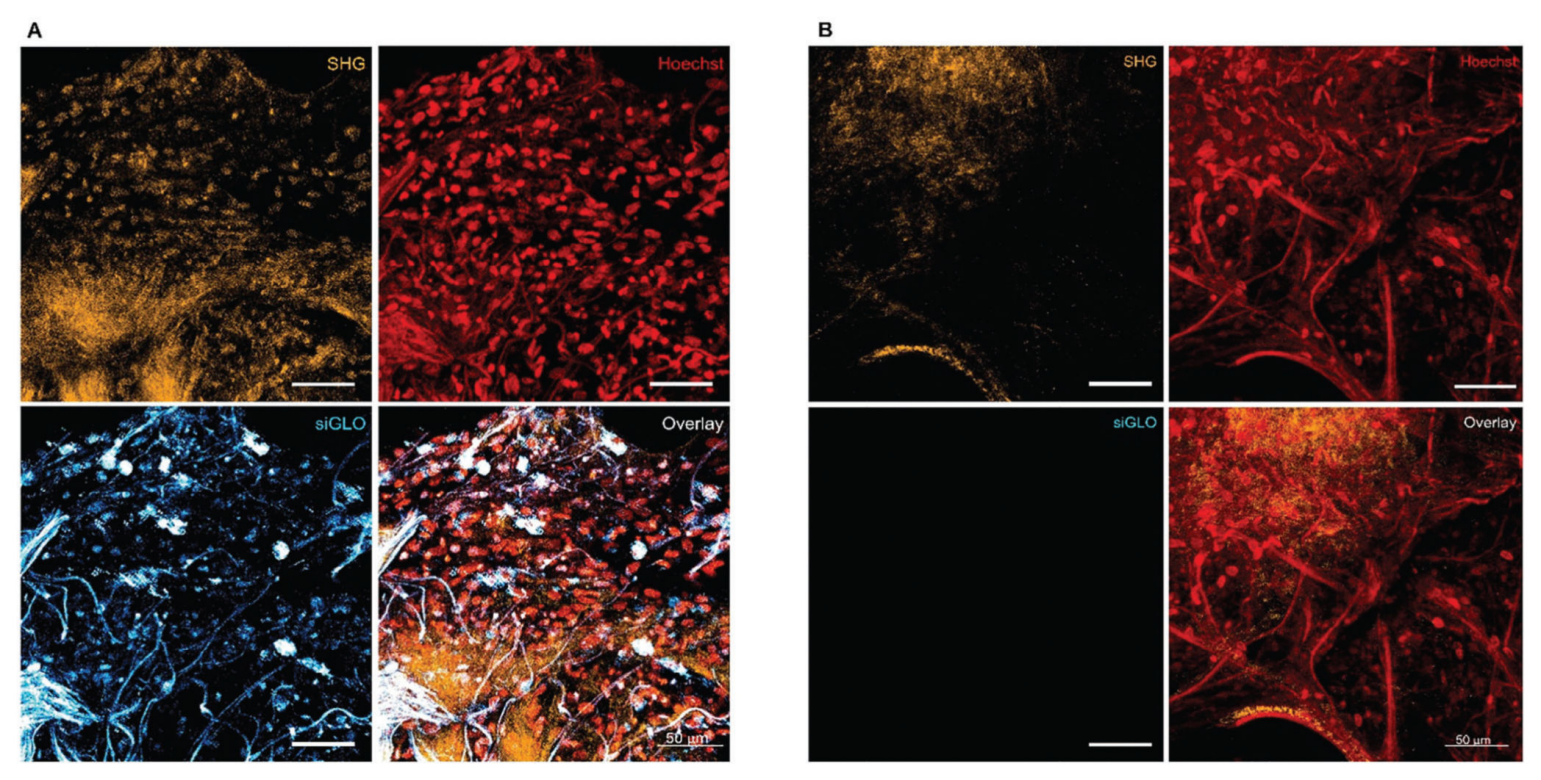
Two photon acquisition of ex vivo P(SpAA-co-DAA)3/siRNA polyplex distribution in fibrotic human lung tissue (hPCLS) transfected with siGLO transfection marker (in blue/cyan) at N/P 5 and marked with Hoechst (in red). The Second Harmonic Generation (SHG), which indicates the collagen type I and II fibers, is represented in orange. Panel A: transfection control with siGLO. Panel B: vehicle control without siGLO.

Epithelial cells are considered essential target cell types in a variety of pulmonary diseases, including lung fibrosis.^[31–33]^ Therefore, to further test the translational potential of this polymer-based siRNA delivery system, gene silencing efficacy in human fibrotic lung tissue was measured. As a model therapeutic target, the G protein-coupled receptor protease-activated receptor (PAR) 2 was chosen, which was previously silenced using siRNA in patient-derived lung fibroblasts.^[20]^ Accordingly, PAR2 knock-down was determined here in fibrotic human lung explants (hP-CLS) after transfection with P(SpAA-co-DAA)3 polyplexes and the downstream effect on collagen I reduction was assessed. For control purposes, lipoplexes made of siLentFect were used, as described before in patient material.^[20]^ In tissue samples from three donors, gene silencing of PAR2 was more prominent after transfection with the polyplexes compared with the lipoplexes in each donor tissue (Figure 6), confirming the results from the in vivo mimicking ALI model and emphasizing the tremendous potential of this polymer for therapeutic siRNA delivery. Moreover, collagen I levels were more efficiently reduced after transfection with the polyplexes, highlighting that siRNA delivery against PAR2 with our optimized polyplexes does indeed mediate anti-fibrotic therapeutic effects in patient tissue. The uneven collagen I levels between the three human donors have to be understood as a result of patient-to-patient variability and disease severity as well as anatomic explant origin. Two photon microscopy additionally underlined efficient intracellular delivery of fluorescently labeled siRNA, which can be found, particularly in the perinuclear regions. Despite typical autofluorescence in lung tissue, visualization of Cy3-labeled siGLO was not affected, as demonstrated by the vehicle control images (Figure 7).

### 2.8 Safety Assessment

MTT assays were used to colorimetrically evaluate cell proliferation as well as the viability of the cells treated with all spermine-containing homo- and copolymers and PEI. Mitochondrial activity in L929 mouse fibroblasts was assessed after 24 h of incubation using different polymer concentrations. Results are presented as the percentage of cell viability compared to untreated control cells. IC50 values were calculated by plotting cell viability and concentration of polymers on a logarithmic scale using a sigmoidal model fit (Figure S45, Supporting Information).^[102]^ All spermine polymers affected the cell viability less than PEI (25 kg mol^−1^, IC50 = 19.05 μg mL^−1^), and IC50 values for the spermine polymers were observed to be between 25 and 71 μg mL^−1^. P(SpAA-co-DAA)3 showed the lowest toxicity with regards to the IC50 value, most likely due to the lowest number of free amine groups when using the same polymer mass concentrations, which is in accordance with previous studies in which hydrophobically modified PEIs affected the cell viability less than the respective unmodified cationic motif.^[86,92,103]^ These results are very promising, especially considering that all spermine-polymers had much higher molecular weights than the tested PEI. In general, cytotoxicity correlates directly with molecular weight of polyamines.^[104]^ Therefore, lowering the molar mass of P(SpAA) homo and copolymers could have an additional beneficial effect on their biocompatibility.

To further test the influence of the newly developed polycationic polymers in a more relevant setup when used for pulmonary siRNA delivery, immune and cytokine responses toward polyplexes made of PEI or P(SpAA-co-DAA)3 were analyzed in mice. Bronchoalveolar lavage fluid (BALF) and BALF cells were collected after mice were intratracheally treated with PEI or P(SpAA-co-DAA)3/siRNA polyplexes, and levels of various cytokines were quantified in a multiplex-ELISA (Figure S46, Supporting Information).

For most of the tested cytokines, P(SpAA-co-DAA)3 treated mice showed similar or lower levels of inflammation than untreated mice (IL-23, IL-27, IFN-y, IL-12p70, IL-10, IFN-b). In some cases, the values were below the detection limit (IL-1a, GM-CSF).

Solely, IL-1b, IL-6, TNF-a and IL-17A levels were slightly, but not significantly higher after P(SpAA-co-DAA)3 polyplex treatment, and MCP-1 values showed a 6-fold increase. At the administered concentration, PEI/siRNA complexes showed similarly low proinflammatory effects, with an increased IL-23 level (not significant) and without increasing the secretion of MCP-1. In case of some cytokines, levels were lower after treatment with P(SpAA-co-DAA)3 as compared to PEI polyplexes. Taking into account that previous comparable in vivo inflammation studies of PEI for pulmonary delivery revealed that in vivo cytokine releases were generally higher with amphiphilic PEIs than with unmodified polymers,^[105,106]^ such generally low levels of cytokines of amphiphilic P(SpAA-co-DAA)3 indicate that spermine-polymers are a safe and biocompatible delivery system.

## 3 Conclusion

The research regarding carrier systems for pulmonary siRNA delivery has caught a lot of attention, since it offers a promising therapeutic approach by downregulation of disease-related gene expression via RNAi in lung-related diseases. In this study, the approach of using the endogenous, cationic molecule spermine as pendant groups in polyacrylamides was examined. A set of three different homopolymers and three copolymers with varying molecular weights and ratios of hydrophobic DAA-units were obtained. siRNA condensation ability and physicochemical characteristics, i.e., particle size, size distribution and zeta potential, were determined showing that all synthesized polymers were able to form uniform polyplexes with siRNA at favorable sizes of ≈100 nm in hydrodynamic diameter supplemented by narrow size distributions at very low N/P ratios even when spray-dried formulations were tested. These results in combination with slightly positive zeta-potentials and desired buffering-capacities indicate optimal conditions for efficient encapsulation and siRNA release. All homo- and copolymers showed an improved cellular uptake in lung cells. P(SpAA-co-DAA)3, with a ratio of 43/57 cationic to hydrophobic monomer subunits, mediated a better cellular uptake than lipofectamine and a ten-fold higher uptake than induced with PEI polyplexes under similar conditions. Efficient endosomal escape and siRNA release were verified additionally using protein knockdown in eGFP expressing cells and air−liquid interface studies. P(SpAA-co-DAA)3 induced a significant knockdown outperforming PEI and Lipofectamine in ALI-cultured Calu3 cells. In vivo studies, regarding biodistribution and safety in the lungs after intratracheal injection confirmed that P(SpAA-co-DAA)3 treated mice showed similar or lower levels of most tested cytokines than untreated mice and that P(SpAA-co-DAA)3 polyplexes efficiently reached type II pneumocytes, club and ciliated cells in the lung. Gene silencing in fibrotic human lung explants exemplified the superiority of the developed spermine-polymers compared to commercially available transfection reagents on the target protein level but also with regards to therapeutic downstream effects on collagen I levels. These findings qualify these spermine based homo- and copolymers as pulmonary nucleic acid carriers for therapeutic applications in lung diseases such as lung fibrosis, respiratory viral infections, asthma, or COPD.

## 4 Experimental Section

### Materials and Methods for Polymer Synthesis and Characterization of Polymer-Samples

All reactions were carried out under nitrogen atmosphere using standard Schlenk techniques. All glassware was heat dried under vacuum prior to use. Unless otherwise stated, all chemicals were purchased from Sigma−Aldrich, Acros Organics, or TCI and used as received. Hyperbranched polyethylenimine (PEI, 25 kg mol^−1^) was obtained from BASF (Ludwigshafen, DE). D(+)-Trehalose dihydrate was acquired from VWR International GmbH (Darmstadt, Germany). Solvents for polymerization were used in an anhydrous form unless otherwise stated.

NMR spectra were recorded on a Bruker AVIII-300, AVIII-400, an Avance III HD Bruker BioSpin 400 or AVIII-500 Cryo spectrometer at Ludwig-Maximilians University Munich or Technical University of Munich in the respective NMR facilities.

Unless otherwise stated, ^1^H- and ^13^C-NMR spectroscopic chemical shifts *δ* are reported in ppm. *δ* (^1^H) is calibrated to the residual proton signal, *δ* (^13^C) to the carbon signal of the solvent. Deuterated solvents were obtained from Sigma−Aldrich, Deutero or Eurisotop.

Elemental analysis was measured at the Laboratory for Microanalysis of the Central Analytics of the Faculty of Chemistry and Pharmacy, Ludwig-Maximilians-University Munich, on an Elementar vario EL or Elementar vario micro cube.

ESI-MS analysis was performed using a Thermo Finnigan LTQ FT Ultra Fourier Transform ion cyclotron resonance mass spectrometer in positive ionization mode. The resolution was set to 100 000 at 400 m/z. Depending on the respective measurement, mass ranges from 50 to 2000 u were detected. The spray capillary voltage on the IonMax ESI head was 4 kV, the heater capillary temperature 250 °C, the nitrogen sheath gas flow 20, and the sweep gas flow 5 units.

Molecular weights and polydispersities of polymers were measured via size exclusion chromatography (SEC) with samples of 3−5 mg mL^−1^ concentration either on a Varian 390-LC at 30 °C equipped with two PL Gel 5 μm Mixed C columns using a refractive index detector (RI detector) with chloroform as eluent relative to polystyrene standards or on an Agilent PL-GPC 50 equipped with two PL-Polargel-M columns and an RI detector at 30 °C using a mixture of DMF and 0.025 mol L^−1^ lithium bromide as eluent relative to poly(methyl methacrylate) (PMMA) depending on the solubility of the polymers.

### General Homopolymerization Procedures

After dissolving the calculated amount of monomer in toluene (monomer concentrations of 10 wt./vol.%), the respective equivalents of AIBN (2−10 wt%) were added. The solution was purged with nitrogen for 20 min and then immersed in an oil bath at 65 °C. At the end of the reaction time, polymerization was stopped by cooling the reaction mixture in an ice bath. The polymers were either washed with toluene if they already precipitated during reaction or were isolated by addition of the reaction mixture to a solvent (hexane or acetonitrile), centrifugation and then the solution was decanted off, if necessary, followed by a washing step. Afterward, the sample was dried in a vacuum oven at 50 °C. The structure and purity of the polymers were analyzed via ^1^H NMR spectroscopy and SEC analysis in DMF or chloroform.

*General Copolymerization Procedures*: After dissolving a calculated amount of NAS and DAA in toluene to obtain a concentration of 10 wt./vol.%, 10 wt% of AIBN were added. The solution was purged with nitrogen for 20 min and then immersed in an oil bath at 65 °C. At the end of the reaction time, polymerization was stopped by cooling the reaction mixture in an ice bath. The polymers were either washed with toluene if they already precipitated during reaction (P(NAS-*co*-DAA)1, P(NAS-*co*-DAA)2) or precipitated and washed by addition of the reaction mixture to hexane ((P(NAS-*co*-DAA)3) followed by centrifugation and an additional washing step. The solvent was decanted off, and the sample was dried in a vacuum oven at 50 °C. The structure, purity, and ratio of the two monomer units were analyzed via ^1^H NMR spectroscopy. In case of P(NAS-*co*-DAA)1 and P(NAS-*co*-DAA)2 deuterated DMSO was used as solvent due to insolubility of the NAS subunit in deuterated chloroform. The ratio of NAS/DAA was calculated using the methyl group signal of DAA (*δ* = 0.8 ppm) and the signal at 2.8 ppm assigned to four protons of NAS (see ^1^H NMR spectra of P(NAS) homopolymers). NMR spectra for P(NAS-*co*-DAA)3 were measured in CDCl_3_ and the ratio of NAS/DAA was calculated using the methyl group signal of DAA (*δ* = 0.8 ppm) and the signal at 2.8 ppm assigned to 4 protons of NAS (see ^1^H NMR spectra of P(NAS) homopolymers). The number average molecular weight (*M*_n_) and molecular weight distribution (Ð) were determined by SEC analysis in DMF relative to PMMA standards.

### General Post-Polymerization Procedures

*Synthesis of P(Tri-Boc Spermine Acrylamide)*: To a solution of one equivalent of the obtained P(NAS) in DMF (4 mL) at 40 to 60 °C, one equivalent of tri-Boc spermine dissolved in DMF (2 mL) was added dropwise and stirred overnight. The solvent was removed in vacuo, then chloroform (20 mL) and concentrated NH_3_ (aq.) (20 mL) were added to the residue and the reaction mixture was stirred for 2 h at room temperature. The separation of the phases was performed via centrifugation and the aqueous phase was decanted off. The organic phase was washed with water (1 × 40 mL) and brine (1 × 40 mL), dried over magnesium sulfate, and filtered. The solvent was removed in vacuo until ≈4 mL of the reaction mixture were left. The polymers were precipitated by the addition of the reaction mixture to hexane, followed by centrifugation. The solvent was decanted off, if necessary, followed by a washing step. The product was further dried in a vacuum oven at 50 °C and if necessary, residual solvent was removed by freeze-drying from benzene overnight. The structure and purity of the polymers were analyzed via ^1^H NMR spectroscopy. The number average molecular weight (*M*_n_) and molecular weight distribution (Ð) were determined by SEC in DMF.

### Synthesis of P(TBSpAA-co-DAA)

To a solution of the obtained P(NAS-*co*-DAA) in DMF (5 mL) at 40 °C, Tri-boc spermine (one equivalent with regard to the NAS repeating unit) dissolved in DMF (4 mL) was added dropwise and the reaction mixture was stirred overnight. The solvent was removed in vacuo, then chloroform (20 mL) and concentrated NH_3_ (aq.) (20 mL) were added to the residue and the reaction mixture was stirred for 2 h at room temperature. The separation of the phases was performed via centrifugation and the aqueous phase was decanted off. The organic phase was washed with water (1 × 40 mL) and brine (1 × 40 mL), dried over magnesium sulfate, and filtered. The solvent was removed in vacuo until ≈4 mL of solvent were left. The polymers were isolated by the addition of the reaction mixture to hexane, centrifugation and then the solvent was decanted off. The product was dried in a vacuum oven at 50 °C and if necessary, residual solvent was removed by freeze-drying from benzene overnight. The structure, purity and subunit ratio were analyzed via ^1^H NMR spectroscopy in CDCl_3_. The ratio of TBSpAA/DAA was calculated using the methyl group signal of DAA (*δ* = 0.8 ppm) and the broad signal at 3.0 ppm consisting of two protons of DAA and 12 protons of TBSpAA (see ^1^H NMR spectra of P(TBSpAA) homopolymers). Number average molecular weights (*M*_n_) and molecular weight distributions (Ð) were determined by SEC in DMF relative to PMMA standards.

### General Deprotection Procedures

*Deprotection of P(Tri-Boc Spermine Acrylamide)*: Trifluoro acetic acid (2 mL) was added at room temperature to the respective Boc-protected polymer in one portion. The reaction mixture was stirred for 2 h at room temperature. The polymers were isolated by the addition of the reaction mixture to hexane or diethyl ether, centrifugation, and then the solution was decanted off, if necessary, followed by a washing step. Subsequently, the polymers were dissolved in water, filtered (1 μm PES-syringe filter), and freeze-dried from water. The structure and purity of the TFA-salts of the polymers were analyzed via ^1^H NMR spectroscopy in D_2_O.

### Deprotection of P(TBSpAA-Co-DAA)

TFA (2 mL) was added to the obtained P(TBSpAA-*co*-DAA) polymers at room temperature. The reaction mixture was stirred for 2 h at room temperature. The polymers were precipitated by addition of the reaction mixture to diethyl ether followed by centrifugation. The solvent was decanted off and the polymers were dissolved in water and filtered (1 μm PES syringe filter). After freeze-drying from water, the structure and purity and ratio of SpAA/DAA were calculated via ^1^H NMR spectroscopy in D_2_O of the TFA salts of the polymers. The ratio of SpAA/DAA was calculated using the methyl group signal of DAA (*δ* = 0.8 ppm) and the broad signal at 3.0 ppm consisting of two protons of DAA and 12 protons of SpAA (see ^1^H NMR spectra of P(SpAA) homopolymers).

### Monomer Reactivity Ratios of NAS and DAA

After dissolving varying ratios of NAS and DAA (10:90 → 90:10; overall 0.594 mmol) in toluene (0.5 mL), AIBN (0.2 m in toluene; 17.8 μL, 0.6 mol%) was added. Each reaction mixture was stirred for 2 min at 60 °C, then the polymerization was stopped by adding hydroquinone as a radical scavenger. The conversion of both monomers was determined via ^1^H NMR spectroscopy in CDCl_3_. If conversion was below 10%, polymerization time, initial feed monomer composition, and final copolymer composition were used for determination of reactivity ratios by Fineman−Ross technique.^[76]^

### Buffering Capacity

Acid−base titration studies over pH values ranging from 12 to 2 were performed to determine the buffering capacity of the studied polymers. In brief, an aqueous polymer solution with a concentration of 1 or 5 mg mL^−1^ was prepared and the pH was adjusted to 12 to 11.5 with 0.1 m NaOH. Subsequently, the solution was titrated with 0.1 m HCl and pH change was measured after every 50 μL addition, until the pH of the polymer solutions decreased to a constant pH of 2−3. The pH value was monitored with a pH-meter and an electrode at 25 °C (Fisherbrand accumet AB150, Fisher Scientific GmbH, Schwerte, Germany).

### Critical Aggregation Concentration (CAC)

The measurement of the CAC of the studied polymers was carried out according to literature.^[82]^ A Nile red stock solution was prepared by dissolving the dye in DMSO at a concentration of 0.794 mg mL^−1^. Multiple stock solutions of each polymer were prepared in high-purity water, then combined with the Nile red stock solution, and additional water high purity water was added to provide the final concentrations to obtain a concentration series of each polymer. The solutions were sonicated for 30 min at 35 °C and incubated at 25 °C for 5 h. After incubation, the solutions were transferred to a black 96-well microplate (Microplate, PS 96 well, Black, Fluotrac, Greiner Bio-One, Frickenhausen, Germany). Excitation/emission wavelengths of 485/636 nm were used measured on a microplate reader (TECAN Spark, TECAN, Maennedorf, Switzerland).

### Materials and Methods for Cell Culture

Highly purified water (HPW) was provided by Ludwig−Maximilians-University Munich. HEPES (4-(2-hydroxyethyl)−1-piperazineethanesulfonic acid), sodium acetate, Heparin sodium salt, Thiazolyl blue tetrazolium bromide (MTT), RPMI-1640 Medium, Fetal Bovine Serum (FBS), Penicillin-Streptomycin solution, Dulbecco’s Phosphate Buffered Saline (PBS), Trypsin-EDTA solution 0.05%, L-glutamine solution 200 mM, dimethyl sulfoxide (DMSO) and Geneticin (G418) disulfate solution were purchased from Sigma−Aldrich (St. Louis, MO, USA). NCI-H1299 (human non-small cell lung carcinoma) cell line, green fluorescent protein (GFP) reporter cell line-NCI-H1299 (human non-small cell lung carcinoma), and L929 cells (subcutaneous tissue fibrob-lasts) were purchased from ATCC (Manassas, VA, USA). SYBR Gold Dye, Lipofectamine 2000 Transfection Reagent, AlexaFluor 488 (AF488) dye were purchased from Life Technologies (Carlsbad, California, USA). HyClone trypan blue solution 0.4% in phosphate buffered saline was obtained from FisherScientific (Hampton, New Hampshire, USA). Amine modified GFP siRNA (5′ − pACCCUGAAGUUCAUCUGCACCACcg, 3′ − ACUGGGACUUCAAGUAGACGGGUGGC) and scrambled siRNA (5′ − pCGUUAAUCGCGUAUAAUACGCGUat, 3′ − CAGCAAUUAGCGCAUAU-UAUGCGCAUAp) were purchased from Integrated DNA Technologies (Coralville, IA, USA) (indication of modified nucleotides: “p” denotes a phosphate residue, lower case letters are 2′-deoxyribonucleotides, capital letters are ribonucleotides, and underlined capital letters are 2′-O-methylribonucleotides).

NCI-H1299 cells (human non-small cell lung carcinoma cells) and L929 cells (mouse subcutaneous tissue fibroblasts) were cultured in RPMI-1640 media supplemented with heat inactivated FBS (10%) and Penicillin-Streptomycin (1%). GFP reporter cell line-NCI-H1299 was cultivated in RPMI-1640 media supplemented with heat inactivated FBS (10%), Penicillin−Streptomycin (1%) and 0.4% (v/v) Geneticin (G418). The plasmid for GFP expression contains an antibiotic resistance for Geneticin to enable the selection of stably expressing GFP cells. All cells were subcultured, maintained, and grown in an incubator under humidified air with 5% CO_2_ at 37 °C.

### Preparation of Polyplexes

To prepare polymer-siRNA complexes (polyplexes), aqueous polymer stock solutions (1 mg mL^−1^) were diluted with freshly, sterile filtered HEPES buffer (pH 7.4) to predetermined concentrations. Afterward, the polymer solution was added to a defined amount of siRNA in a microcentrifuge tube and vigorously mixed to obtain polyplexes at various N/P ratios. An incubation time of 1 h facilitated a stable polyplex formation. The N/P ratio is defined as the molar ratio between the polymers’ amine groups (N) and the siRNA’s phosphate groups (P). The amount of polymer needed to obtain different N/P ratios was calculated according to following equation:

$$\begin{array}{c} \text{m}(\;\text{polymer}\;\text{in}\;\text{pg})=\text{n}\;\text{siRNA}\;(\text{pmol})\times \;\text{protonable}\;\text{unit}\;(\text{g}/\text{mol})\\ \times \text{N}/\text{P}\times \text{number}\;\text{of}\;\text{nucleotides}\;\text{siRNA}\; \end{array}$$

The number of nucleotides of 25/27mer siRNA is set to 52.

Lipofectamine polyplexes were prepared according to the manufacturer’s protocol.

### Size and Zeta (ζ)-Potential Analysis by Dynamic Light Scattering and Laser Doppler Anemometry

Particle size, polydispersity index (PDI), and zeta potential of polyplexes were measured using a Zetasizer Nano ZS (Malvern Instruments, Malvern, UK). After 1 h incubation time, 100 μL of freshly prepare’d polyplexes were used for particle size and PDI measurements. Zeta potentials were measured using a Zeta Cell (Zetasizer Nano series, Malvern, UK) containing a 6.5X dilution of the same 100 μL sample of polyplex suspension. Results are expressed as mean ± standard deviation (n = 3; the diameter was averaged over three independent values consisting each of 15 measurements). For redispersability of spray dried formulations, ≈3.5 mg od the spray-dried polyplex formulations were dissolved in 70 μL HPW and measured as described above.

### Spray Drying of Polyplexes

For microparticle preparation a B-290 (Büchi Labortechnik, Essen, Germany) was used. Spray-drying was performed according to literature.^[97]^ Spray-dried polyplexes were prepared in 5% w/v trehalose.

### siRNA Encapsulation Measured by SYBR Gold Assay

SYBR Gold assay was used to evaluate the capacity of the polymers to condense siRNA at various N/P ratios as previously described.^[52]^ For each batch, 100 μL of polyplex solution was transferred to a black FLUOTRAC 96 well plate (Greiner Bio-One, Frickenhausen, Germany). A 4X SYBR Gold solution (30 μL) was added to each well and the plate was incubated for 10 min in the dark. The fluorescence signal was determined using a fluorescence plate reader (TECAN Spark, TECAN, Maennedorf, Switzerland) at 485 and 535 nm excitation and emission wavelengths. Analogue procedure with free siRNA was used as 100% value. Measurements were performed in triplicates and results are shown as mean values ± standard deviation (n = 3).

### Heparin Assay

Heparin assays were used to determine the stability of the polyplexes at a physiologically relevant pH 7.4, as well as at pH 4.5 representing the late endosome. N/P ratio of 7 was chosen for all polymer-siRNA polyplex formations for testing the stability against increasing amounts of heparin (0, 0.1, 0.25, 0.5, 0.75, and 1.0 international Unit (1 IU = 4.926 μg heparin sodium salt). All polyplexes were formed as described in the preparation of polyplexes. The samples at pH 7.4 were prepared in 100 μL 10 mm HEPES buffer, while the samples measured at pH 4.5 were prepared in 100 μL sodium acetate buffer. Samples were prepared as described for the SYBR gold assay with the exception of adding 10 μL of heparin solution at various concentrations (0 − 1.0 IU per well) and incubated for another 30 minutes before adding the SYBR Gold solution. The fluorescence was measured at 485 and 535 nm excitation and emission wavelengths using a microplate reader (TECAN Spark, TECAN, Maennedorf, Switzerland). The experiments were run in triplicates and results were analyzed as described before for the SYBR Gold assay.

### MTT Assay

Cytotoxicity of free polymers (PEI, P(SpAA)1-3 and P(SpAA-*co*-DAA)1-3) was tested via an MTT Assay as described previously.^[107,108]^ 5000 L929 cells per well were seeded in a transparent 96 well plate (BioLite 96 well multidish, Thermo Fisher Scientific, Rochester, New York, USA). After 24 h, different stock concentrations of free polymers were diluted in 10 mm HEPES buffer (pH 7.4) to a final volume of 10 μL. This volume was added to 90 μL of prewarmed RPMI-1640 medium to obtain polymer concentrations of 5, 10, 15, 25, 50, and 100 μg mL^−1^. The cell medium of every well was aspirated and 100 μL of polymer containing media was added to each well and incubated for 24 h at 37 °C and 5% CO_2_. As a positive control, cells were incubated in 100 μL consisting of 10 μL 10 mm HEPES buffer (pH 7.4) and 90 μL medium. After 24 h, media was aspirated and 100 μL of MTT containing medium (0.5 mg mL^−1^ in serum-free RPMI-1640 medium) was added to each well. Cells were incubated for another 3 h in the incubator. Subsequently, the cell culture medium was completely removed and insoluble purple formazan crystals, converted from water-soluble MTT by metabolically active mitochondria,^[109]^ was dissolved in 200 μL DMSO. The absorption was measured at 570 nm, corrected with background values measured at 680 nm, using a microplate reader (TECAN Spark, TECAN, Männedorf, Switzerland). Data is shown as mean ± SD from three independent experiments that were run in duplicates as percentage of viable cells in comparison to untreated cells representing 100% viability. IC 50 values were calculated by plotting the concentration on a logarithmic axis against cell viability using OriginPro 2019b software (OriginLab, Northampton, Massachusetts, USA).

### Quantification of Cellular Uptake by Flow Cytometry

Flow cytometry was used to quantify the in vitro cellular uptake of polyplexes. Amine modified siRNA was labeled with the fluorescence dye Alexa Fluor 488 (siAF488) following the manufacturer’s protocol and purified by ethanol precipitation and spin column binding as described previously.^[110]^ NCI-H1299 cells were seeded in 24 well plates at a density of 50 000 cells per well and incubated for 24 h at 37 °C and 5% CO_2_. For all uptake experiments, negative controls consisted of cells treated with free siRNA (siAF488), while positive control cells were transfected with Lipofectamine 2000-siAF488 lipoplexes, which were prepared according to the manufacturer’s protocol. Cells were transfected with positive and negative controls and polyplexes containing 100 pmol siAF488 for 24 h. After incubation, medium was aspirated, cells were washed with prewarmed PBS and detached using 0.05% trypsin-EDTA. RPMI-1640 medium was added to all samples and spun down at 400G for 5 min using a centrifuge (Heraeus Megafuge 16R, Thermo Fisher Scientific, Osterode am Harz, Germany). Medium was aspirated without taking out the cell pellet. Samples were washed two times with PBS and resuspended in 500 μL PBS/2 mm EDTA. Median fluorescence intensities (MFI) were analyzed using an Attune NxT Acoustic Focusing Cytometer (Thermo Fisher Scientific, Waltham, Massachusetts, USA) by exciting the siRNA-AF488 at 488 nm and measuring the fluorescence signal with an 530/30 nm emission filter. Subsequently, trypan blue quenching was used to exclude surface fluorescence signals of not completely internalized siRNA-complexes and MFI of samples was measured again. Data is shown from two independent experiments that were run in duplicates, each sample consisting of a minimum of 10 000 viable cells. Three-way ANOVA with Bonferroni mean comparison (p > 0.05 considered not significant (ns); *p <0.05, **p <0.01, ***p <0.001 considered significantly different) was performed with OriginPro 2019b software (OriginLab, Northampton, Massachusetts, USA).

### In vitro eGFP Knockdown

To determine, if polyplexes can efficiently knockdown protein levels in cells, the green fluorescent reporter gene (eGFP) was chosen to be silenced by siRNA (siGFP) comprised polyplexes. 25 000 NCI-H1299/eGFP cells per well were seeded in 24 well plates in 500 μL RPMI-1640 + 0.4% G418 medium and grown for 24 h at 37 °C in humidified atmosphere with 5% CO_2_. As negative control polyplexes containing 50 pmol of scrambled siRNA (siNC) were used. Nanoparticle formation was followed as described for the preparation of polyplexes. Spraydried formulations were resuspended in high-purity water to reach a 5% trehalose solution. Cells were transfected with siGFP-polyplexes for 48 h at 37 °C in humidified atmosphere with 5% CO_2_. Subsequently, cells were trypsinized and prepared for flow cytometry measurements as described for cellular uptake experiments. MFIs of samples were quantified using an Attune Cytometer (Thermo Fisher Scientific, Waltham, Massachusetts, USA) with an 488 nm excitation laser and an 530/30 nm emission filter. Data is shown from two experiments, each sample consisting of a minimum of 10 000 viable cells.

### Methods for Air−Liquid Interface (ALI) Cell Culture

*Materials*: Eagle’s Minimum Essential Medium (EMEM), Trypsin 0.25%, PBS, Tween 20, and formaldehyde solution were purchased from Sigma−Aldrich (St. Louis, MO, USA). Rhodamine phalloidin, 4′,6-diamidino-2-phenylindole (DAPI), AF488-wheat germ agglutinin were obtained from Life technologies. FluorSave was purchased from Merck Millipore. Transwell polyester cell culture inserts (6.5 mm, 0.4 μm pore) were purchased by Corning. PneumaCult ALI medium was obtained by STEMcell technology.

### Culturing Conditions

Calu3 cells were maintained in EMEM medium supplemented with 10% FBS and 1% P/S. Cells were cultivated in 75 cm^2^ and split with 0.25% trypsin/EDTA solution. Cells were maintained at 37 °C and 5% CO_2_.

Calu3 cells were seeded at a density of 250 000 cells onto uncoated Tran-swell polyester cell culture inserts (6.5 mm, 0.4 μm pore, Corning, USA) in 100 μL of medium, while 700 μL were added to the basolateral chamber. After 72 h, apical medium was removed from the inserts to obtain ALI conditions, while the basolateral medium was replaced with 200 μL of PneumaCult ALI medium (STEMcell technology, Vancouver, Canada) and replaced every two days. Experiments were performed after TEER values ≥ 300 Ω*cm^2^ were reached as measured by EVOM epithelial volt/ohm meter (World Precision Instruments, Sarasota, USA).

### Phalloidin staining

For microscopy experiments, amine modified siRNA was labeled with succinimidyl ester (NHS) modified AlexaFluor647 (Life Technologies, Carlsbad, USA) according to the manufacturer’s protocol and purified via ethanol purification to obtain the resulting AF647-siRNA, as previously described.^[110]^ Calu3 monolayers were transfected with 100 pmol AF647-siRNA/P(SpAA-*co*-DAA)3 polyplexes at N/P 5 for 24 h. Afterward, monolayers were fixed in 4% PFA for 15 min, washed three times with PBS and permeabilized with PBS + 0.3% Tween 20 for 10 min. Cells were then incubated with rhodamine phalloidin (Life technologies, Carlsbad, USA) for 60 min. Finally, nuclei were stained with 4′,6-diamidino-2-phenylindole (DAPI) (Life technologies, Carlsbad, USA) at a concentration of 0.5 μg mL^−1^ for 15 min and washed two times with PBS. Samples were then mounted on glass slides using FluorSave reagent (Merck Millipore, Billerica, USA) prior to analysis with a SP8 inverted scanning confocal microscope (Leica Camera, Wetzlar, Germany). The images were exported from the Leica Image Analysis Suite (Leica) and processed with the Fiji distribution of ImageJ.

### Mucus staining

Calu3 monolayers were transfected with 100 pmol AF647-siRNA/P(SpAA-*co*-DAA)3 polyplexes at N/P 5 for 24 h. Once the incubation time was completed, cells were incubated for 15 min with AF488-wheat germ agglutinin (WGA) (Life technologies, Carlsbad, USA) at 37 °C and 5% CO_2_, washed with PBS twice and mounted on glass slides using Fluorsave. Monolayers were then immediately analyzed with a SP8 inverted scanning confocal microscope (Leica Camera, Wetzlar, Germany). The images were exported from the Leica Image Analysis Suite (Leica) and processed with the Fiji distribution of ImageJ.

### qPCR

Calu3 monolayers were transfected with 100 pmol siGAPDH/P(SpAA-*co*-DAA)3 polyplexes at N/P 5 and respective siNC as negative control for 24 h at 37 °C and 5% CO_2_. Lipofectamine lipoplexes were prepared as positive control. Cells were then lysed, and RNA was isolated using the PureLink RNA mini kit (Thermo Fischer Scientific, Waltham, USA) according to the manufacturer’s protocol with additional DNase digestion. Afterward, cDNA was synthetized using the high-capacity cDNA synthesis kit (Applied Biosystems, Waltham, Massachusetts). For qPCR, cDNA was diluted 1:10 and run with primers for GAPDH (Qiagen, Hilden, Germany) as well as *β*-actin (Qiagen, Hilden, Germany) for normalization. Cycle thresholds were acquired by auto setting within the qPCRsoft software (Analytic Jena, Jena, Germany). Values are given as mean ± SEM with n = 3. Two-way ANOVA with Bonferroni mean comparison (p > 0.05 considered not significant (ns); * *p* <0.05, **p <0.01, ***p <0.001 considered significantly different) was performed with OriginPro 2019b software (OriginLab, Northampton, Massachusetts).

### Methods for Ex Vivo Studies

*Preparation of Precision-Cut Lung slices*: All investigations using human tissue were approved by the ethics committee of the Hannover Medical School (MHH, Hannover, Germany) and are in compliance with “The Code of Ethics of the World Medical Association” (renewed on 2015/04/22, number 2701−2015). All patients gave written informed consent for the use of their lung tissue for research. PCLS were prepared from the lung explants of lung fibrosis patients. This human lung tissue from the peripheral lung was provided by the Hannover Medical School (MHH, Hannover, Germany) from three males with PF aged 58−63 years who underwent lung transplantation. Briefly, human lung lobes were cannulated with a flexible catheter and the selected lung segments were inflated with warm (37 °C) low melting agarose (1.5%) prepared in Dulbecco’s Modified Eagle’s Medium Nutrient Mixture F-12 Ham (DMEM-F-12) supplemented with 15 mm HEPES, 100 U mL^−1^ penicillin, and 100 μg mL^−1^ streptomycin (all from Invitrogen Life Technologies, Carlsbad, CA). After polymerization of the agarose solution on ice, tissue cores of a diameter of 8 mm were prepared using a sharp rotating metal tube. Subsequently, the cores were sliced into 250−300 μm thin slices in DMEM using a Krumdieck tissue slicer (Alabama Research and Development, Munford, AL). PCLS were washed 3x for 30 min in DMEM-F-12 supplemented with 15 mm HEPES, 100 U mL^−1^ penicillin, and 100 μg mL^−1^ streptomycin and used for the experiments.

### PAR2 Gene Silencing in PCLS

PCLS generated from three donor lungs were placed in 12-well-plates in 900 μL DMEM F-12 medium supplemented with HEPES and penicillin/streptomycin. Afterward, PCLS were transfected with P(SpAA-*co*-DAA)3 polyplexes at N/P 5 encapsulating 100 nmol pre-designed, commercially available siRNA against PAR2 (siPAR2; Santa Cruz Biotechnology, Santa Cruz, CA). To control for non-specific gene inhibition of the siRNA used in this study, a universal negative-control siRNA (siControl) sequence was employed (Ambion, Darmstadt, Germany). As additional controls, PCLS were transfected with lipoplexes of siLentFect lipid reagent (Bio-Rad Laboratories, Munich, Germany) using the same amount of siPAR2 or siControl as in the polyplexes. The tissue slices were incubated for 72 h at 37 °C and 5% CO_2_ in technical duplicates and afterward collected for protein isolation.

### Western Blot Analysis

PCLS were homogenized using a mortar and pestle in a lysis buffer containing 50 mm Tris, pH 7.4, 150 mm NaCl, 1 mm EDTA, 1% Triton-X-100, 1% Sodium Deoxycholate and 0.1% SDS supplemented with 1 mm Na_3_VO_4_,1 mm PMSF and1 μg mL^−1^ Complete Protease Inhibitor Cocktail (Roche Applied Science, Indianapolis, IN). Per lane, 10 μg protein were separated on a 10% SDS polyacrylamide gel and transferred to a PVDF membrane (Roth, Karlsruhe, Germany). The membrane was blocked with 1% bovine serum albumin (BSA, Sigma−Aldrich) and treated with either rabbit anti-PAR-2 antibody (cat. no.: 35−2300, Ther-moFisher Scientific, Dreieich, Germany) or goat anti-type I collagen antibody (cat. no.: 1310-01, SouthernBiotech, Birmingham, AL). Proteins were detected using the Amersham ECL Select Western Blotting Detection Reagent (GE Healthcare, Chicago, IL). The pictures were acquired using a ChemiDoc Imaging Systems (Bio-Rad). *β*-actin, detected using a mouse-anti *β*-actin antibody (Sigma−Aldrich), was used as a loading control.

### Two-Photon Laser Scanning Microscopy

For imaging analysis, PCLS were transfected with siGlo/P(SpAA-*co*-DAA)3 polyplexes at N/P 5 at a final concentration of 100 nm Cy3-labeled transfection marker siRNA (Horizon Discovery, Cambridge, UK). PCLS were transfected for 24 h and subsequently were placed inside a glass-bottom dish (glass thickness #1.5) with phenol-red-free culture medium. Hoechst was added in the imaging dish before the image acquisition. Images were captured using a Leica SP8 inverted microscope, equipped with a SpectraPhysics Insight X3 laser and external detectors. For the SHG signal as well as for the 2-photon excitation of Hoechst, the Insight laser was tuned at 860 nm, while for the excitation of the siGLO-Red transfection marker, the fixed line of the laser at 1045 nm was used. The SHG signal was captured with a spectral Hybrid Detector (HyD), which was set to detect wavelengths between 425 and 435 nm. The Hoechst emission together with the elastin fibers auto-fluorescence was captured with a second HyD from 440−460 nm, while the Cy3 emission of the siGLO-Red was captured with a photo multiplier tube from 565−655 nm. At this wavelength range, also elastin fiber auto-fluorescence was detected. All the emission wavelengths were captured with a 10 × 0.4NA air objective. The acquired z-stack images were subsequently computationally cleared and the channels’ cross-talk was removed using the Lightning and the Dye Separation software modules of Leica, respectively.

### Methods for In Vivo Studies

*Animals*: All animal experiments were carried out according to the German law of protection of animal life and approved by an external review committee for laboratory animal care by the Government of Upper Bavaria (approval number: ROB-55.2-2532.Vet_02-20-171).

Female Balb/c mice were purchased form Charles River Laboratories and used at 5 weeks of age. Mice were intratracheally instilled (under ketamine/xylazine anesthesia) with 2 nmol AF647-siRNA/P(SpAA-*co*- DAA)3 polyplexes at N/P 5 prepared in 50 μL 5% glucose as well as with siRNA/PEI polyplexes prepared in 50 μL 5% glucose. As negative control, animals were administered with 5% glucose only. After 48 h, mice were sacrificed and BALF as well as blood was collected.

BALF was obtained by lavaging lungs with 1 mL PBS/protease inhibitor and stored at −80 °C. The concentration of cytokines in BALF was determined using the mouse LEGENDplex ELISA kit (BioLegend, San Diego, California). Values are given as mean ± SEM with n = 4 for treatment with PEI or P(SPAA-*co*-DAA)3 polyplexes and n = 2 for untreated mice. One-way ANOVA with Bonferroni mean comparison (p > 0.05 considered not significant (ns); * *p* <0.05, **p <0.01, ***p <0.001 considered significantly different) was performed with OriginPro 2019b software (OriginLab, Northampton, Massachusetts).

Additionally, lung cell suspensions were prepared as previously reported.^[111]^ Lung cell suspensions were counterstained with BB515 labeled rat anti-mouse CD45 (30-F11, 1:40, BD Biosciences, Franklin Lakes, New Jersey), pacific orange labeled anti-F4/80 (1:100, Invitrogen, Waltham, Massachusetts), BV421 labeled anti-tubulin Beta 3 (1:100, BioLegend, San Diego, California), anti-prosurfactant protein C (1:100, Abcam), mouse anti-rabbit PE-Cy7 (1:200, Santa Cruz Biotechnology), antiuteroglobin antibody (1:100, Abcam) and mouse anti-rabbit AF594 (1:200, Invitrogen, Waltham, Massachusetts) following the manufacturer’s protocol. The analysis was performed on a Cytek Aurora flow cytometer (Cytek Biosciences, Fremont, California). The different populations of leukocytes (CD45+), macrophage/monocytes (F4/80+), Type II pneumocytes (proSPC+), ciliated cells (tubulin beta +) and club cells (uteroglobin +) were gated and the MFI of siRNA-AF647 in the different cell populations was quantified.

Values are given as mean ± SEM with n = 2 for treatment with P(SPAA-*co*-DAA)3 polyplexes. One-way ANOVA with Bonferroni mean comparison (p > 0.05 considered not significant (ns); * *p* <0.05, **p <0.01, ***p <0.001 considered significantly different) was performed with OriginPro 2019b software (OriginLab, Northampton, Massachusetts).

## Supplementary Material

Supplementary information

## Figures and Tables

**Table 1:** Free radical polymerization of NAS and DAA and copolymerizations of NAS and DAA.

Entry^a)^	Name	NAS/ DAA_Feed_ [mol%]	wt% AIBN	Time [h]	Yield [%]	*M*_n_ [x10^3^ g mol^-1^]^b)^	Ð^b)^	NAS/ DAA [mol%]^c)^	Name Postpoly.	n [x10^3^ g mol^-1^]^b)^	Ð^b)^	TBSpAA/DAA ratio^d)^	Name Deprot.	SpAA/DAA ratio^e)^
1	P(NAS)1-3	100/0	2	16.5	84	16.9	3.43	100/0	P(TBSpAA)1-3	104.9	2.75	100/0	P(SpAA)1-3	100/0
2		100/0	5	17	97	20.0	3.46	100/0		115.2	2.55	100/0		100/0
3		100/0	10	19	93	12.6	3.57	100/0		97.8	2.11	100/0		100/0
4	P(DAA)	0/100	2	16.5	80	23.7^f)^	2.02^f)^	0/100	-	−	−	−	−	−
5	P(NAS-co- DAA)1-3	90/10	10	22	80	20.0	3.40	91/9	P(TBSpAA-co- DAA)1-3	53.1	3.5	83/17	P(SpAA-co- DAA)1-3	83/17
6		80/20	10	21	72	29.3	2.80	83/17		82.5	2.61	72/28		76/24
7		50/50	10	20	87	35.3	3.69	49/51		129.5	1.74	40/60		43/57

a) Polymerization in toluene at 65 °C with monomer concentrations of 10 wt./vol% overnight;

b) *M*_n_ as obtained via SEC in DMF relative to polystyrene standards. Ð = *M*_w_/*M*_n_ calculated via SEC in DMF

c) Ratio between NAS/DAA calculated via ^1^H NMR spectroscopy in DMSO or CDCl_3_

d) Ratio between TBSpAA/DAA calculated via ^1^H NMR spectroscopy in CDCl_3_

e) Ratio between SpAA/DAA calculated via ^1^H NMR spectroscopy in D_2_O. Polyacrylamide fraction omitted for clarity: P(TBSpAA-co-DAA)1 = 10 wt%, P(TBSpAA-co-DAA)2 = 5 wt%, P(TBSpAA-co-DAA)3 = 1 wt%

f) *M*_n_ as obtained via SEC in chloroform relative to polystyrene standards. Ð = *M*_w_/*M*_n_

## Data Availability

The data that support the findings of this study are available in the supplementary material of this article.
