# Supplementary material for: Pulmonary siRNA Delivery with Sophisticated Amphiphilic Poly(Spermine Acrylamides) for the Treatment of Lung Fibrosis
Source: Small. Author manuscript; Available in PMC 2024 Oct 29. (PMC7616748; doi:10.1002/smll.202308775)
Supplement: Supplementary information [file EMS199502-supplement-Supplementary_information.pdf]

NANO · MICRO
small

Supporting Information

for *Small*, DOI 10.1002/smll.202308775

Pulmonary siRNA Delivery with Sophisticated Amphiphilic Poly(Spermine Acrylamides) for the Treatment of Lung Fibrosis

Friederike Adams, Christoph M. Zimmermann, Domizia Baldassi, Thomas M. Pehl, Philipp Weingarten, Iris Kachel, Moritz Kränzlein, David C. Jürgens, Peter Braubach, Ioannis Alexopoulos, Malgorzata Wygrecka and Olivia M. Merkel**

Supporting Information

Pulmonary siRNA delivery with sophisticated amphiphilic poly(spermine acrylamides) for the treatment of lung fibrosis

Friederike Adams, Christoph M. Zimmermann, Domizia Baldassi, Thomas M. Pehl, Philipp Weingarten, Iris Kachel, Moritz Kränzlein, David C. Jürgens, Peter Braubach, Ioannis Alexopoulos, Malgorzata Wygrecka, and Olivia M. Merkel[*]

1. Experimental section

Calculation of the Protonable Unit:

Hyperbranched PEI (25 kg/mol, Lupasol® WF, BASF, Ludwigshafen, Germany) has a protonable unit of 43.1 g/mol.

The protonable unit of the homopolymers was calculated by dividing the mass of the repeating unit by the number of protonable primary and secondary amines present in the polymer.

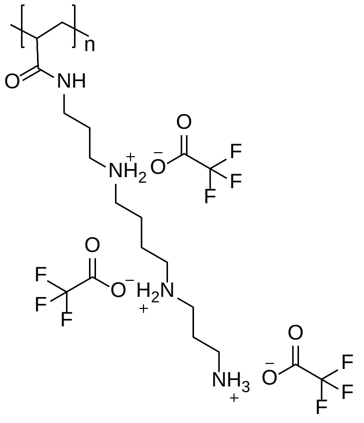

P(SpAA)

M_n = 598.46 g/mol
$M_{protonable\ unit}$ = 199.49 g/mol

Figure S 1 Structure and protonable unit of P(SpAA) 1-3.

The protonable unit of the different copolymers is calculated using the ratio of the two repeating units and the number of protonable primary and secondary amines present in the polymer.

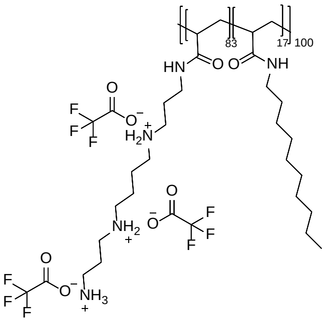

P(SpAA-*co*-DAA)1

M_n = 53265.13 g/mol
$M_{protonable\ unit}$ = 213.9 g/mol

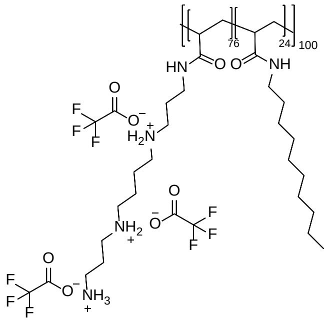

P(SpAA-*co*-DAA)2

M_n = 50555.36 g/mol
$M_{protonable\ unit}$ = 221.7 g/mol

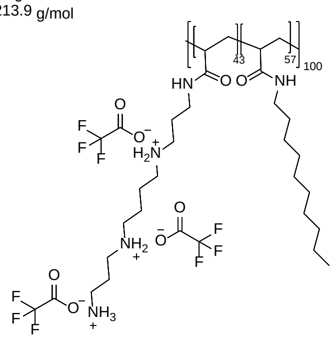

P(SpAA-*co*-DAA)3

M_n = 37780.73 g/mol
$M_{protonable\ unit}$ = 292.9 g/mol

Figure S 2: Structure and protonable unit of P(SpAA-*co*-DAA) 1-3.

Monomer reactivity ratios of NAS and DAA.

The Fineman-Ross equations (*Equation (1)* and *Equation (2)*) were used to calculate the reactivity ratios of a NAS or DAA-polymer chain ends towards reaction with an NAS or DAA monomer,

$$\frac{F}{f}(f-1) = r_1\frac{F^2}{f} - r_2 \qquad (1)$$

$$\frac{f-1}{F} = -r_2\frac{f}{F^2} + r_1 \qquad (2)$$

in which F is the monomer ratio in feed ($F = M_1/M_2$; NAS (M_1) and DAA (M_2)) and f is the monomer ratio in the polymer ($f = m_1/m_2$; NAS (m_1) and DAA (m_2)). Furthermore, r_1 and r_2 are the reactivity ratios of NAS and DAA, respectively. The monomer reactivity ratios are given by $r_1 = k_{11}/k_{12}$ and $r_2 = k_{22}/k_{21}$.

The reactivity ratios are obtained from calculating the slopes by plotting (F^2/f) against (F/f)(f-1) and (f/F^2) against (f-1)/F for the determination of r_1 and r_2, respectively (Figure 1).[1]

2. Characterization and synthesis of 1-4

N-Acryloxysuccinimide (NAS) (1):[2]

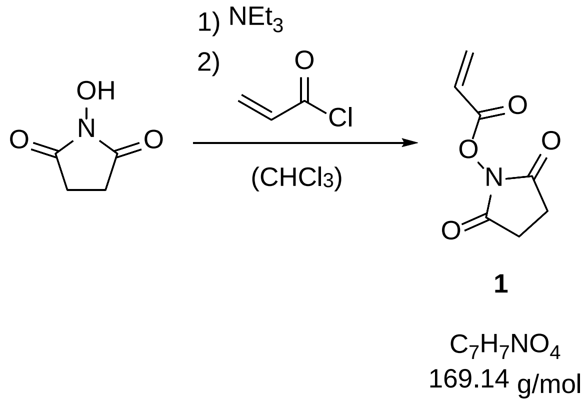

N-Hydroxysuccinimide (5.30 g, 46.0 mmol, 1.00 eq.) was dissolved in 60 mL chloroform. Triethylamine (7.30 mL, 52.6 mmol, 1.15 eq.) was added, the solution was cooled to 0 °C and acryloyl chloride (4.25 mL, 52.6 mmol, 1.15 eq.) was added dropwise. After stirring for 4 h at room temperature, the resulting reaction mixture was extracted with water (2 x 50 mL), the organic phase was dried over anhydrous $MgSO_4$, filtered and concentrated *in vacuo*. After recrystallization from hexane/EtOAc (8:1), the product was obtained as a colorless crystalline substance (5.26 g, 71%).

^{1}H NMR (400 MHz, $CDCl_3$, 298 K): δ (ppm) = 6.69 (dd, J = 17.3, 0.9 Hz, 1H, H_{Alkene}), 6.32 (dd, J = 17.3, 10.7 Hz, 1H, H_{Alkene}), 6.16 (dd, J = 10.7, 0.9 Hz, 1H, H_{Alkene}), 2.85 (s, 4H, H_{NHS}).

^{13}C NMR (101 MHz, $CDCl_3$, 298 K): δ (ppm) = 169.2, 161.2, 136.3, 123.1, 25.7.

**EA:**	calculated:	C 49.71	H 4.17	N 8.29
	found:	C 49.65	H 4.19	N 8.34

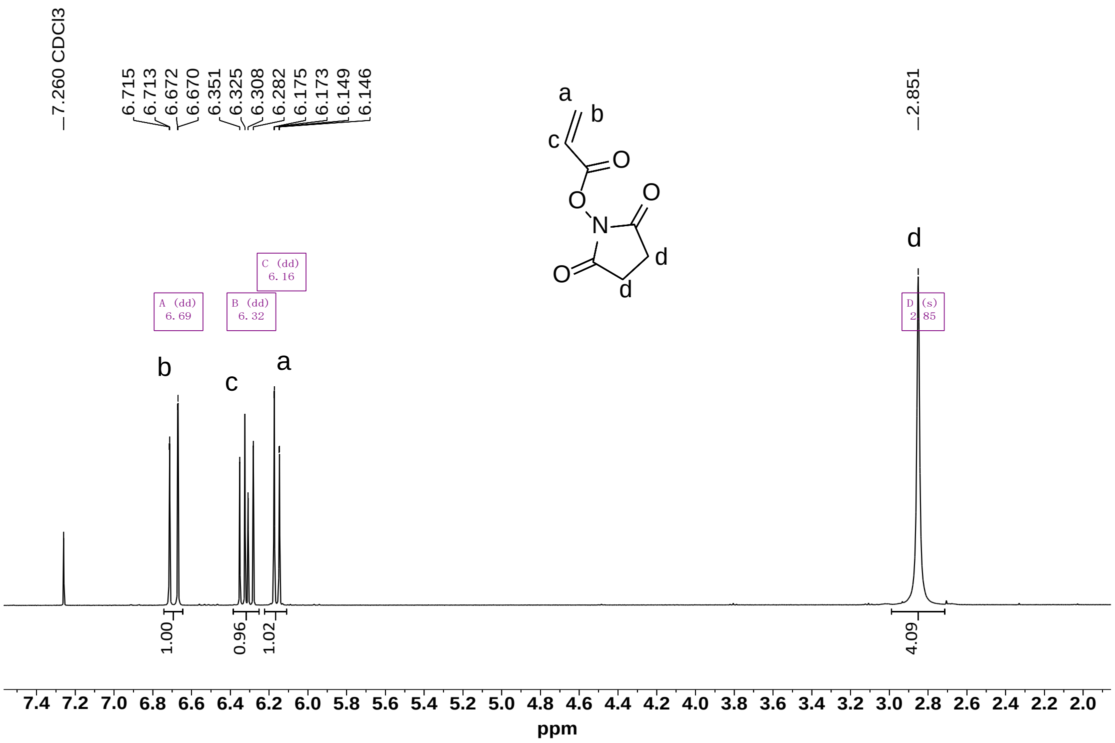

Figure S 3: ^{1}H-NMR spectrum of NAS (1) in $CDCl_3$ (400 MHz).

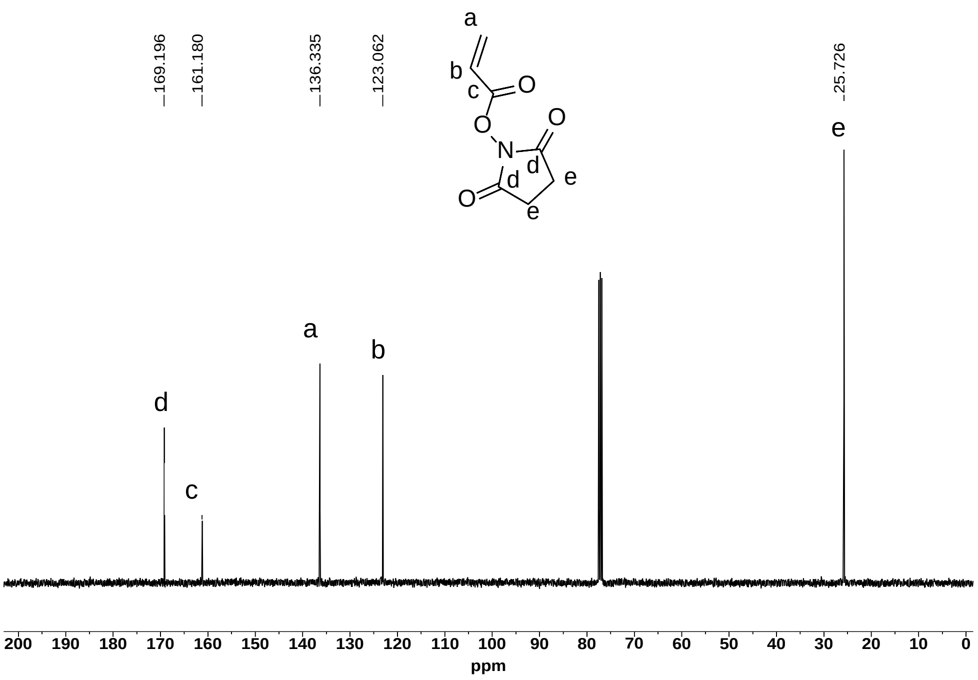

Figure S 4: ^{13}C-NMR spectrum of NAS (1) in $CDCl_3$ (101 MHz).

N-Decylacrylamide (DAA)(2):[3]

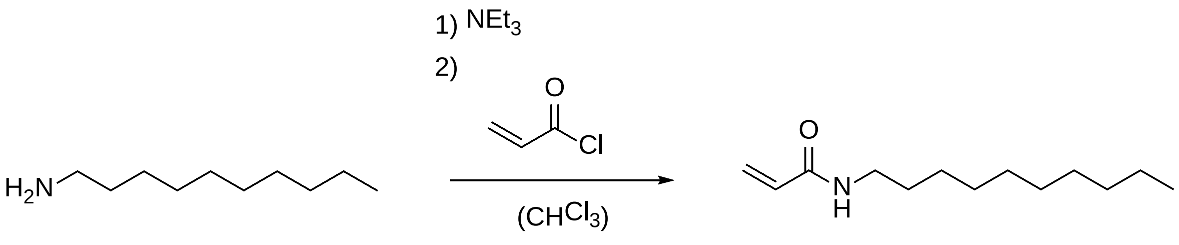

2
$C_{13}H_{25}NO$
211.35 g/mol

N-Decylamine (8.00 g, 50.9 mmol, 1.00 eq.) was dissolved in 50 mL chloroform. Triethylamine (7.75 mL, 55.9 mmol, 1.10 eq.) was added, the solution was cooled to 0 °C and acryloyl chloride (4.52 mL, 55.9 mmol, 1.10 eq.) was added dropwise. After stirring for 1 h at 0 °C and 4 h at room temperature, the resulting reaction mixture was extracted with saturated NH_4Cl aqueous solution (1 x 40 mL) and saturated NaCl aqueous solution (1 x 40 mL), the organic phase was dried over anhydrous $MgSO_4$, filtered and concentrated *in vacuo*. After recrystallization from heptane, the product was obtained as a colorless crystalline substance (7.02 g, 65%).

^{1}H NMR (400 MHz, $CDCl_3$, 298 K): δ (ppm) = 6.25 (dd, J = 17.0, 1.5 Hz, 1H, H_{Alkene}), 6.08 (dd, J = 17.0, 10.3 Hz, 1H, H_{Alkene}), 5.81 – 5.68 (broad, 1H, NH), 5.61 (dd, J = 10.3, 1.5 Hz, 1H, H_{Alkene}), 3.34 – 3.28 (m, 2H, CH_2-NH-C(O)), 1.52 (p, 2H, CH_2), 1.32 – 1.21 (m, 14H, CH_2), 0.87 (t, 3H, CH_3).

^{13}C NMR (101 MHz, $CDCl_3$, 298 K): δ (ppm) = 165.7 (C=O), 131.1 (CH=), 126.2 (CH_2=), 39.8 (CH_2N), 32.0 (CH_2), 29.6 (2xCH_2), 29.4 (2xCH_2), 27.1 (CH_2), 22.7 (CH_2), 14.2 (CH_3).

**EA:**	calculated:	C 73.88	H 11.92	N 6.63
	found:	C 73.15	H 11.72	N 6.60

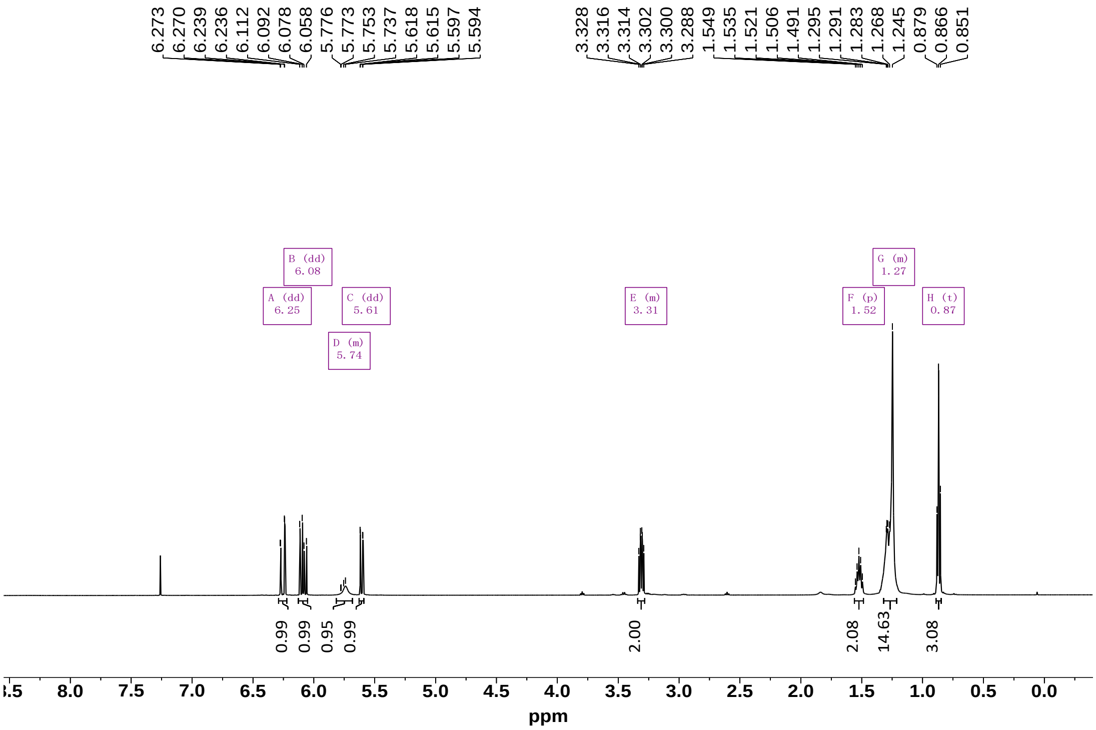

Figure S 5: ^{1}H-NMR spectrum of DAA (2) in $CDCl_3$ (400 MHz).

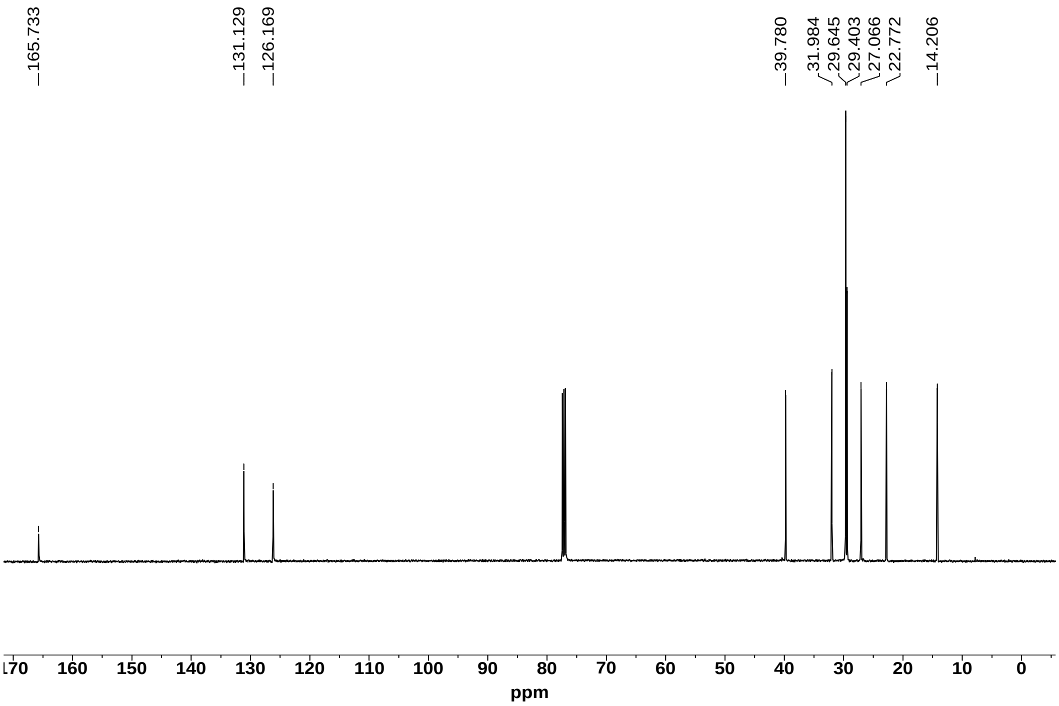

Figure S 6: ^{13}C-NMR spectrum of DAA (2) in $CDCl_3$ (101 MHz).

Tri-boc spermine (TBSp) (3):[4]

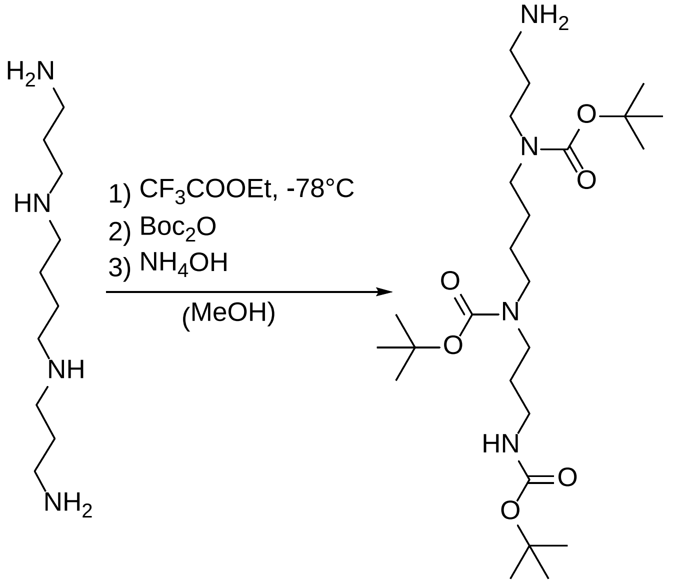

3

$C_{25}H_{50}N_4O_6$
502.70 g/mol

Spermine (4.00 g, 19.8 mmol, 1.0 eq) was dissolved in anhydrous MeOH (250 mL). The solution was cooled down to –78°C and 2.81 g Ethyltrifluoroacetate (2.35 mL, 19.8 mmol, 1.0 eq) were added dropwise. Afterwards, the reaction mixture was stirred at –78°C for 1 h and then at 0°C for 1 h. Di-tert-butyl dicarbonate (17.3 g, 79.1 mmol, 4.0 eq) was dissolved in 50 mL MeOH and added to the solution. After stirring for one day at room temperature, the pH was increased to 11 with concentrated aqueous ammonia and the solution was stirred at room temperature overnight. The solvent was removed under reduced pressure and the crude was purified via column chromatography (SiO_2, CH_2Cl_2/MeOH/$NH_{3,aq}$ = 70:10:1 to 50:10:1) to obtain the desired tri-boc spermine **3** as a colorless viscous oil (4.80 g, 9.54 mmol, 48%).

$\mathbf{R_f}$ = 0.27 (SiO_2, $KMnO_4$, CH_2Cl_2/MeOH/NH_3 = 10:1:0.1).

^{1}H NMR (500 MHz, $CDCl_3$, 298 K): δ (ppm) = 3.32 – 2.90 (m, 10H, 5xCH_2N), 2.80 (s, 2H, CH_2), 1.78 (s, 2H, CH_2), 1.55 (s, 2H, CH_2), 1.49 – 1.19 (m, 31H, 2xCH_2, 9x$CH_{3,Boc}$).

**HR-MS (ESI+):**	Calculated:	m/z 503.38086 ($C_{25}H_{51}O_6N_4$ $[M+H]^+$)
	Found:	m/z 503.38026

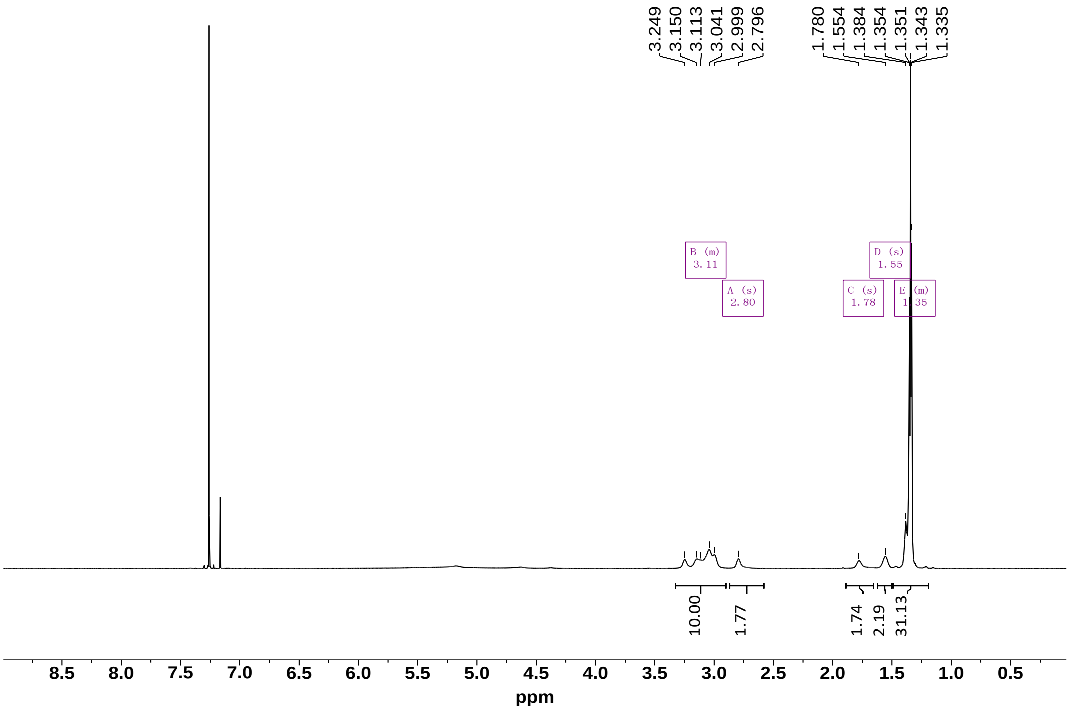

Figure S 7: ^{1}H-NMR spectrum of TBSp (3) in $CDCl_3$ (400 MHz).

Propionyl-spermine TFA-salt (4)

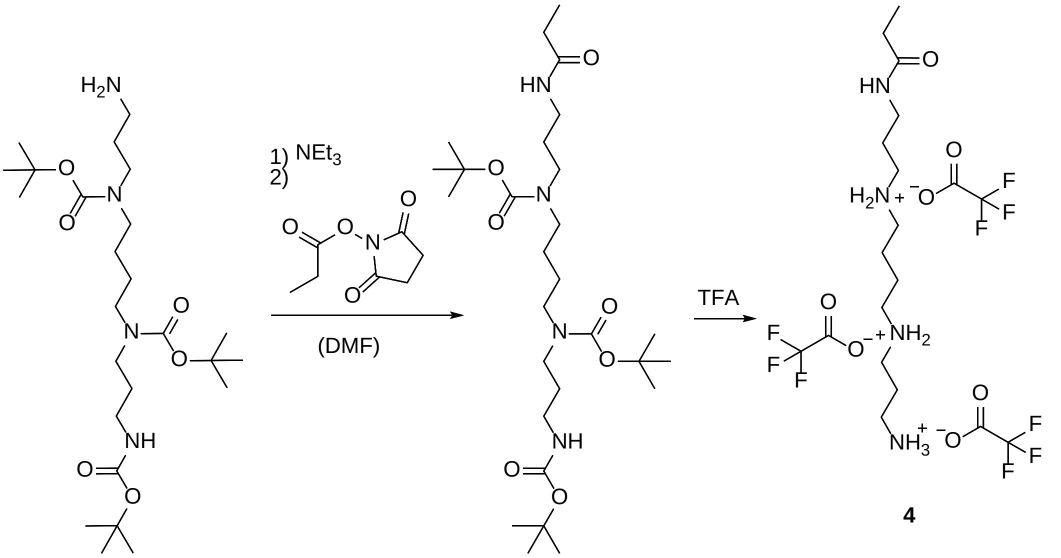

4

Tri-boc spermine **3** (410 mg, 0.82 mmol, 1.0 eq.) was dissolved in 5 mL anhydrous dimethylformamide and 0.32 mL triethylamine (330 mg, 3.26 mmol, 4.0 eq.) were added. 140 mg propionyl-NHS ester (0.82 mmol, 1.0 eq.) in 0.8 mL dimethylformamide were slowly added to the reaction mixture at room temperature. After stirring for 4 h, the solvent was evaporated and the crude product was purified via column-chromatography (SiO_2, CH_2Cl_2/MeOH/$NH_{3,aq}$ = 4:1:0.1) to obtain propionyl tri-boc spermine.

^{1}H NMR (500 MHz, $CDCl_3$, 298 K): δ (ppm) = 3.28 – 2.94 (m, 12H), 2.15 (q, J = 7.6 Hz, 2H), 1.58 (s, 4H), 1.38 (m, 31H), 1.09 (t, J = 7.6 Hz, 3H).

ESI-MS (Acetonitrile/H_2O): 581.4 $[M+Na]^+$

Propionyl tri-boc spermine is treated with 2 mL trifluoroacetic acid for 2 hours at room temperature. The reaction mixture is precipitated from diethyl ether and freeze-dried from water to give propionyl spermine **4** as a colorless powder.

^{1}H NMR (400 MHz, D_2O, 298 K): δ (ppm) = 3.28 (t, J = 6.7 Hz, 2H), 3.18 – 2.97 (m, 10H), 2.25 (q, J = 7.7 Hz, 2H), 2.14 – 2.01 (m, 2H), 1.88 (dq, J = 8.4, 6.9 Hz, 2H), 1.76 (p, J = 3.5 Hz, 4H), 1.09 (t, J = 7.7 Hz, 3H).

^{13}C NMR (101 MHz, D_2O, 298 K): δ (ppm) = 178.6 (NH-C=O), 162.9 (q, J = 35.6 Hz, $F_3CC(O)$=O), 116.3 (q, J = 291.5 Hz, $F_3CC(O)$=O), 46.9, 46.8, 44.9, 44.4, 36.4, 35.8, 29.0, 25.6, 23.7, 22.7, 9.5.

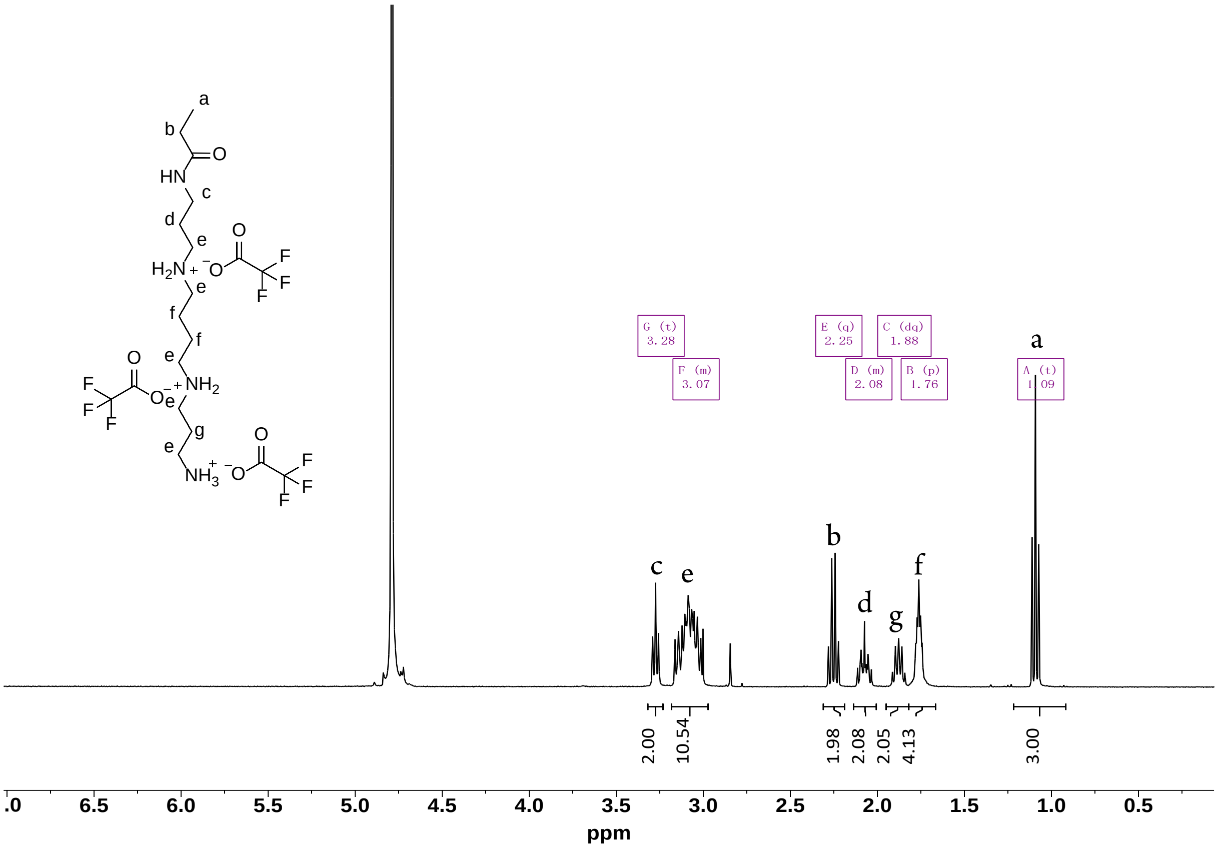

Figure S 8: ^{1}H-NMR spectrum of 4 in D_2O (400 MHz).

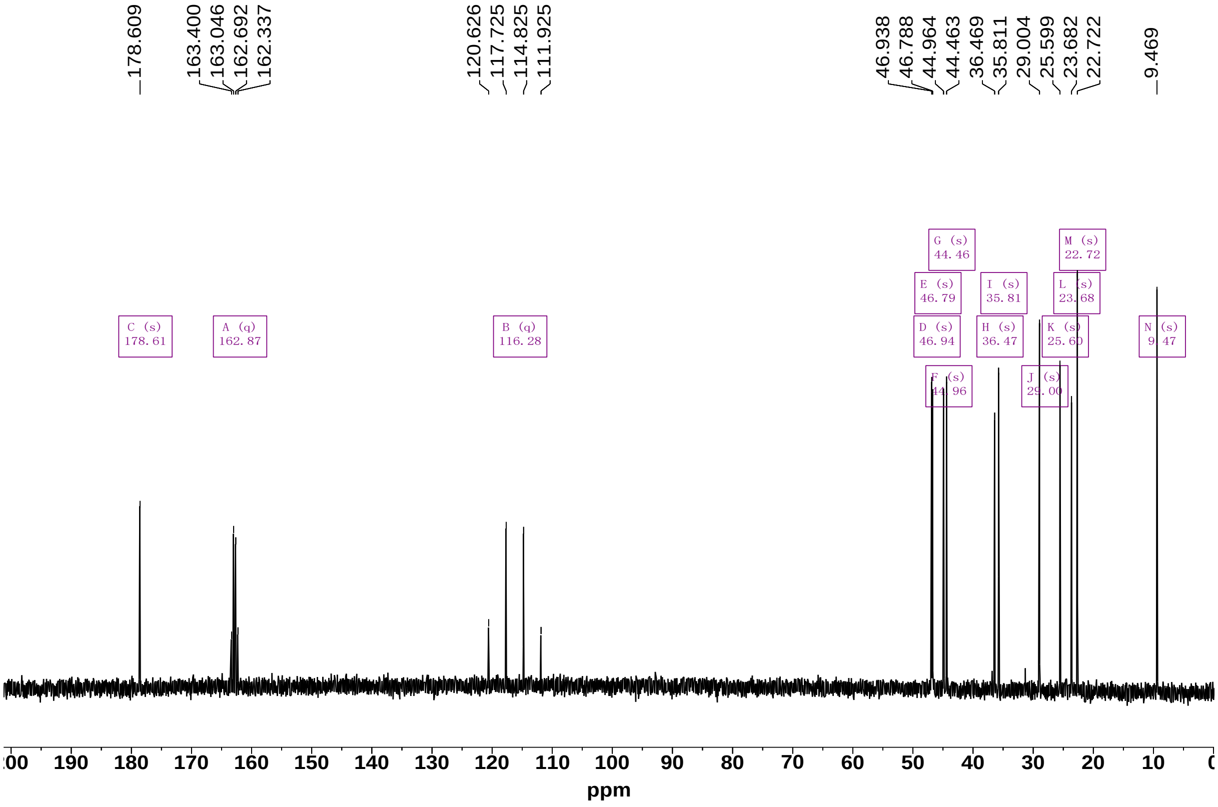

Figure S 9: ^{13}C-NMR spectrum of 4 in D_2O (101 MHz).

3. Copolymerization Parameter

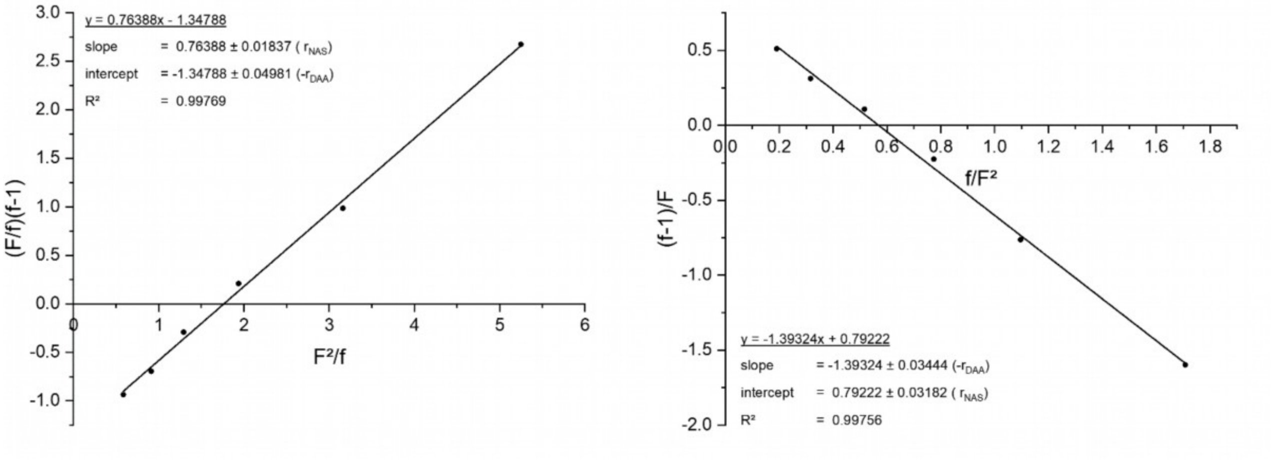

Figure S 10: **Determination of copolymerization reactivity ratios according to Fineman-Ross technique.[24]**

4. NMR spectra of polymers

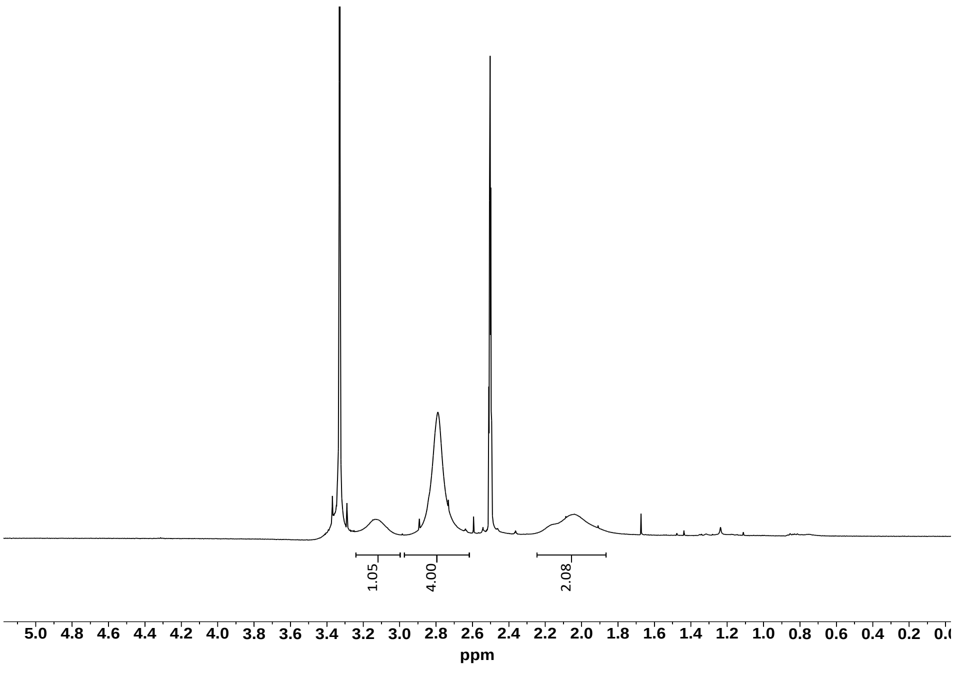

Figure S 11: ^{1}H-NMR spectrum of P(NAS) in DMSO (500 MHz).

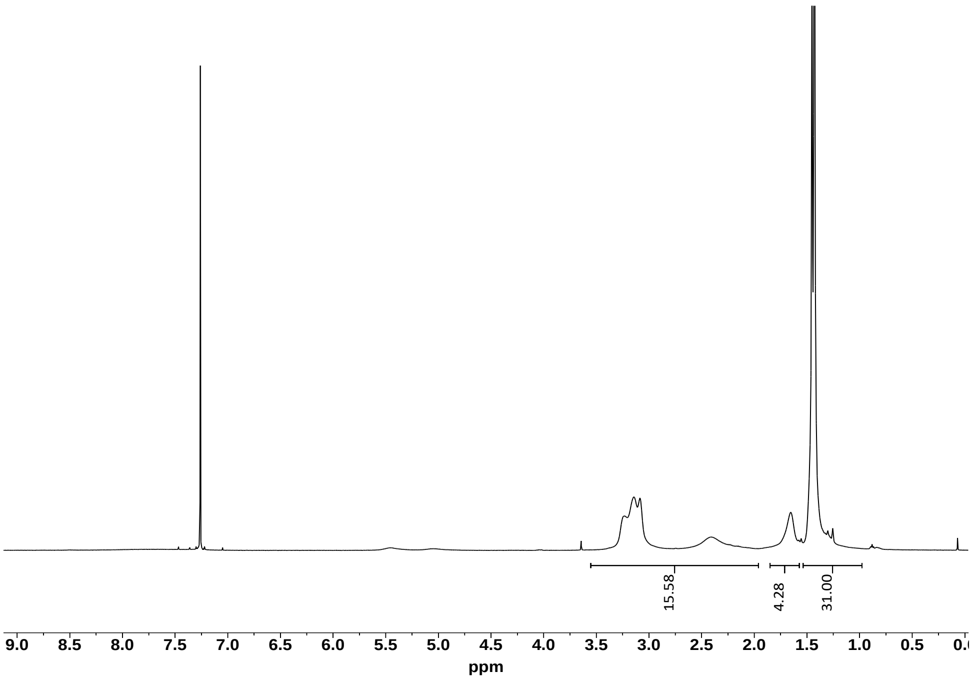

Figure S 12: ^{1}H-NMR spectrum of P(TBSpAA) in $CDCl_3$ (500 MHz).

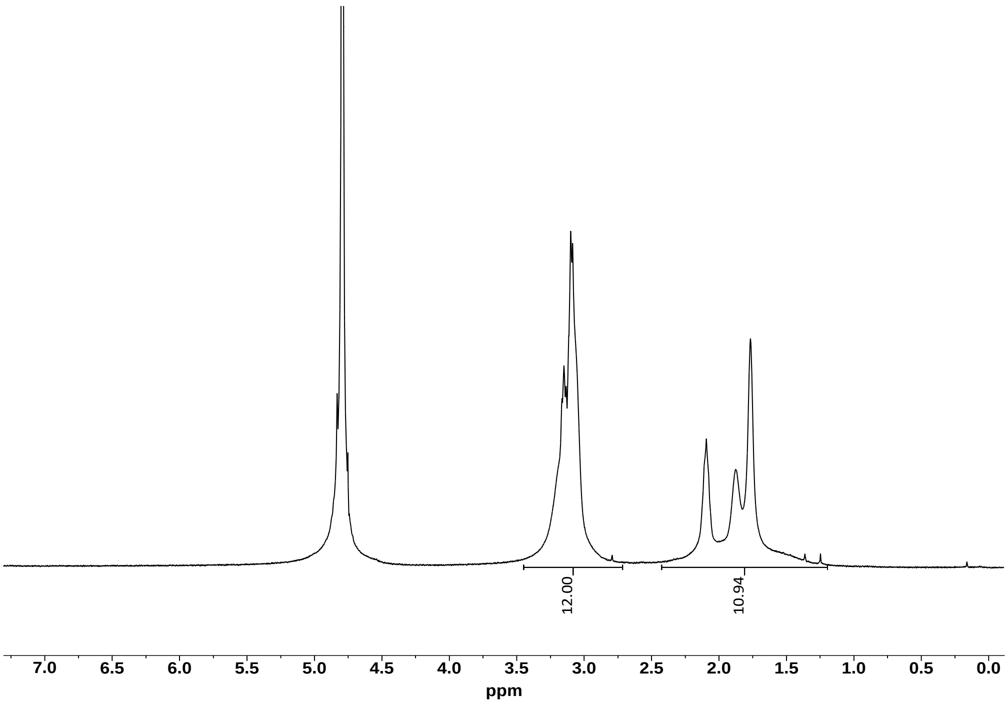

Figure S 13: ^{1}H-NMR spectrum of P(SpAA) TFA salt in D_2O (500 MHz).

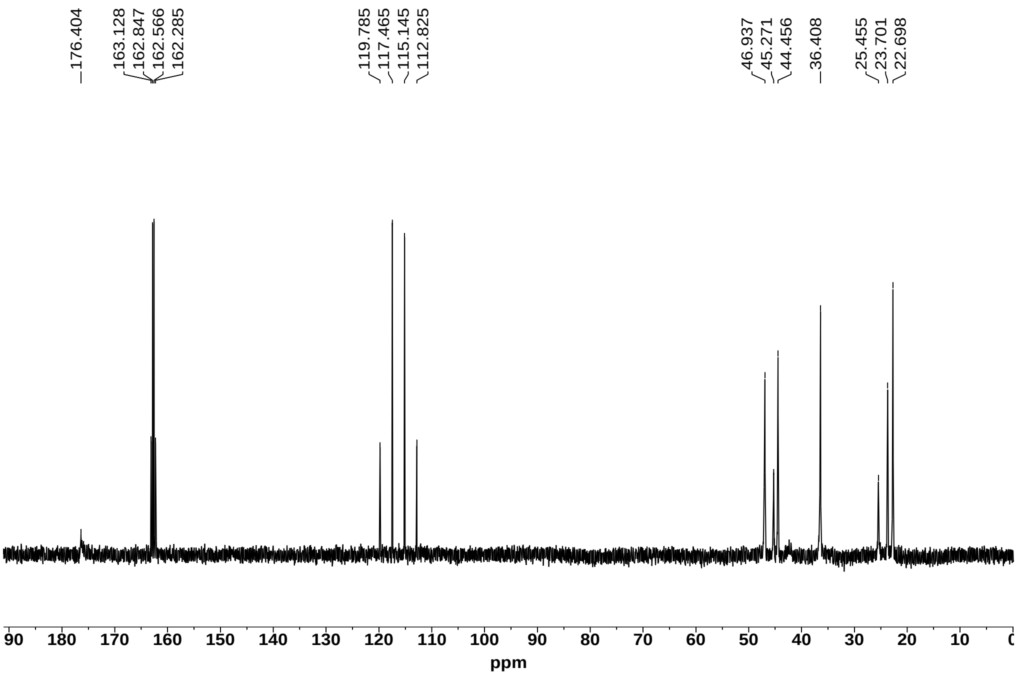

Figure S 14: ^{13}C-NMR spectrum of P(SPAA) TFA salt in D_2O (101 MHz).

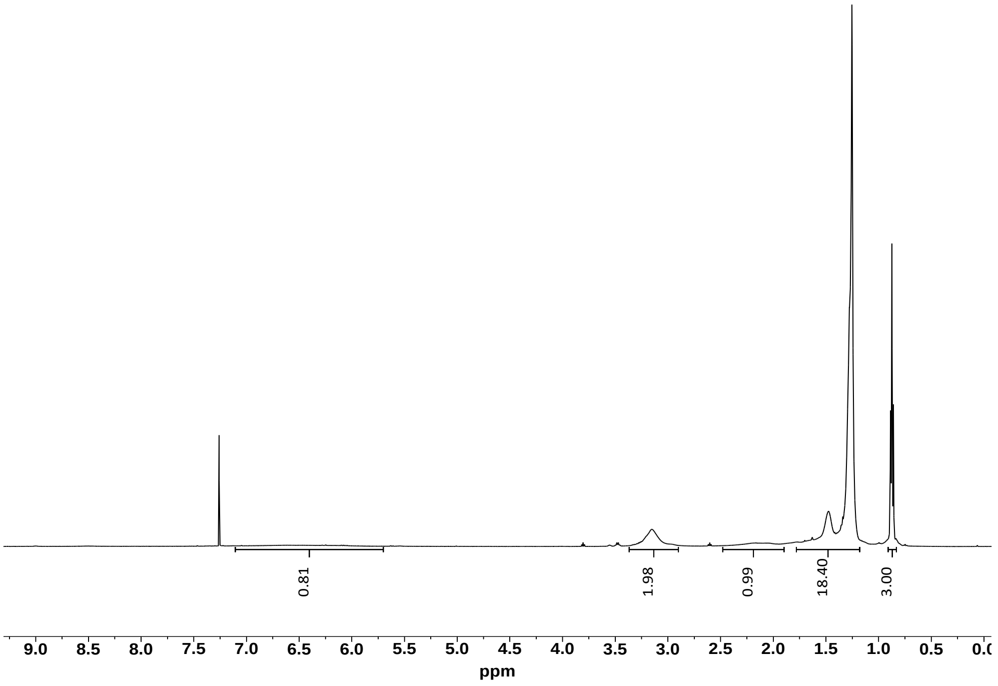

Figure S 15: ^{1}H-NMR spectrum of P(DAA) in $CDCl_3$ (500 MHz).

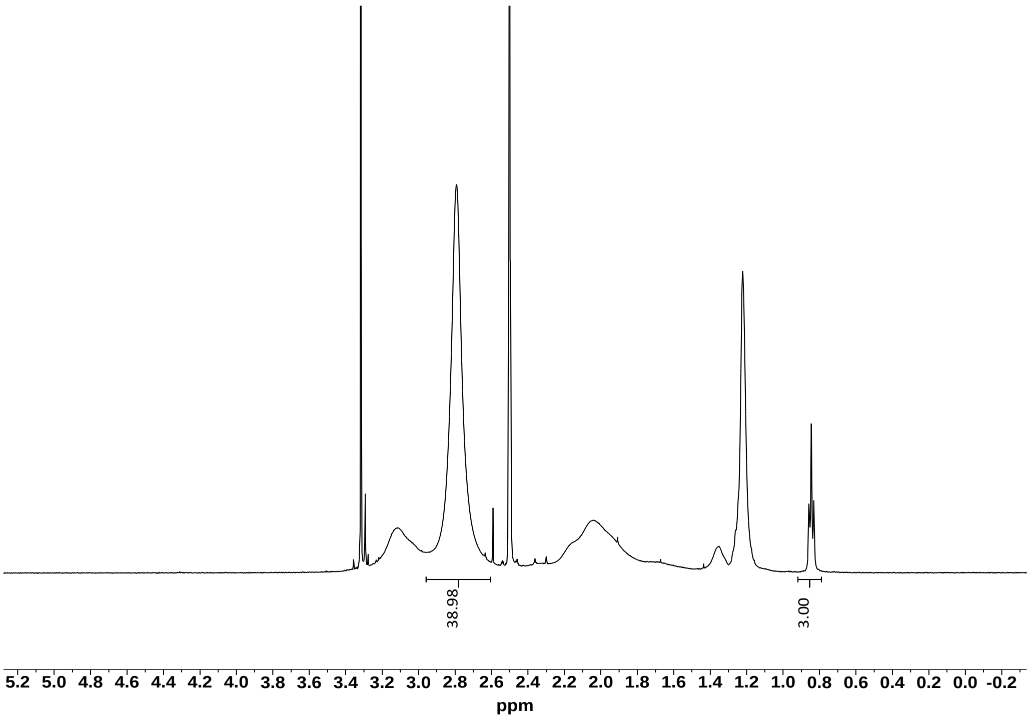

Figure S 16: ^{1}H-NMR spectrum of P(NAS-*co*-DAA)1 in DMSO (500 MHz).

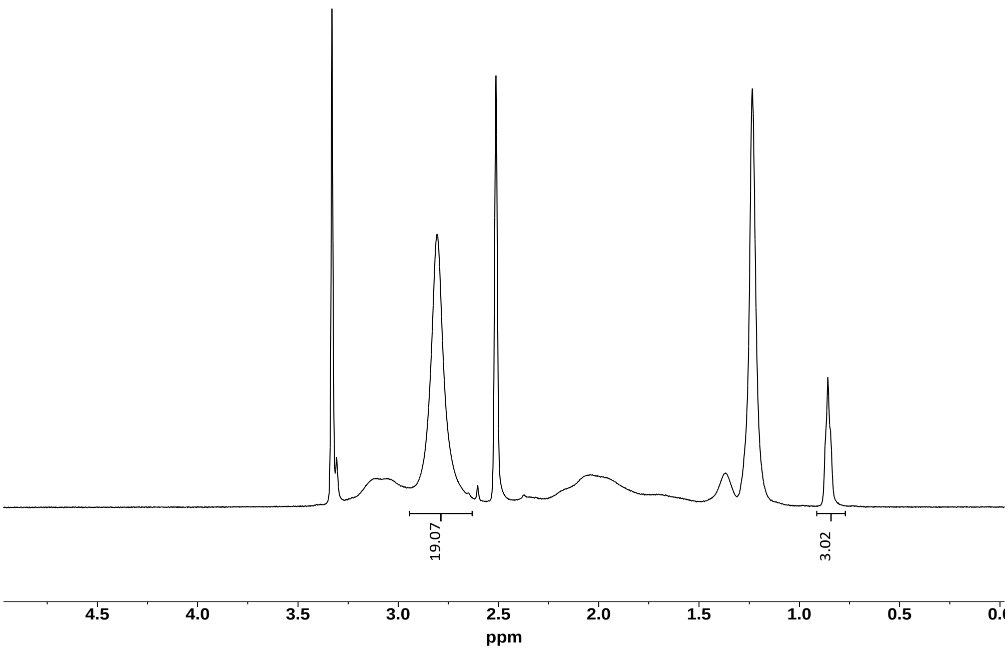

Figure S 17: ^{1}H-NMR spectrum of P(NAS-*co*-DAA)2 in DMSO (500 MHz).

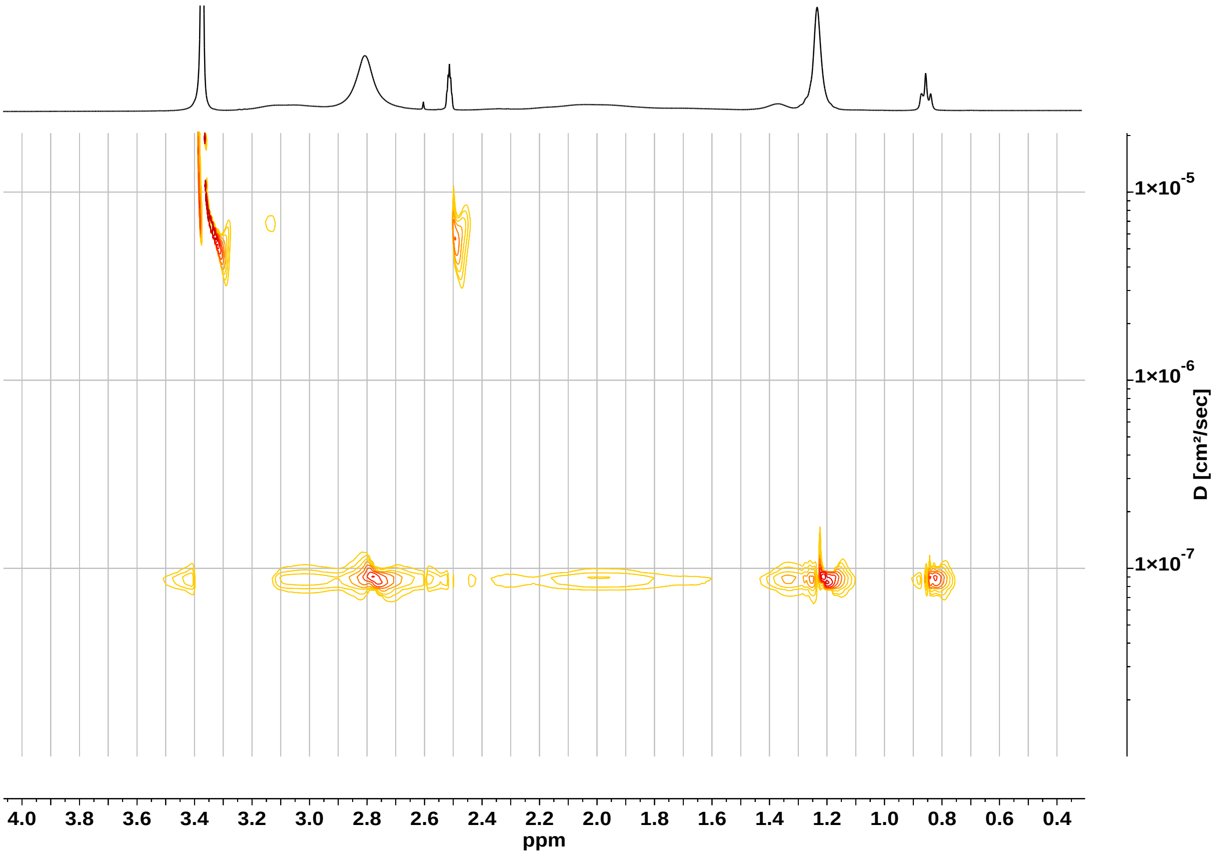

Figure S 18: DOSY-NMR spectrum of P(NAS-*co*-DAA)2 in DMSO (400 MHz, 32 scans, resolution factor; 1, repetitions:1, points in diffusion dimension: 128).

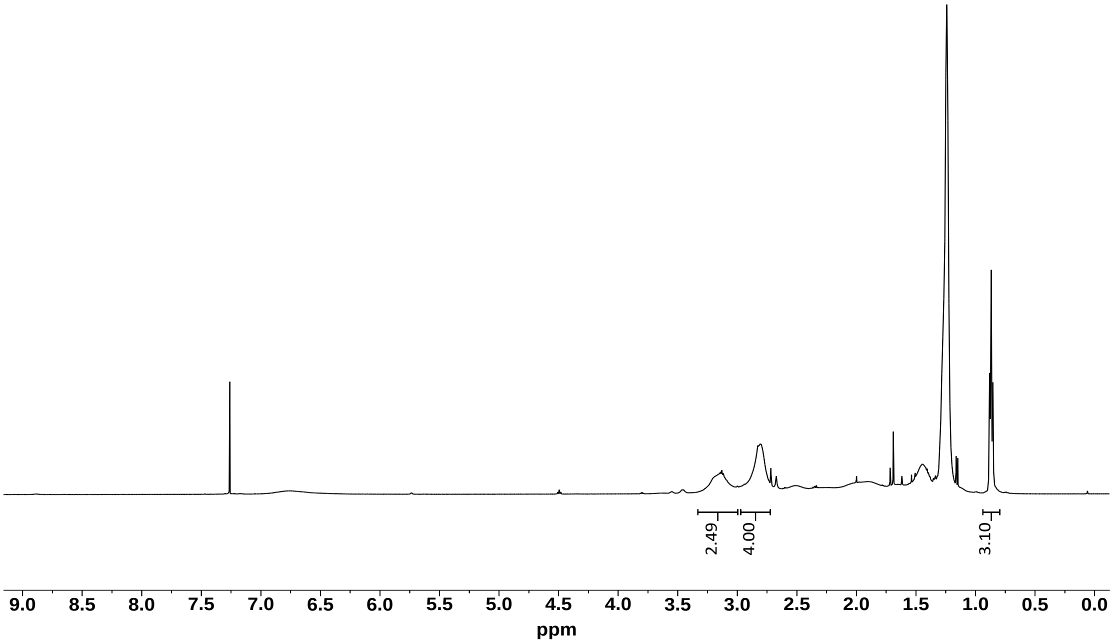

Figure S 19: ^{1}H-NMR spectrum of P(NAS-*co*-DAA)3 in $CDCl_3$ (500 MHz).

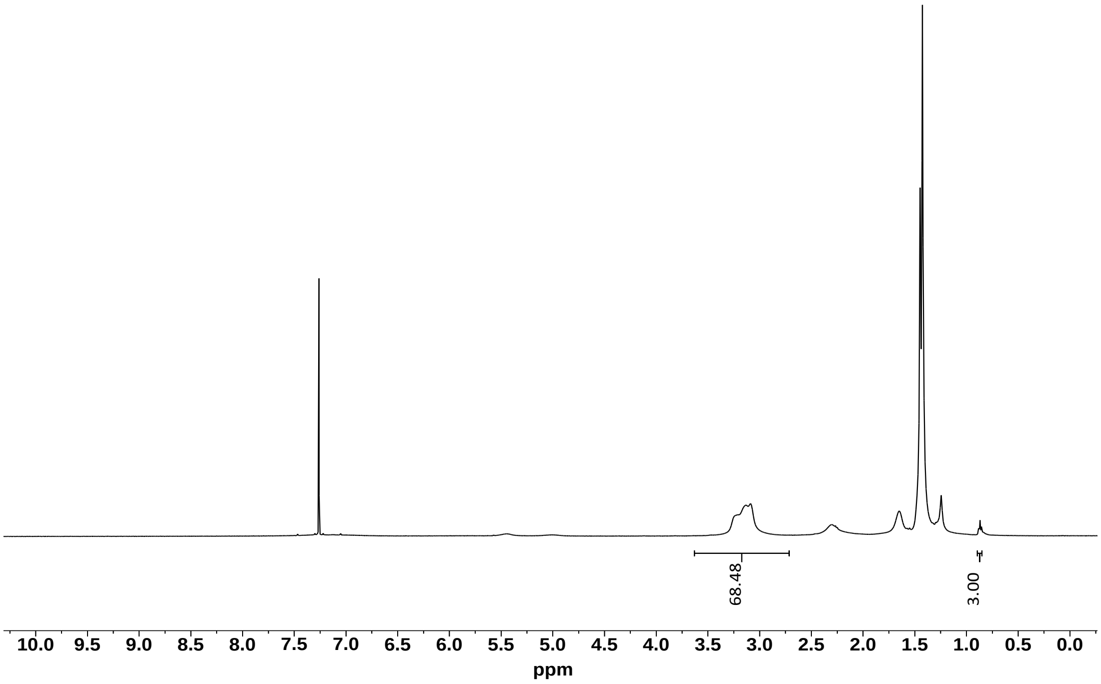

Figure S 20: ^{1}H-NMR spectrum of P(TBSpAA-*co*-DAA)1 in $CDCl_3$ (500 MHz).

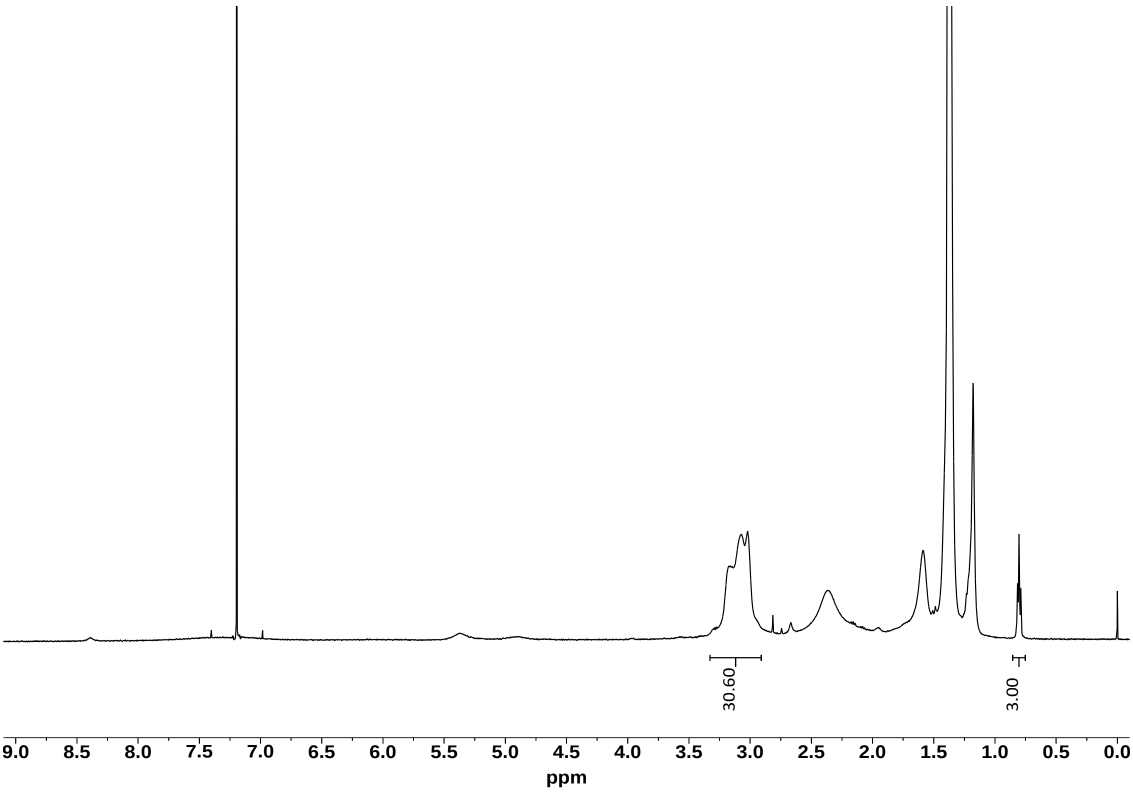

Figure S 21: ^{1}H-NMR spectrum of P(TBSpAA-*co*-DAA)2 in $CDCl_3$ (500 MHz).

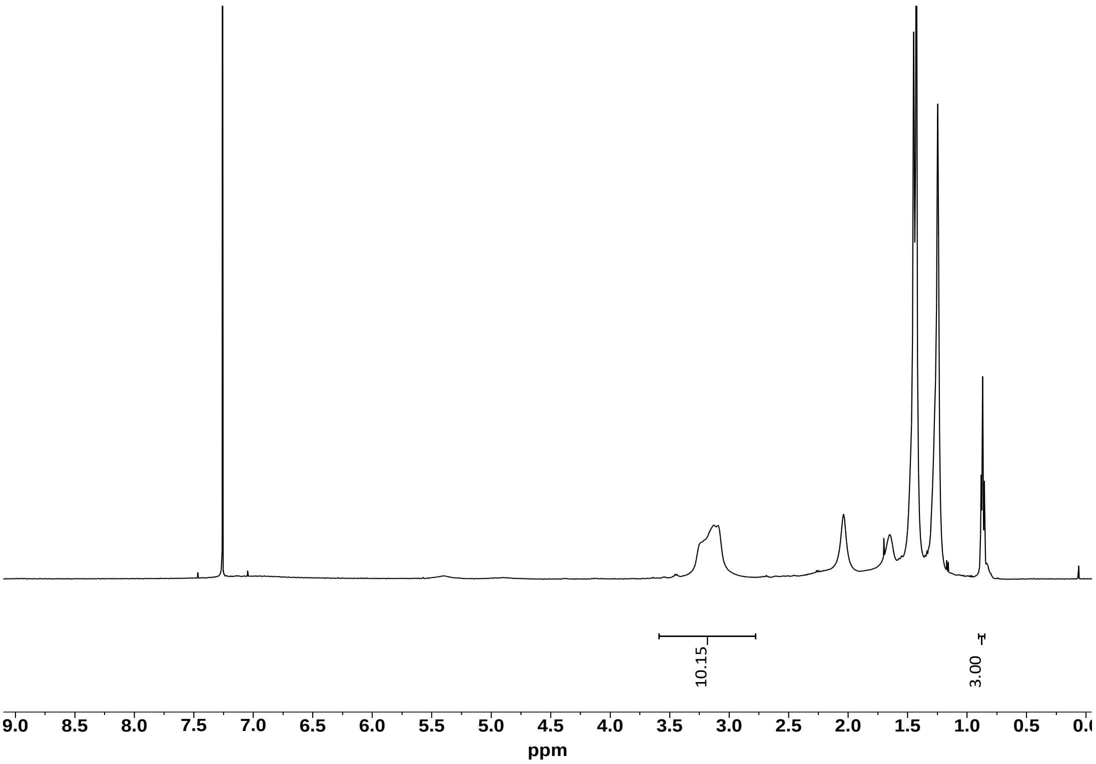

Figure S 22: ^{1}H-NMR spectrum of P(TBSpAA-*co*-DAA)3 in $CDCl_3$ (500 MHz).

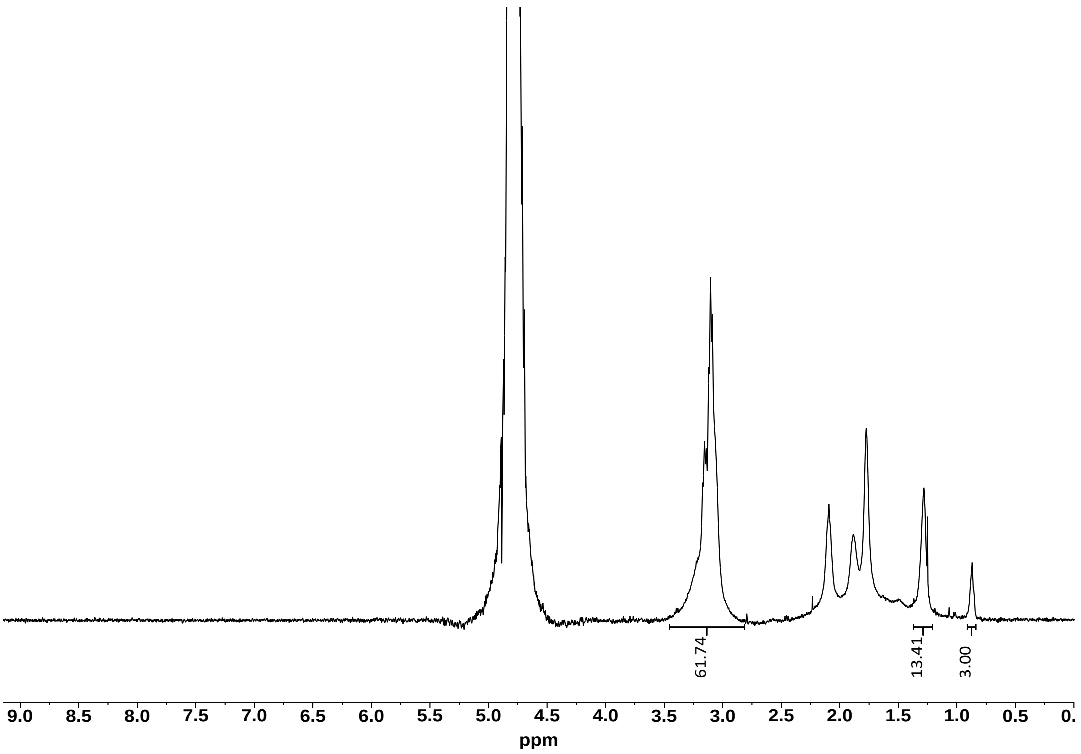

Figure S 23: ^{1}H-NMR spectrum of P(SpAA-*co*-DAA)1 TFA salt in D_2O (500 MHz).

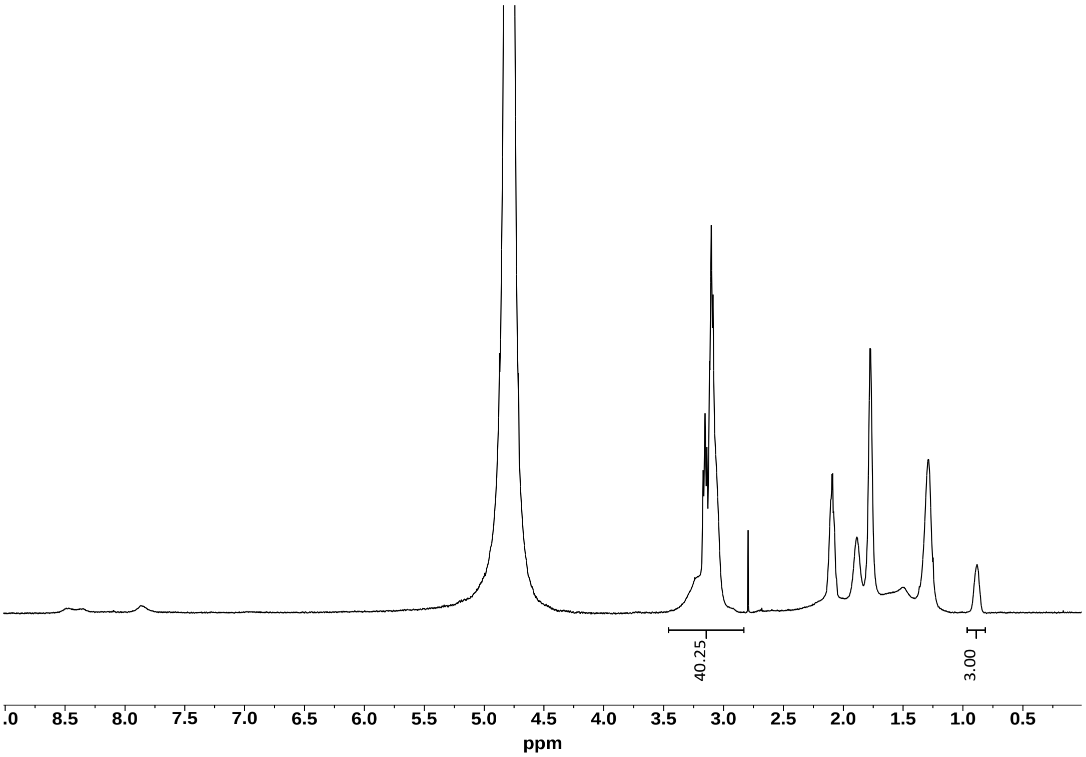

Figure S 24: ^{1}H-NMR spectrum of P(SpAA-*co*-DAA)2 TFA salt in D_2O (500 MHz).

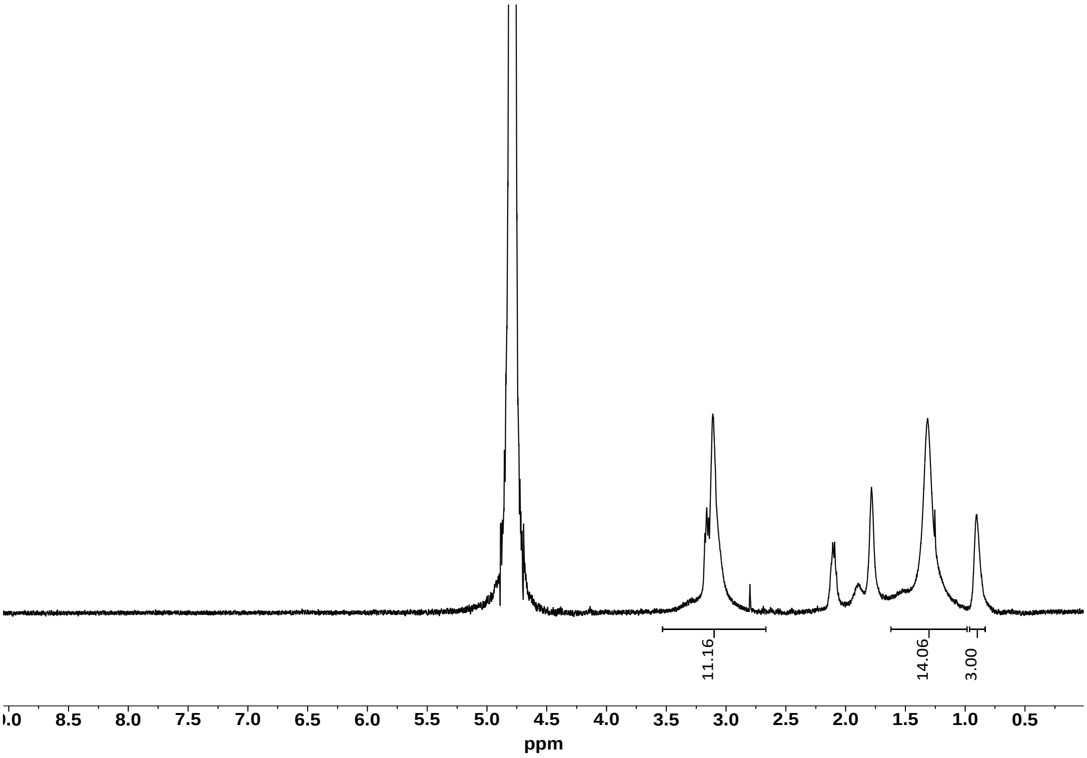

Figure S 25: ^{1}H-NMR spectrum of P(SpAA-*co*-DAA)3 TFA salt in D_2O (500 MHz).

5. SEC-traces

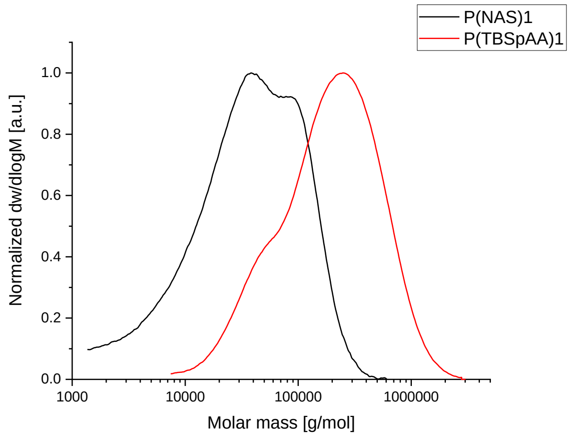

Figure S 26: SEC traces of P(NAS)1 and P(TBSpAA)1 measured via SEC in DMF.

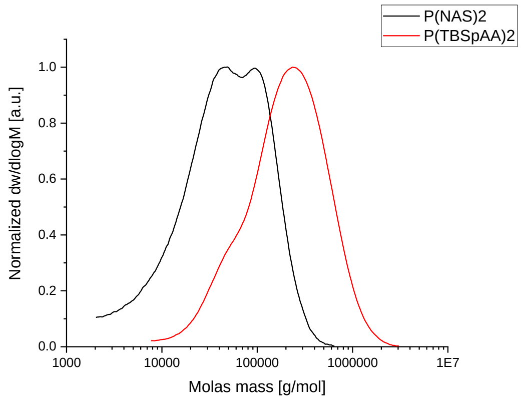

Figure S 27: SEC traces of P(NAS)2 and P(TBSpAA)2 measured via SEC in DMF.

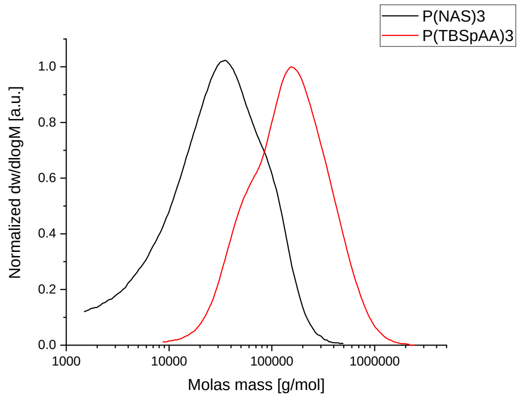

Figure S 28: SEC traces of P(NAS)3 and P(TBSpAA)3 measured via SEC in DMF.

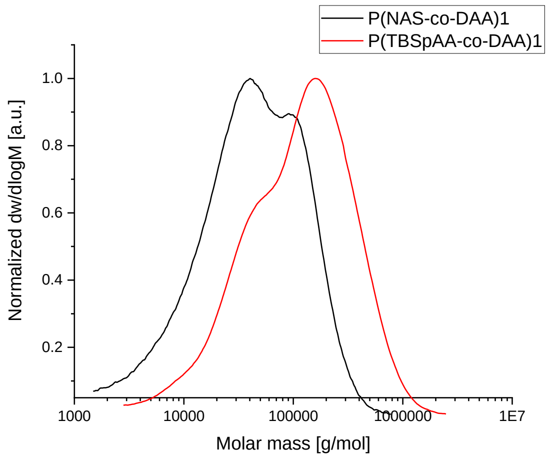

Figure S 29: SEC traces of P(NAS-*co*-DAA)1 and P(TBSpAA-*co*-DAA)1 measured via SEC in DMF.

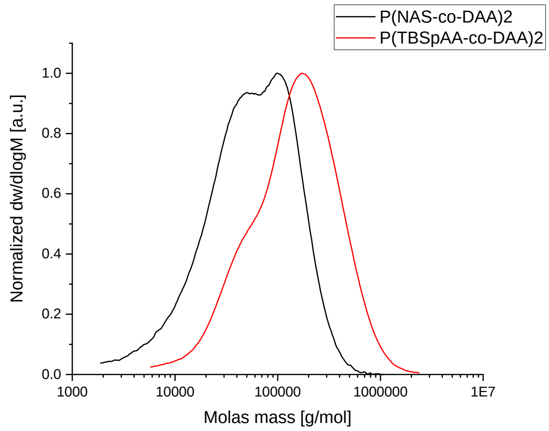

Figure S 30: SEC traces of P(NAS-*co*-DAA)2 and P(TBSpAA-*co*-DAA)2 measured via SEC in DMF.

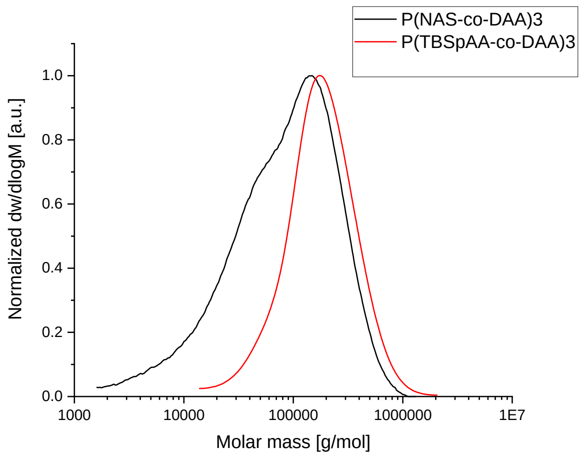

Figure S 31: SEC traces of P(NAS-*co*-DAA)3 and P(TBSpAA-*co*-DAA)3 measured via SEC in DMF.

6. Titration curve

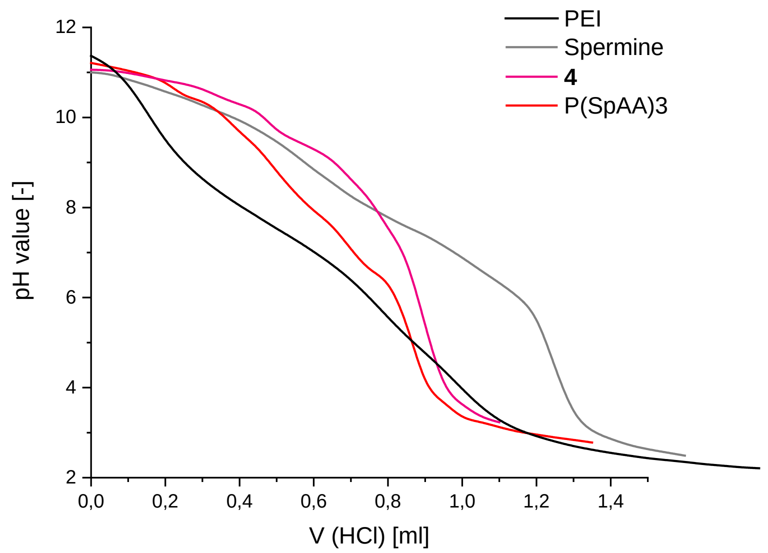

Figure S 32: Titration curves of PEI, Spermine, 4 and P(SpAA)3.

7. CAC calculations

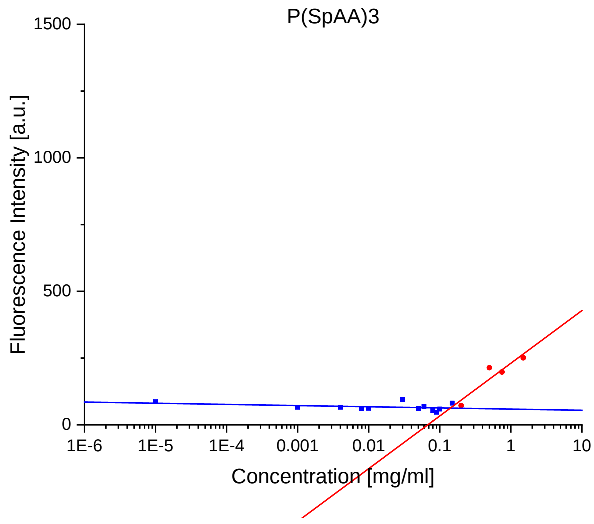

Figure S 33: Determination of critical aggregation concentration (CAC) of P(SpAA)3 with Nile red.

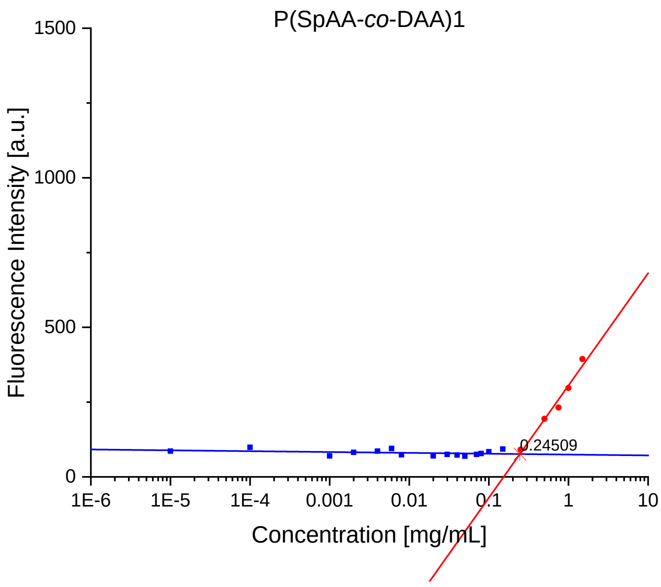

Figure S 34: Determination of critical aggregation concentration (CAC) of P(SpAA-*co*-DAA)1 with Nile red.

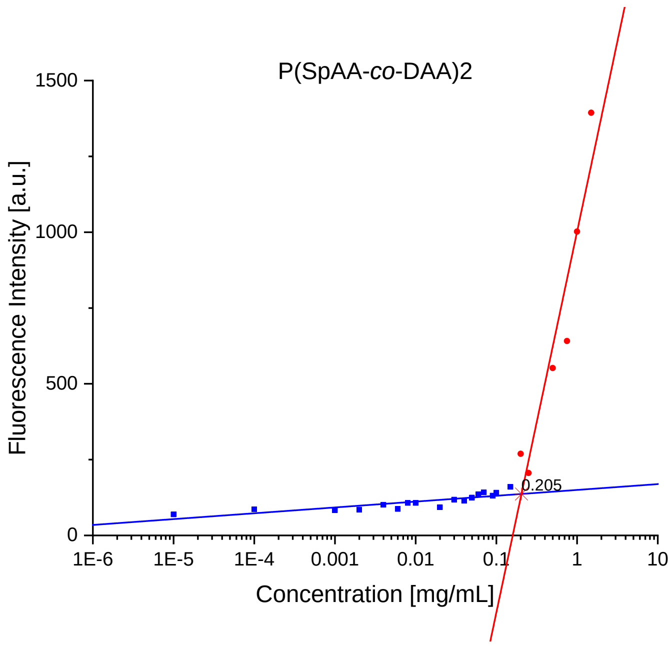

Figure S 35: Determination of critical aggregation concentration (CAC) of P(SpAA-*co*-DAA)2 with Nile red.

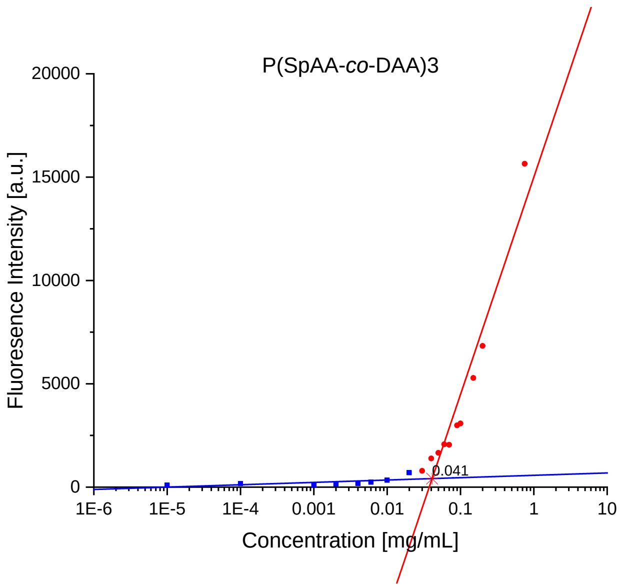

Figure S 36: Determination of critical aggregation concentration (CAC) of P(SpAA-*co*-DAA)3 with Nile red.

8. SYBR gold assay

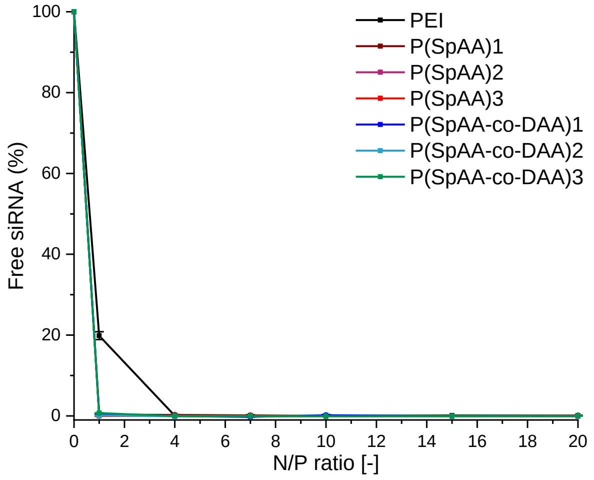

Figure S 37: siRNA encapsulation profiles of polyplexes as measured by SYBR Gold assay at various N/P ratios. 100% values are represented by the determined fluorescence of uncondensed siRNA (data points indicate mean ± SD, n = 3).

9. Size and Zeta Potential

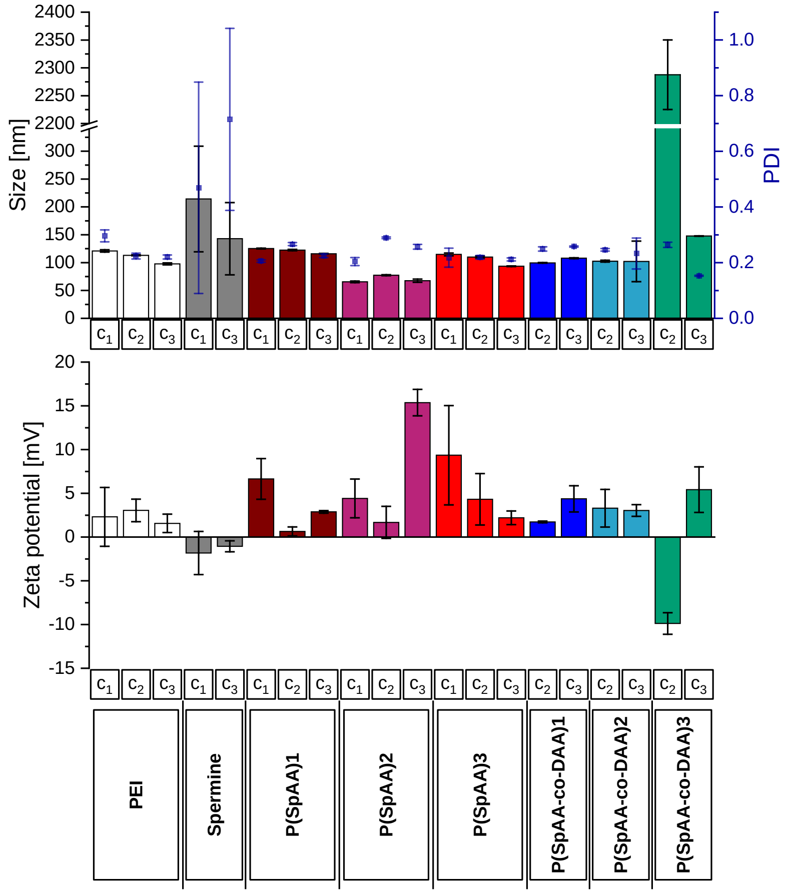

Figure S 38: Dynamic light scattering and laser Doppler anemometry measurements of polyplexes formed with PEI, Spermine, P(SpAA)1-3 or P(SpAA-*co*-DAA)1-3. (Top) Hydrodynamic diameters (left y-axis), polydispersity indices (PDI, right y-axis) and (Bottom) zeta potentials of polyplexes at concentrations of c_1 = 5.603 µg/mL, c_2 = 7.844 µg/mL, c_3 = 11.206 µg/mL polymer representing $µg_{polymer}/µg_{siRNA}$ weight ratios of 0.67, 0.93 and 1.33 assigned to N/P ratios of 5, 7 and 10 for PEI (data points indicate mean ± SD, n = 3).

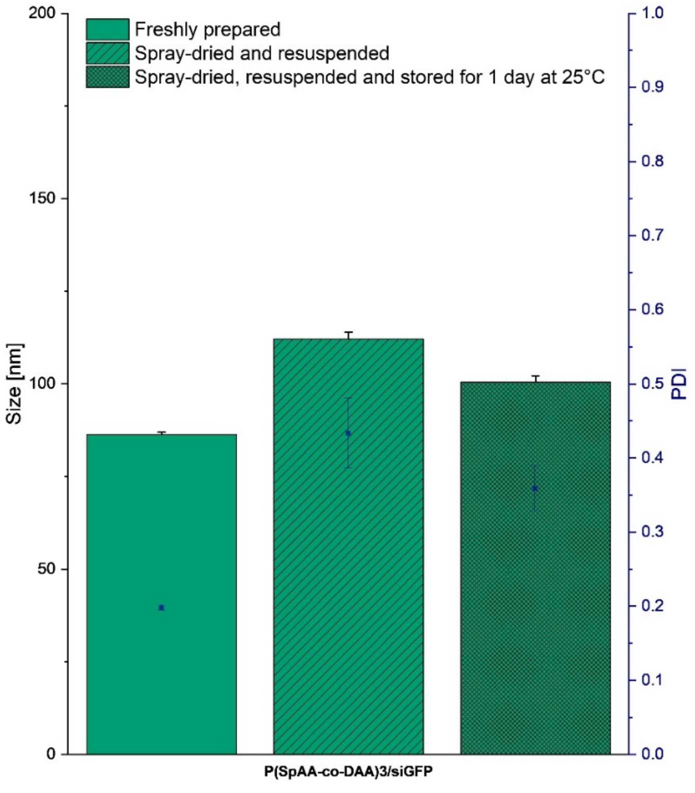

Figure S 39: Hydrodynamic diameters (left y-axis), polydispersity indices (PDI, right y-axis) of polyplexes at N/P ratio of 5 (data points indicate mean ± SD, n = 3).

10. Heparin SYBR gold assay

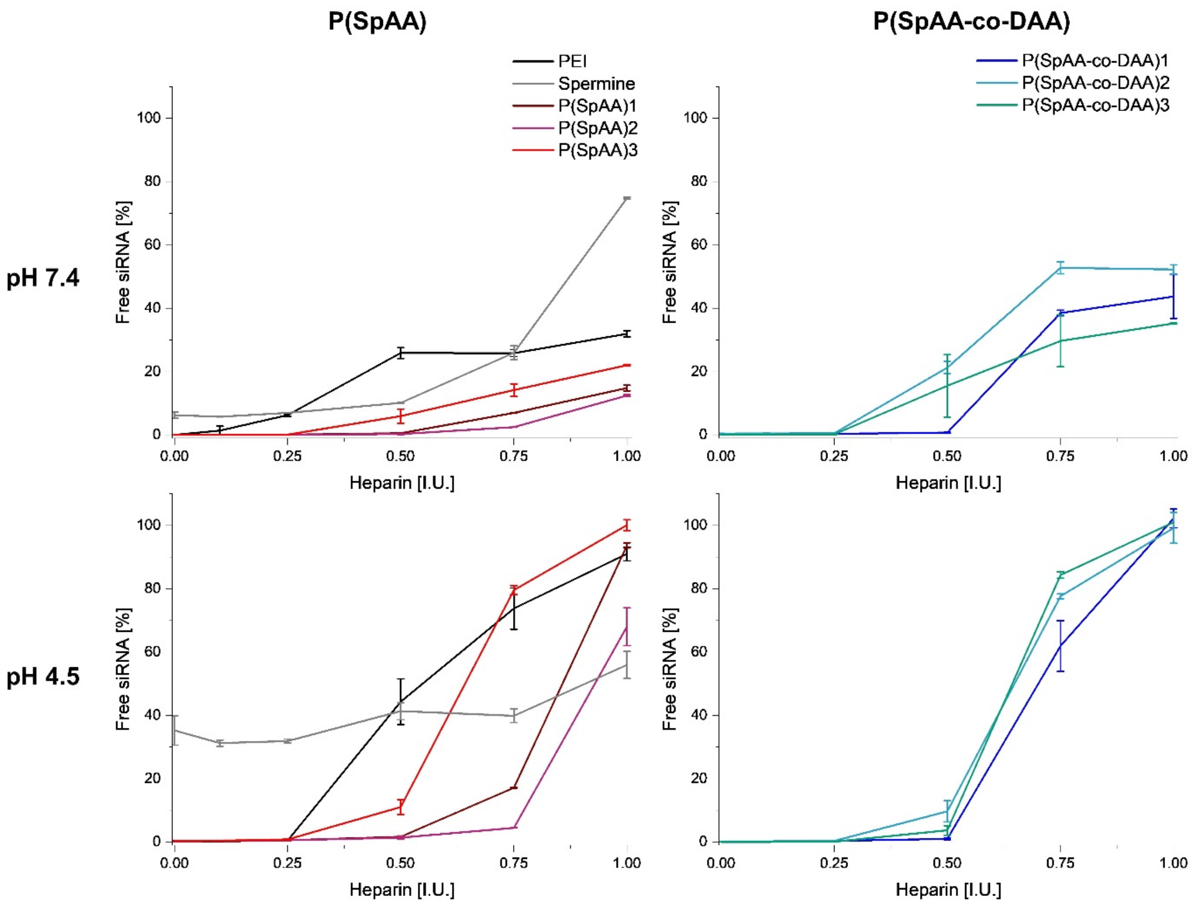

Figure S 40: Release profiles of siRNA from PEI, Spermine, P(SpAA) 1-3 and P(SpAA-co-DAA) 1-3 polyplexes at N/P 7 as a function of heparin concentration (0.0 - 1.0 I.U. heparin per well, 1 I.U. = 4.9 µg) at pH 7.4 (top) and pH 4.5 (bottom).

11. Cellular uptake

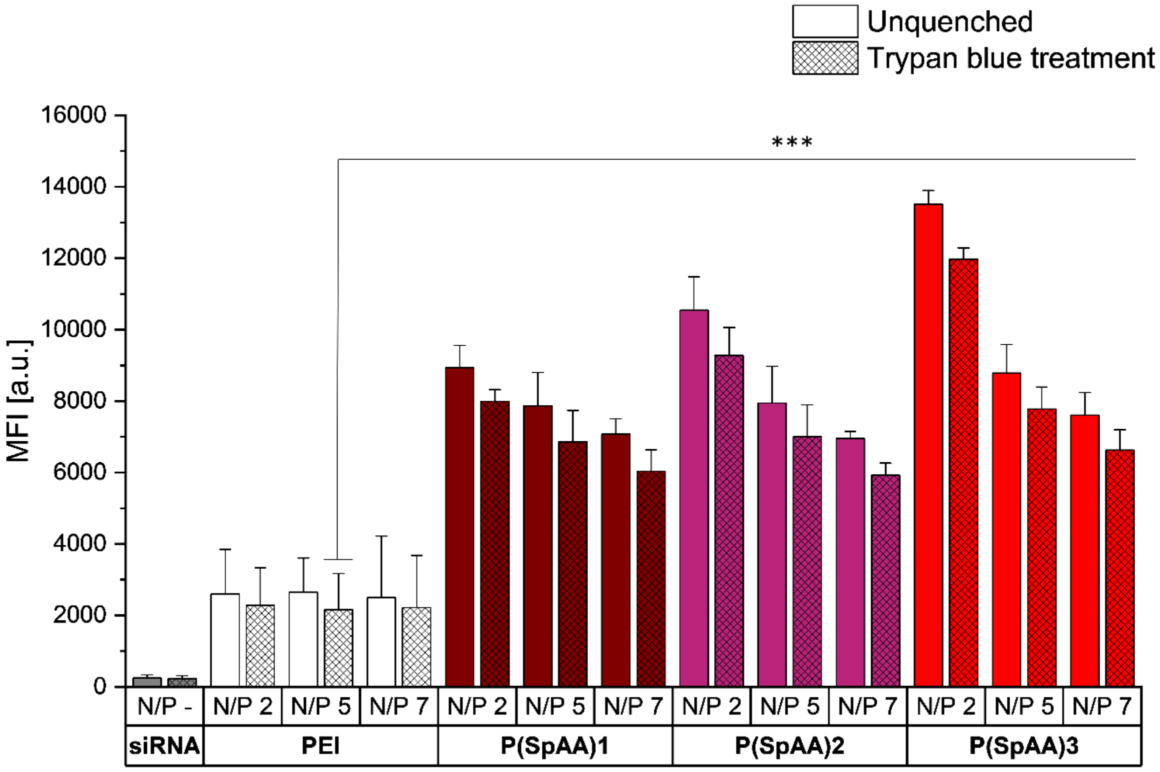

Figure S 41: Cellular uptake of polyplexes made of AF488-labeled siRNA with PEI or P(SpAA) 1-3 at N/P ratios of 2,5 and 7 after 24 h of incubation, as quantified by flow cytometry performed with and without trypan quenching and presented as median fluorescence intensity. Cells treated with free siRNA served as negative control. Significance levels shown in comparison to PEI uptake. Lipofectamine as positive control was omitted for clarity.

12. eGFP knockdown

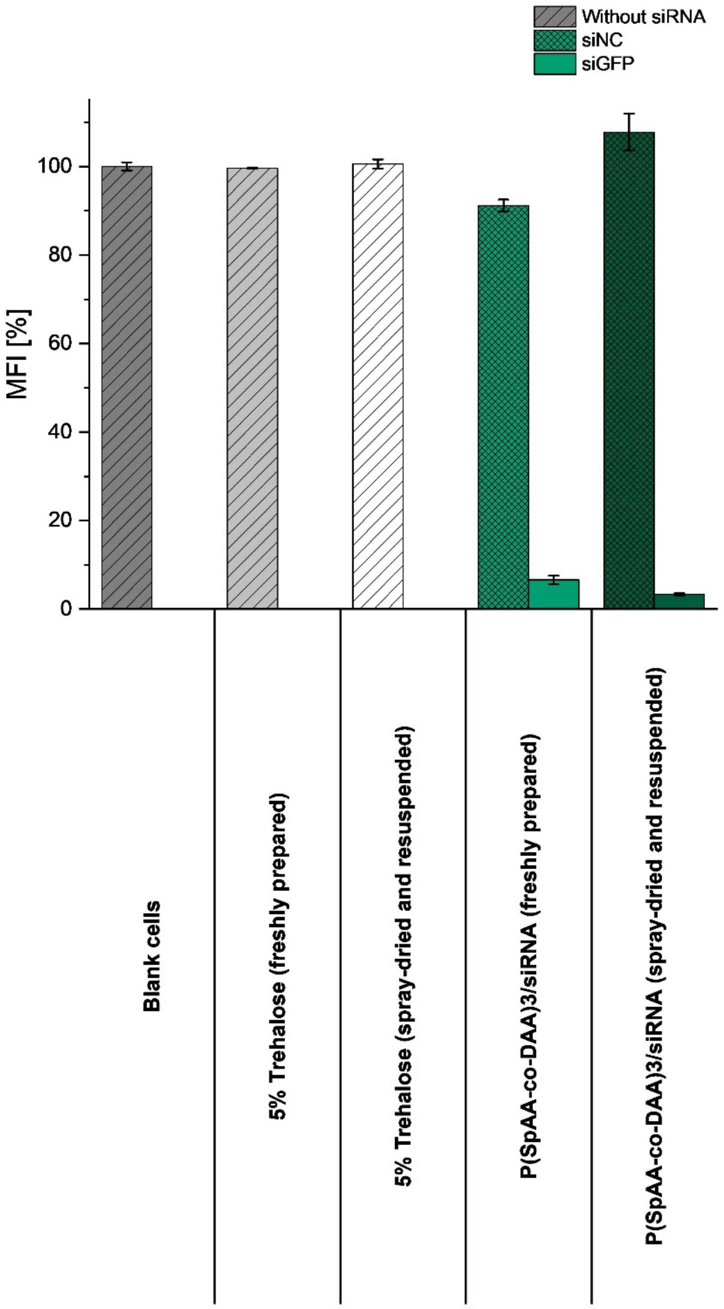

Figure S 42: Enhanced green fluorescent protein (eGFP) knockdown of P(SpAA-co-DAA)3 polyplexes that were freshly-prepared and that were spray-dried with 5% trehalose in human non-small cell lung carcinoma cells expressing eGFP (H1299/eGFP) quantified by flow cytometry as median fluorescence intensity (MFI) of eGFP after transfection with polyplexes at N/P 5 with eGFP siRNA (siGFP) or scrambled control siRNA (siNC) for 48 h. Blank cells samples consisted of 500 µL medium and H1299/eGFP cells. 5% Trehalose or P(SpAA-co-DAA)3/siRNA (freshly prepared) samples consisted of 400 µL medium and 100 µL 5% trehalose or polyplex solution and H1299/eGFP cells. 5% Trehalose or P(SpAA-co-DAA)3/siRNA (spray-dried and resuspended) samples consisted of 400 µL medium and 100 µL resuspended powder and H1299/eGFP cells. Data given as MFI in %, in relation to untreated cells.

13. Phalloidin staining of Calu3 cells at ALI

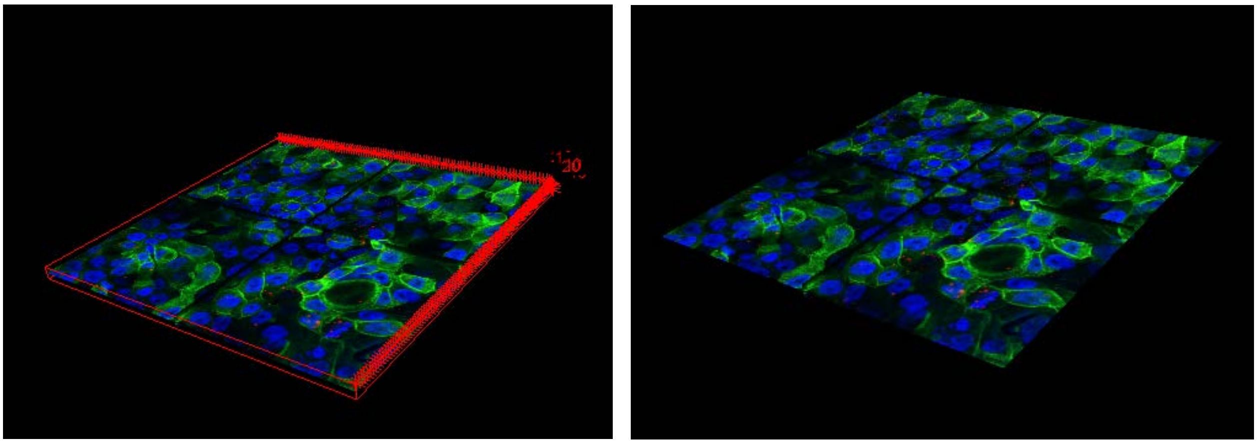

Figure S 43: 3D orthoslice of AF488-phalloidin staining of Calu3 cells cultured at ALI. Incubation time: 24 h; Blue: DAPI (nuclei); Green: Phalloidin (cytoskeleton); Red: AF647-siRNA.

14. Mucus staining of Calu3 cells at ALI

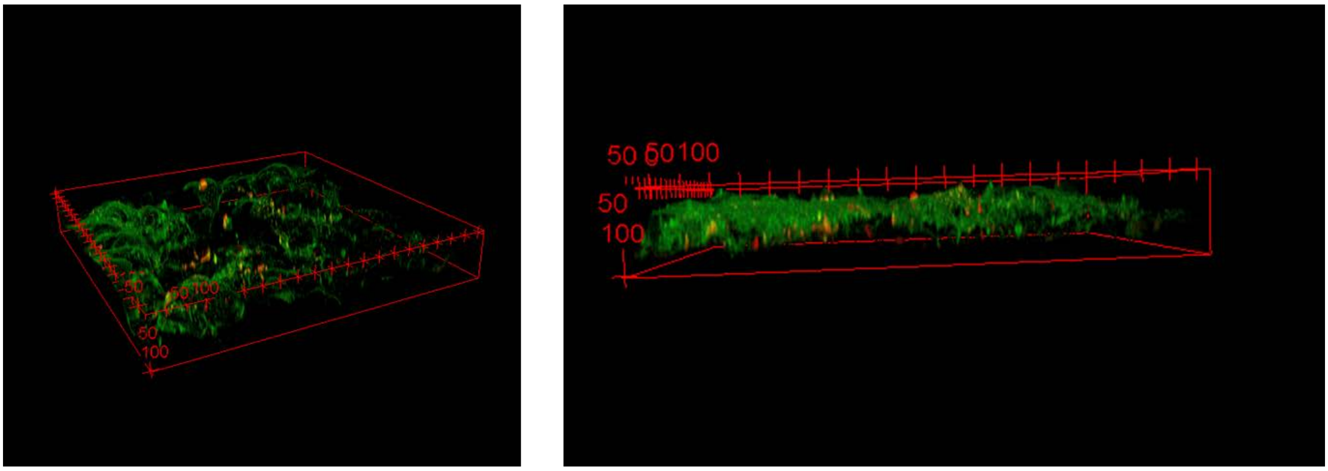

Figure S 44: 3D orthoslice from different angles of mucus staining of Calu3 cells at ALI. Incubation time: 24 h; Green: WGA-AF488 staining of mucus layer; Red: AF647-siRNA.

15. MTT Assay

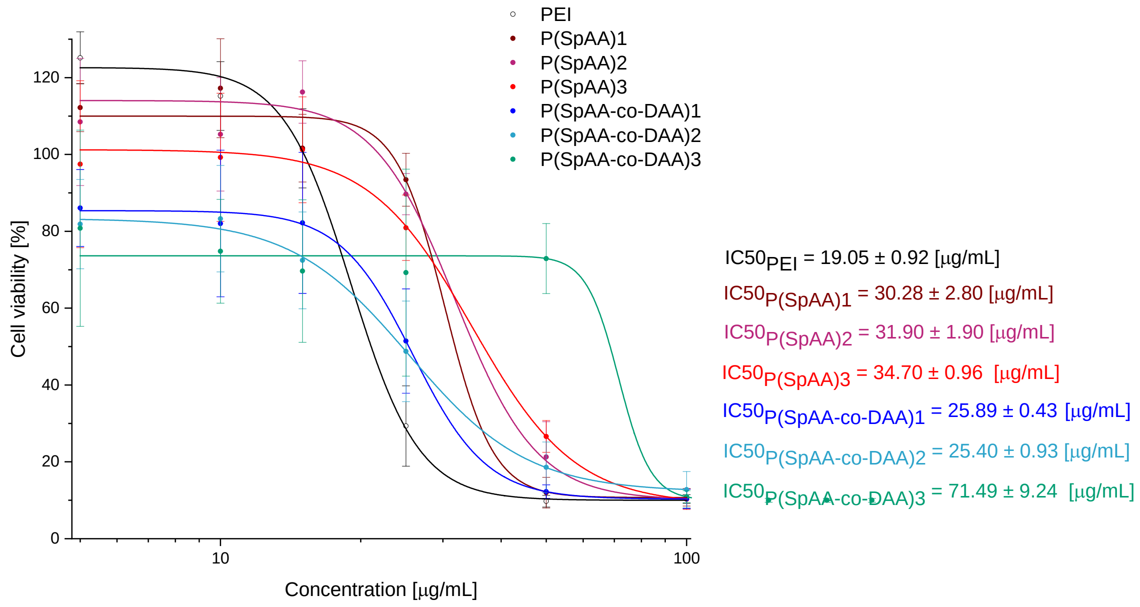

Figure S 45: IC_{50} values of PEI, P(SpAA)1-3 and P(SpAA-*co*-DAA)1-3 determined by MTT assay. Cell viabilities are plotted as a function of polymer mass concentration. IC_{50} values were calculated with a sigmoidal fit.

16. In vivo cytokine screening

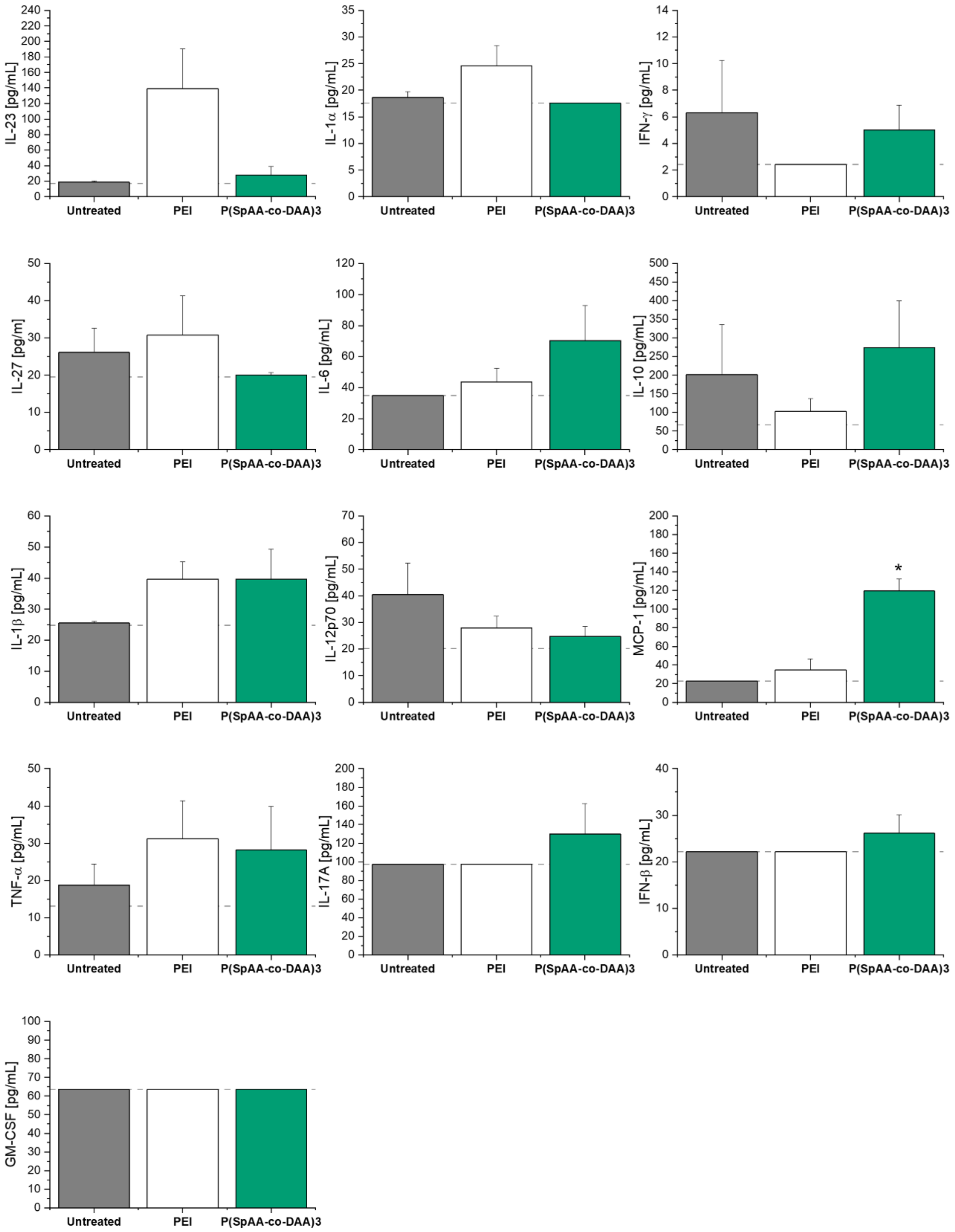

Figure S 46: Cytokine release in BALF after intratracheal instillation of siRNA polyplexes using PEI or P(SpAA-co-DAA)3 at an N/P ratio of 5. Data represent mean ± SEM (n= 4 for treatment with PEI or P(SPAA-co-DAA)3 polyplexes and n = 2 for untreated mice); significantly increased cytokine levels compared to control were marked with an asterisk (*p < 0.05). Detection limit: dotted line.

17. References

1. Lai, J. C.; Rounsfell, T.; Pittman Jr, C. U., Free‐radical homopolymerization and copolymerization of vinylferrocene. *Journal of Polymer Science Part A‐1: Polymer Chemistry* **1971,** *9* (3), 651-662.
2. Sharma, S.; Basavaraju, K. C.; Singh, A. K.; Kim, D.-P., Continuous Recycling of Homogeneous Pd/Cu Catalysts for Cross-Coupling Reactions. *Org. Lett.* **2014,** *16* (15), 3974-3977.
3. Prazeres, T. J. V.; Beija, M.; Charreyre, M.-T.; Farinha, J. P. S.; Martinho, J. M. G., RAFT polymerization and self-assembly of thermoresponsive poly(N-decylacrylamide-b-N,N-diethylacrylamide) block copolymers bearing a phenanthrene fluorescent α-end group. *Polymer* **2010,** *51* (2), 355-367.
4. Geall, A. J.; Blagbrough, I. S., Homologation of Polyamines in the Rapid Synthesis of Lipospermine Conjugates and Related Lipoplexes. *Tetrahedron* **2000,** *56* (16), 2449-2460.
